# Assessment of regional economic restorability under the stress of COVID-19 using the new interval type-2 fuzzy ORESTE method

**DOI:** 10.1007/s40747-022-00928-x

**Published:** 2022-12-19

**Authors:** Hui Zhang, Hui Gao, Peide Liu

**Affiliations:** 1 School of Business, Heze University, Heze, Shandong China; 2 School of Management Science and Engineering, Shandong University of Finance and Economics, Jinan, 250014 Shandong China

**Keywords:** Regional economic restorability, COVID-19, Interval type-2 fuzzy set, ORESTE method

## Abstract

The economic implications from the COVID-19 crisis are not like anything people have ever experienced. As predictions indicated, it is not until the year 2025 may the global economy recover to the ideal situation as it was in 2020. Regions lacked of developing category is among the mostly affected regions, because the category includes weakly and averagely potential power. For supporting the decision of economic system recovery scientifically and accurately under the stress of COVID-19, one feasible solution is to assess the regional economic restorability by taking into account a variety of indicators, such as development foundation, industrial structure, labor forces, financial support and government's ability. This is a typical multi-criteria decision-making (MCDM) problem with quantitative and qualitative criteria/indicator. To solve this problem, in this paper, an investigation is conducted to obtain 14 indicators affecting regional economic restorability, which form an indicator system. The interval type-2 fuzzy set (IT2FS) is an effective tool to express experts’ subjective preference values (PVs) in the process of decision-making. First, some formulas are developed to convert quantitative PVs to IT2FSs. Second, an improved interval type-2 fuzzy ORESTE (IT2F-ORESTE) method based on distance and likelihood are developed to assess the regional economic restorability. Third, a case study is given to illustrate the method. Then, robust ranking results are acquired by performing a sensitivity analysis. Finally, some comparative analyses with other methods are conducted to demonstrate that the developed IT2F-ORESTE method can supporting the decision of economic system recovery scientifically and accurately.

## Introduction

The COVID-19 outbreak in 2020, as a major public health emergency, has brought heavy disaster to people around the world. COVID-19 pandemic is considered to be the notorious economic shock arising throughout the year 2020. The economic implications from the COVID-19 crisis are not like anything people have ever experienced. As predictions indicated, it is not until the year 2025 may the global economy recover to the ideal situation as it was in 2020 [1]. As a result, although the negative effects of the deadly CODIV-19 pandemic are still presented currently, the regional economic recovery phase must be projected to start due to the fact that regional economic development has plummeted to historic bottom [2–6]. Specially, regions lacked of developing category is among the mostly affected regions, because the category includes weakly and averagely potential power [7–9].

Quantitative research on the impact of major public health events on economic system can provide scientific support for improving the regional economic restorability. Existing studies have analyzed the economic impact of major public health events at different scales and industrial sectors, but there is still an issue that needs to be discussed:

1. There are few studies on the economic impact of epidemics at the scale and level. What’s more, there are as yet no studies in the associated field of the assessment of regional economic restorability (RER) under the stress of COVID-19.

In this study, RER can be considered as a multi-criteria (indicators) decision-making (MCDM) problem which a number of regions should be ranked based on several special indicators. Studies form China, Japan researched on establishing assessment indicator systems for major natural disaster or public health emergencies, such as earthquake [10], flood damage [11], SARS [12], etc., and then implemented in the prioritization of regions.

Those assessment indicator systems contain specific indicators for reflecting the comprehensive strength of regional economy. At the same time, each indicator is assigned a certain number to confirm its relative significance. Obviously, those systems are good at processing quantitative information, however, it still has some drawbacks. On one hand, those systems are limited in pay close attention to comprehensive indicators, which make the final ranking results less practical and effective in determining the priority for regions; on the other hand, those indicators are mostly qualitative with a great deal of fuzzy and imprecise information, but the MCDM methods mentioned earlier are not able to process qualitative information. The type-1 fuzzy set (T1FS) theory [13] represent the qualitative indicator values with membership function (MF). MFs of T1FS are crisp number and have two-dimensional NFs. Nevertheless, in the real assessment of RER, owing to more complexity and uncertainty, the preference values of most indicator, such as location advantages, foreign trade dependence, diversification of industrial structure, industrial clusters competitiveness and Internet economy development environment, etc., cannot be represented sufficiently by T1FS. Because it is unreasonable to apply an accurate membership degree for an uncertain item. In such circumstances, Type-2 fuzzy set (T2FS) [13] are developed based on T1FS which could cover more complexity and uncertainty by three-dimensional MFs. That is, T2FS can more easily express vagueness and imprecision than T1FS. Whereas, the computation of T2FS is commonly complex, and the corresponding amounts of computation are very large. As a consequence, interval type-2 fuzzy set (IT2FS) is the most extensively utilized, membership degree of IT2FS take the form of crisp intervals, which make the calculations related to IT2FS. Furtherly, many studies of IT2FS have detected that IT2FS is a very helpful tool for quantifying the ambiguous nature of linguistic variables. In this regard, the IT2FS is a suitable tool when their fuzz MF cannot be defined easily for fuzzy system.

Although MCDM methods with IT2FSs have been widely applied to many fields [14–16], there are few studies on assessment of RER applying interval type-2 fuzzy MCDM (IT2F-MCDM) methods. In the meantime, as a result of effectiveness, many IT2F-MCDM methods have also been developed, which are mostly the utility value-based methods, such as the interval type-2 fuzzy aggregate operators [14–16], the interval type-2 fuzzy TOPSIS (IT2F-TOPSIS) method [17], the interval type-2 fuzzy VIKOR (IT2F-VIKOR) method [18], and the interval type-2 fuzzy MULTIMOORA (IT2F- MULTIMOORA) method [19]. However, the existing IT2F-MCDM methods have some crucial drawbacks:

(2) The above IT2F-MCDM methods only focus on the preference interrelations and the indifference interrelations between alternatives. And the incomparable interrelations are neglected which occurs objectively. For instance, when a comparison of economic restorability according to diversification of industrial structure ($\tilde{C}_{1}$) and industrial clusters competitiveness ($\tilde{C}_{2}$) is given between regions ($X_{1}$ and $X_{2}$), if in region $X_{1}$ the preference value (PV) of $\tilde{C}_{1}$ is “Very unimportant” but the PV of $\tilde{C}_{2}$ is “Very important”, and if in region $X_{2}$ the preference value (PV) of $\tilde{C}_{1}$ is “Very important” but the PV of $\tilde{C}_{2}$ is “Very unimportant”, then these two regions cannot be regarded simply indifference interrelation based on the corresponding aggregated results.
(3) The existing IT2F-MCDM methods can merely solve the decision-making problems that the PVs are represented as IT2FSs but cannot solve the real problems that an unspecified number of the PVs are in crisp numbers. But in reality, the cases involve generally both quantitative indicators and qualitative indicators, and under the most circumstances the quantitative indicator PVs are easy to acquire.

The ORESTE method, developed originally by Roubens [20], is an ordinary outranking method and does not need to be concerned with crisp indicator weights. Compared with the existing MCDM methods, the ORESTE method not only can determine the utility values of alternatives but also can capture the preference interrelations, incomparability interrelations and the indifference interrelations between alternatives. Moreover, a large number of researchers have developed some extended forms of the OREST method, such as probabilistic hesitant fuzzy ORESTE method [21], hesitant fuzzy linguistic ORESTE method [22], interval type-2 fuzzy ORESTE (IT2F-ORESTE) method [23]. Although the IT2F-ORESTE method can overcome the above drawback (2), it still has some other drawbacks:

(4) Most of the distance measures (DMs) for IT2FS are generalizations of the distances applied in the crisp sets, using the membership function to take place of the characteristic function, such as the normalized Hamming DM and the normalized Euclidean DM. Heidarzade et al. [24] illustrated that these two DMs are not suitable for IT2FS and require extensive computations.
(5) The likelihood of IT2FSs has not been combined with the IT2F-MCDM methods. The measure of preference information (PI) has always been a hot button for IT2F-MCDM method improvement. Different measures of IT2FSs have a critical impact on the ordering of schemes on account of different information they conveyed. For instance, the similarity of IT2FSs can detail the general interrelation between PI [25], and the entropy of IT2FSs can detail the uncertainty of PI [26]. Compared to similarity and entropy, the likelihood of IT2FSs can detail the binary interrelation of PI. Besides, it still has some wonderful properties such as transmission and complementation.

Therefore, it is worthwhile developing a feasible IT2F-ORESTE solution to a significance problem in economic management field, namely, assessment of RER. First, the Delphi approach is applied to construct a comprehensive indicator system for RER based on the interview with 35 magisterial and accomplished experts from regional economic field, government management field, medical care and public health field. The IT2FSs provided by experts are applied to express fuzzy and imprecise information. Then, an improved IT2F-ORESTE method is developed to solve the RER assessment problem with both qualitative and quantitative indicator. The main contributions of this paper are summarized as follows:

- For supporting the decision of economic system recovery scientifically and accurately, on the basis of the development foundation, industrial structure, labor forces, financial support and government's ability, etc., RER of different regions under the stress of COVID-19 are determined. Thus, the drawback (1) is overcome.
- Some formulas are developed to convert quantitative PVs to IT2FSs for combining the quantitative and qualitative indicator information. In this case, drawback (3) is overcome.
- The vertex method for DM is extended to encompass IT2FSs. The extended vertex method is an efficient simple formula that requires few computations in contrast to other DMs. This overcomes drawback (4) of existing DMs.
- An improved IT2F-ORESTE method based on the DM and likelihood of IT2FS is developed to deal with the RER assessment problem. Thus, the drawback (2) and (5) are overcome.
- Also, a comprehensive discussion between the improved IT2F-ORESTE method, the traditional ORESTE method and two representative IT2F-MCDM methods, including IT2F-TOPSIS method, IT2F-VIKOR method, and IT2F-MULTIMOORA method, are developed to demonstrate the validity and reliability of the improved IT2F-ORESTE method. In addition, the case study presents a helpful reference for government departments to improve the RER.

The structure of this paper is briefly introduced as follows. “Literature reviews” constructs an assessment indicator system of RER under the stress of COVID-19. “Preliminaries” reviews some relative principal theory of IT2FS and the classic ORESTE method. “Assessment of RER under the stress of COVID-19 using the new IT2F-ORESTE method based on distance and likelihood” develops a new IT2F-ORESTE method based on distance measure and likelihood. “A case study: the assessment of RER of cities under the stress of COVID-19 epidemic” proposes a case study of the assessment of RER of Shandong Province under the stress COVID-19 epidemic. Moreover, sensitivity and comparative analyses are conducted. “Conclusions” provides the conclusions and recommendations for future study.

## Literature reviews

Indicator system and selection of MCDM method are the essential issues of assessment of RER. Thus, the literature in this section includes restorability assessment, indicator system and ORESTE method.

### Assessment of restorability

On account of the newly increased popularity of restorability in various research disciplines, some assessment methods of restorability have been proposed. Moslehi and Reddy [27] proposed a new performance-based method for characterizing and assessing resilience of multi-functional demand-side engineered systems. Liu et al. [28] presented a planning-oriented resilience assessment framework to develop quantitative resilience indices from both the system and component perspectives. Zarei et al. [29] presented a framework for resilience assessment in process systems using a fuzzy hybrid MCDM model. Abbasnejadfard et al. [30] developed a novel deterministic and probabilistic resilience assessment measures for engineering and infrastructure systems based on the economic impacts. Rezvani et al. [31] built an enhancing urban resilience evaluation system through automated rational and consistent decision-making simulations. In this study, assessment of regional economic restorability can be considered as a MCDM problem. Although MCDM methods have been widely applied to restorability assessment fields, there are few studies on assessment of RER.

### Assessment indicator system of RER under the stress of COVID-19

First of all, “assessment”, “regional economic”, “restorability”, “resilience”, “major public emergencies” are taken as keywords to search the relevant literatures in Web of Science, Science Direct, Springer Databases, Wiley Online Library and CNKI (Time is up to June 30, 2022). A great deal of literature works regarding the indicator system for RER were reviewed, which are displayed in Table 1. Clearly, assessment of RER is basis of a series of qualitative and quantitative criteria. Whereas, under the stress of COVID-19, many researchers consider the assessment indicator system should contain specific indicators for reflecting the comprehensive strength of regional economy. At present, in the context of COVID-19, government departments are required to formulate the economic promotion policies according to the RER. On this basis, the related 23 indicators from the relevant literatures are picked out. Furtherly, these indicators are divided into five dimensions from the perspectives of social and economic, which contain development foundation ($\tilde{T}_{1}$), industrial structure ($\tilde{T}_{2}$), labor forces ($\tilde{T}_{3}$), financial support ($\tilde{T}_{4}$) and government's ability ($\tilde{T}_{5}$) displayed in Table 1.

**Table 1:** Admission indicator for general RER under the stress of COVID-19

Dimensions	Original indicators	References/expert interview
Development foundation ($\tilde{T}_{1}$)	Regional GDP ($\tilde{C}_{1}$)	[32, 33]
	Location advantages ($\tilde{C}_{2}$)	[34, 35]
	Foreign trade dependence ($\tilde{C}_{3}$)	[2, 4, 36, 37]
	New infrastructure investment ($\tilde{C}_{4}$)	[2, 38, 39]
Industrial structure ($\tilde{T}_{2}$)	Diversification of industrial structure ($\tilde{C}_{5}$)	[5, 8, 40, 41]
	Industrial chain system ($\tilde{C}_{6}$)	[3, 5–7]
	Industrial clusters competitiveness ($\tilde{C}_{7}$)	[8, 42]
	R&D investment in high and new technology industries ($\tilde{C}_{8}$)	[40]
	Transformation of digital economy ($\tilde{C}_{9}$)	[9, 43]
	Internet economy development environment ($\tilde{C}_{10}$)	[44, 45]
	Small and medium-sized enterprises develop vitality ($\tilde{C}_{11}$)	[46, 47]
Labor forces ($\tilde{T}_{3}$)	Scientific and technological innovation talent resources allocation efficiency ($\tilde{C}_{12}$)	[9, 10, 28, 29]
	Unemployment rate ($\tilde{C}_{13}$)	[48]
	Introducing and training of the high level and the high-quality talents ($\tilde{C}_{14}$)	[46–48]
Financial support ($\tilde{T}_{4}$)	Intensity of credit support ($\tilde{C}_{15}$)	[49, 50]
	Per capita fiscal expenditure ($\tilde{C}_{16}$)	[51]
	Optimization of financial structure ($\tilde{C}_{17}$)	[9–11]
	Financial services industry agglomeration ($\tilde{C}_{18}$)	[33–35]
	Financial regulation policy ($\tilde{C}_{19}$)	[52, 53]
Government's ability ($\tilde{T}_{5}$)	Epidemic prevention and control efforts ($\tilde{C}_{20}$)	[2–4]
	Epidemic prevention and control effectiveness ($\tilde{C}_{21}$)	[2–4]
	Intensity of economic stimulus ($\tilde{C}_{22}$)	[1, 5–7]
	Government financial self-sufficient capacity ($\tilde{C}_{23}$)	[2–6]

### OREST method

The ORESTE method is an ordinary outranking method to deal with MCDM problems. The most interesting part of ORESTE method is to separate preference, indifference and incomparability relations of alternatives through the conflict analysis, which makes the results more easily accepted by the decision-makers. At present, a large number of researchers have developed some extended forms of the OREST method. Li et al. [21] proposed an ORESTE method for MCDM with probabilistic hesitant fuzzy. Li et al. [22] prioritized the elective surgery patient admission in a Chinese public tertiary hospital using the hesitant fuzzy linguistic ORESTE method. Zheng et al. [23] developed an extended IT2F-ORESTE method for risk analysis in FMEA. Liao et al. [54] presented a new hesitant fuzzy linguistic ORESTE method for hybrid MCDM. Luo et al. [55] proposed a likelihood-based hybrid ORESTE method for evaluating the thermal comfort in underground mines. Wang et al. [56] proposed a double hierarchy hesitant fuzzy linguistic ORESTE method. Wang et al. [57] developed an interval 2-Tuple linguistic Fine-Kinney model for risk analysis based on extended ORESTE method with cumulative prospect theory. Liang et al. [58] proposed a hesitant Pythagorean fuzzy ORESTE method to determine the risk priority of the failure modes.

These previous studies indicate that the ORESTE method has been successfully utilized to address the priority calculation problem. Consequently, in this study, it is worthwhile developing the classic ORESTE method and extending it into the interval type-2 fuzzy context to deal with the complexity MCDM problems with both qualitative and quantitative criteria and the weights being unknown.

## Preliminaries

In following subsection, some concepts, operational laws, likelihood of IT2FS, PA operator of IT2FS, and the classic ORESTE method are briefly reviewed.

### IT2FS

#### Definition 1

[14] Let $E$ be the universe of discourse, a T2FS $A$ can be denoted as follows:

1 $$ A = \left\{ {\left( {\left( {\varepsilon ,\sigma } \right),\mu_{\rm A} \left( {\varepsilon ,\sigma } \right)} \right)\left| {\forall \varepsilon \in E,\forall \sigma \in J_{\varepsilon } \subseteq \left[ {0,1} \right]} \right.} \right\} $$

where $\mu_{\rm A} \left( {\varepsilon ,\sigma } \right)$ is called type-2 MF, $0 \le \mu_{\rm A} \left( {\varepsilon ,\sigma } \right) \le 1$ for each $\varepsilon$ and $\sigma$. In addition, the T2FS $A$ also can be denoted as follows:

2 $$ A = \int_{{\varepsilon \in {\rm E}}} {\int_{{\sigma \in J_{\varepsilon } }} {\mu_{A} \left( {\varepsilon ,\sigma } \right)} } /\left( {\varepsilon ,\sigma } \right) = \int_{{\varepsilon \in {\rm E}}} {\left( {\int_{{\sigma \in J_{\varepsilon } }} {\mu_{A} \left( {\varepsilon ,\sigma } \right)} /\sigma } \right)} /\varepsilon , $$

where $J_{\varepsilon } \subseteq \left[ {0,1} \right]$ is the primary membership at $\varepsilon$ and $\int_{{\sigma \in J_{\varepsilon } }} {\mu_{A} \left( {\varepsilon ,\sigma } \right)} /\sigma$ is the second membership at $\varepsilon$. For discrete spaces, $\int {}$ is replaced by $\sum {}$.

#### Definition 2

[14] Let $A$ be a T2FS in the universe of discourse $E$, if all $\mu \left( {\varepsilon ,\sigma } \right) = 1$, then $A$ is called an IT2FS, represented as follows:

3 $$ A = \int_{{\varepsilon \in {\rm E}}} {\int_{{\sigma \in J_{\varepsilon } }} 1 } /\left( {\varepsilon ,\sigma } \right) = \int_{{\varepsilon \in {\rm E}}} {\left( {\int_{{\sigma \in J_{\varepsilon } }} 1 /\sigma } \right)} /\varepsilon . $$

Apparently, IT2FS $A$ in $E$ is totally determined by the footprint of uncertainty (FOU) which can be denoted:

4 $$ FOU\left( A \right) = \bigcup\nolimits_{\varepsilon \in E} {J_{\varepsilon } } = \bigcup\nolimits_{\varepsilon \in E} {\left\{ {\left( {\varepsilon ,\sigma } \right)\left| {\sigma \in J_{\varepsilon } \subseteq \left[ {0,1} \right]} \right.} \right\}} . $$

Generally, due to the calculations on IT2FSs are more complex, some simplified forms can be utilized to denote IT2FSs. In here, we utilize trapezoidal IT2FS to process GSS problems.

#### Definition 3

**Fig. 1:**
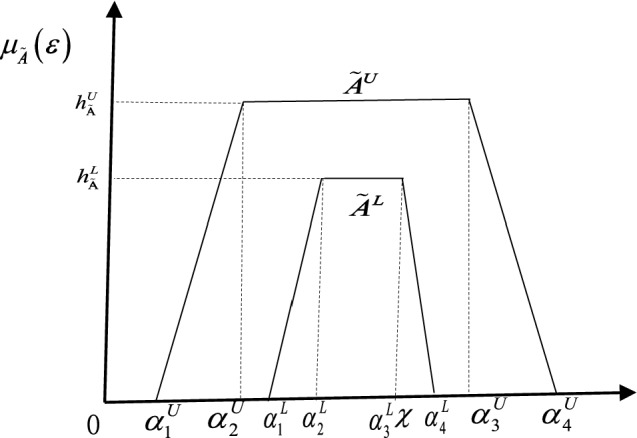
A geometrical interpretation of an IT2FS [23, 24]

[14] Let $\tilde{A}^{L}$ and $\tilde{A}^{U}$ be two generalized trapezoidal fuzzy numbers, where the height of a generalized FM is positioned in $\left[ {0,1} \right]$. Let $h_{{\tilde{A}}}^{L}$ and $h_{{\tilde{A}}}^{U}$ be the heights of $\tilde{A}^{L}$ and $\tilde{A}^{U}$, respectively. An IT2FS $\tilde{A}$ (as shown in Fig. 1) in the universe of discourse $E$ can be defined:

$$ \tilde{A} = \left( {\tilde{A}^{L} ,\tilde{A}^{U} } \right) = \left[ {\left( {\alpha_{1}^{L} ,\alpha_{2}^{L} ,\alpha_{3}^{L} ,\alpha_{4}^{L} ;h_{{\tilde{A}}}^{L} } \right),\left( {\alpha_{1}^{U} ,\alpha_{2}^{U} ,\alpha_{3}^{U} ,\alpha_{4}^{U} ;h_{{\tilde{A}}}^{U} } \right)} \right] $$

where $\tilde{A}^{L}$ and $\tilde{A}^{U}$ are type-1 fuzzy sets,$\alpha_{1}^{L} \le \alpha_{2}^{L} \le \alpha_{3}^{L} \le \alpha_{4}^{L}$,$\alpha_{1}^{U} \le \alpha_{2}^{U} \le \alpha_{3}^{U} \le \alpha_{4}^{U}$, $\alpha_{1}^{U} \le \alpha_{1}^{L}$, $\alpha_{4}^{L} \le \alpha_{4}^{U}$ and $0 \le h_{{\tilde{A}}}^{L} \le h_{{\tilde{A}}}^{U} \le 1$. The lower MF $\tilde{A}^{L} \left( \varepsilon \right)$ and upper MF $\tilde{A}^{U} \left( \varepsilon \right)$ are defined as follows:

5 $$ \tilde{A}^{L} \left( \varepsilon \right) = \left\{ {\begin{array}{*{20}c} {h_{{\tilde{A}}}^{L} \frac{{\left( {\varepsilon - \alpha_{1}^{L} } \right)}}{{\alpha_{2}^{L} - \alpha_{1}^{L} }} \alpha_{1}^{L} \le \varepsilon \le \alpha_{2}^{L} } \\ { h_{{\tilde{A}}}^{L} \alpha_{2}^{L} \le \varepsilon \le \alpha_{3}^{L} } \\ {h_{{\tilde{A}}}^{L} \frac{{\left( {\alpha_{4}^{L} - \varepsilon } \right)}}{{\alpha_{4}^{L} - \alpha_{3}^{L} }} \alpha_{1}^{L} \le \varepsilon \le \alpha_{2}^{L} } \\ {0 {\text{otherwise}}} \\ \end{array} } \right.\,\tilde{A}^{U} \left( \varepsilon \right) = \left\{ {\begin{array}{*{20}c} {h_{{\tilde{A}}}^{U} \frac{{\left( {\varepsilon - \alpha_{1}^{U} } \right)}}{{\alpha_{2}^{L} - \alpha_{1}^{L} }} \alpha_{1}^{U} \le \varepsilon \le \alpha_{2}^{U} } \\ { h_{{\tilde{A}}}^{U} \alpha_{2}^{U} \le \varepsilon \le \alpha_{3}^{U} } \\ {h_{{\tilde{A}}}^{U} \frac{{\left( {\alpha_{4}^{U} - \varepsilon } \right)}}{{\alpha_{4}^{L} - \alpha_{3}^{L} }} \alpha_{1}^{U} \le \varepsilon \le \alpha_{2}^{U} } \\ {0 {\text{otherwise}}} \\ \end{array} } \right.. $$

#### Definition 4

[15, 16] Let $\tilde{A}_{1} = \Bigl( \tilde{A}_{1}^{L} ,\tilde{A}_{1}^{U}  \Bigr) = \Bigl[ \Bigl( \alpha_{11}^{L} ,\alpha_{12}^{L} ,\alpha_{13}^{L} ,\alpha_{14}^{L} ;h_{{\tilde{A}}}^{L}  \Bigr),\Bigl( \alpha_{11}^{U} ,\alpha_{12}^{U} ,\alpha_{13}^{U} ,\alpha_{14}^{U} ;h_{{\tilde{A}}}^{U}  \Bigr) \Bigr]$ and $\tilde{A}_{2} = \left( {\tilde{A}_{2}^{L} ,\tilde{A}_{2}^{U} } \right) = \left[ {\left( {\alpha_{21}^{L} ,\alpha_{22}^{L} ,\alpha_{23}^{L} ,\alpha_{24}^{L} ;h_{{\tilde{A}_{2} }}^{L} } \right),\left( {\alpha_{21}^{U} ,\alpha_{22}^{U} ,\alpha_{23}^{U} ,\alpha_{24}^{U} ;h_{{\tilde{A}_{2} }}^{U} } \right)} \right]$ be any two IT2FSs, then the operational laws between $\tilde{A}_{1}$ and $\tilde{A}_{2}$ are defined as follows:

6 $$ \tilde{A}_{1} \oplus \tilde{A}_{2} = \left[ \begin{gathered} \left( {\alpha_{11}^{L} + \beta_{21}^{L} ,\alpha_{12}^{L} + \beta_{22}^{L} ,\alpha_{13}^{L} + \beta_{23}^{L} ,\alpha_{14}^{L} + \beta_{24}^{L} ;\min \left\{ {h_{{\tilde{A}_{1} }}^{L} ,h_{{\tilde{A}_{2} }}^{L} } \right\}} \right), \hfill \\ \left( {\alpha_{11}^{U} + \beta_{21}^{U} ,\alpha_{12}^{U} + \beta_{22}^{U} ,\alpha_{13}^{U} + \beta_{23}^{U} ,\alpha_{14}^{U} + \beta_{24}^{U} ;\min \left\{ {h_{{\tilde{A}_{1} }}^{U} ,h_{{\tilde{A}_{2} }}^{U} } \right\}} \right) \hfill \\ \end{gathered} \right] $$

7 $$ \tilde{A}_{1} \otimes \tilde{A}_{2} = \left[ \begin{aligned} & \left( {\alpha_{11}^{L} \beta_{21}^{L} ,\alpha_{12}^{L} \beta_{22}^{L} ,\alpha_{13}^{L} \beta_{23}^{L} ,\alpha_{14}^{L} \beta_{24}^{L} ;\min \left\{ {h_{{\tilde{A}_{1} }}^{L} ,h_{{\tilde{A}_{2} }}^{L} } \right\}} \right), \hfill \\ & \left( {\alpha_{11}^{U} \beta_{21}^{U} ,\alpha_{12}^{U} \beta_{22}^{U} ,\alpha_{13}^{U} \beta_{23}^{U} ,\alpha_{14}^{U} \beta_{24}^{U} ;\min \left\{ {h_{{\tilde{A}_{1} }}^{U} ,h_{{\tilde{A}_{2} }}^{U} } \right\}} \right) \hfill \\ \end{aligned} \right] $$

8 $$ \chi \tilde{A}_{1} = \left[ {\left( {\chi \alpha_{11}^{L} ,\chi \alpha_{12}^{L} ,\chi \alpha_{13}^{L} ,\chi \alpha_{14}^{L} ;h_{{\tilde{A}_{1} }}^{L} } \right),\left( {\chi \alpha_{11}^{U} ,\chi \alpha_{12}^{U} ,\chi \alpha_{13}^{U} ,\chi \alpha_{14}^{U} ;h_{{\tilde{A}_{1} }}^{U} } \right)} \right],\chi \ge 0 $$

9 $$ \tilde{A}_{1}^{\chi } = \left[ {\left( {\left( {\alpha_{11}^{L} } \right)^{\chi } ,\left( {\alpha_{12}^{L} } \right)^{\chi } ,\left( {\alpha_{13}^{L} } \right)^{\chi } ,\left( {\alpha_{14}^{L} } \right)^{\chi } ;h_{{\tilde{A}_{1} }}^{L} } \right),\left( {\left( {\alpha_{11}^{U} } \right)^{\chi } ,\left( {\alpha_{12}^{U} } \right)^{\chi } ,\left( {\alpha_{13}^{U} } \right)^{\chi } ,\left( {\alpha_{14}^{U} } \right)^{\chi } ;h_{{\tilde{A}_{1} }}^{U} } \right)} \right],\chi \ge 0 $$

#### Definition 5

[59] Let $\tilde{A}_{1} = \left( {\tilde{A}_{1}^{L} ,\tilde{A}_{1}^{U} } \right) = \Big[ \Big( \alpha_{11}^{L} ,\alpha_{12}^{L} ,\alpha_{13}^{L} ,\alpha_{14}^{L} ;h_{{\tilde{A}_{1} }}^{L}  \Big),\Big( \alpha_{11}^{U} ,\alpha_{12}^{U} ,\alpha_{13}^{U} ,\alpha_{14}^{U} ;h_{{\tilde{A}_{1} }}^{U}  \Big) \Big]$ and $\tilde{A}_{2} = \left( {\tilde{A}_{2}^{L} ,\tilde{A}_{2}^{U} } \right) = \left[ {\left( {\alpha_{21}^{L} ,\alpha_{22}^{L} ,\alpha_{23}^{L} ,\alpha_{24}^{L} ;h_{{\tilde{A}_{2} }}^{L} } \right),\left( {\alpha_{21}^{U} ,\alpha_{22}^{U} ,\alpha_{23}^{U} ,\alpha_{24}^{U} ;h_{{\tilde{A}_{2} }}^{U} } \right)} \right]$ be two any IT2FSs, then the distance measure based on the extend vertex method between $\tilde{A}_{1}$ and $\tilde{A}_{2}$ are defined as follows:

10 $$ d\left( {\tilde{A}_{1} ,\tilde{A}_{2} } \right) = \sqrt {\frac{1}{8}\left( \begin{gathered} \left( {\alpha_{11}^{L} - \alpha_{21}^{L} } \right)^{2} + \left( {\alpha_{12}^{L} - \alpha_{22}^{L} } \right)^{2} + \left( {\alpha_{13}^{L} - \alpha_{23}^{L} } \right)^{2} + \left( {\alpha_{14}^{L} - \alpha_{24}^{L} } \right)^{2} + \left( {\alpha_{11}^{U} - \alpha_{21}^{U} } \right)^{2} + \left( {\alpha_{12}^{U} - \alpha_{22}^{U} } \right)^{2} \hfill \\ + \left( {\alpha_{13}^{U} - \alpha_{23}^{U} } \right)^{2} + \left( {\alpha_{14}^{U} - \alpha_{24}^{U} } \right)^{2} + 2\left( {h_{{\tilde{A}_{1} }}^{L} - h_{{\tilde{A}_{2} }}^{L} } \right)^{2} + 2\left( {h_{{\tilde{A}_{1} }}^{U} - h_{{\tilde{A}_{2} }}^{U} } \right)^{2} \hfill \\ \end{gathered} \right)} . $$

### Likelihood of IT2FS

In this section, a framework of the likelihood of IT2FSs based on the upper likelihood and the lower likelihood are proposed in the following definition.

#### Definition 6

[60] Let $\tilde{A}_{1} = \left( {\tilde{A}_{1}^{L} ,\tilde{A}_{1}^{U} } \right) = \Big[ \Big( \alpha_{11}^{L} ,\alpha_{12}^{L} ,\alpha_{13}^{L} ,\alpha_{14}^{L} ;h_{{\tilde{A}_{1} }}^{L}  \Big),\Big( \alpha_{11}^{U} ,\alpha_{12}^{U} ,\alpha_{13}^{U} ,\alpha_{14}^{U} ;h_{{\tilde{A}_{1} }}^{U}  \Big) \Big]$ and $\tilde{A}_{2} = \left( {\tilde{A}_{2}^{L} ,\tilde{A}_{2}^{U} } \right) = \left[ {\left( {\alpha_{21}^{L} ,\alpha_{22}^{L} ,\alpha_{23}^{L} ,\alpha_{24}^{L} ;h_{{\tilde{A}_{2} }}^{L} } \right),\left( {\alpha_{21}^{U} ,\alpha_{22}^{U} ,\alpha_{23}^{U} ,\alpha_{24}^{U} ;h_{{\tilde{A}_{2} }}^{U} } \right)} \right]$ be two any IT2FSs. Assume that at least one of $h_{{\tilde{\rm A}_{1} }}^{L} \ne h_{{\tilde{\rm A}_{2} }}^{U}$, $\alpha_{14}^{L} \ne \alpha_{11}^{L}$, $\alpha_{24}^{U} \ne \alpha_{21}^{U}$, and $\alpha_{1\zeta }^{L} \ne \alpha_{2\zeta }^{U}$ holds, and at least one of $h_{{\tilde{A}_{1} }}^{U} \ne h_{{\tilde{A}_{2} }}^{L}$, $\alpha_{14}^{U} \ne \alpha_{11}^{U}$, $\alpha_{24}^{L} \ne \alpha_{21}^{L}$, and $\alpha_{1\zeta }^{U} \ne \alpha_{2\zeta }^{L}$ holds, where $\zeta = \left\{ {1,2,3,4} \right\}$. The upper likelihood $I^{ + } \left( {\tilde{A}_{1} \ge \tilde{A}_{2} } \right)$ of an IT2FS binary relation (BR) $\tilde{A}_{1} \ge \tilde{A}_{2}$ can be defined by:

11 $$ I^{ + } \left( {\tilde{A}_{1} \ge \tilde{A}_{2} } \right) = \max \left\{ {1 - \max \left[ {\frac{{\sum\nolimits_{\zeta = 1}^{4} {\max \left( {\alpha_{2\zeta }^{L} - \alpha_{1\zeta }^{U} ,0} \right)} + \left( {\alpha_{24}^{L} - \alpha_{11}^{U} } \right) + 2\max \left( {h_{{\tilde{A}_{2} }}^{L} - h_{{\tilde{A}_{1} }}^{U} ,0} \right)}}{{\sum\nolimits_{\zeta = 1}^{4} {\left| {\alpha_{2\zeta }^{L} - \alpha_{1\zeta }^{U} } \right|} + \left( {\alpha_{14}^{U} - \alpha_{11}^{U} } \right) + \left( {\alpha_{24}^{L} - \alpha_{21}^{L} } \right) + 2\left| {h_{{\tilde{A}_{2} }}^{L} - h_{{\tilde{A}_{1} }}^{U} } \right|}},0} \right],0} \right\} $$

The lower likelihood $I^{ - } \left( {\tilde{A}_{1} \ge \tilde{A}_{2} } \right)$ of an IT2FS BR $\tilde{A}_{1} \ge \tilde{A}_{2}$ can be defined by:

12 $$ I^{ - } \left( {\tilde{A}_{1} \ge \tilde{A}_{2} } \right) = \max \left\{ {1 - \max \left[ {\frac{{\sum\nolimits_{\zeta = 1}^{4} {\max \left( {\alpha_{2\zeta }^{U} - \alpha_{1\zeta }^{L} ,0} \right)} + \left( {\alpha_{24}^{U} - \alpha_{11}^{L} } \right) + 2\max \left( {h_{{\tilde{A}_{2} }}^{U} - h_{{\tilde{A}_{1} }}^{L} ,0} \right)}}{{\sum\nolimits_{\zeta = 1}^{4} {\left| {\alpha_{2\zeta }^{U} - \alpha_{1\zeta }^{L} } \right|} + \left( {\alpha_{14}^{L} - \alpha_{11}^{L} } \right) + \left( {\alpha_{24}^{U} - \alpha_{21}^{U} } \right) + 2\left| {h_{{\tilde{A}_{2} }}^{U} - h_{{\tilde{A}_{1} }}^{L} } \right|}},0} \right],0} \right\} $$

The likelihood $I\left( {\tilde{A}_{1} \ge \tilde{A}_{2} } \right)$ of an IT2FS BR $\tilde{A}_{1} \ge \tilde{A}_{2}$ can be defined by:

13 $$ I\left( {\tilde{A}_{1} \ge \tilde{A}_{2} } \right) = \frac{{I^{ + } \left( {\tilde{A}_{1} \ge \tilde{A}_{2} } \right) + I^{ - } \left( {\tilde{A}_{1} \ge \tilde{A}_{2} } \right)}}{2}. $$

#### Definition 7

[61] Let $\tilde{A}_{1} = \left( {\tilde{A}_{1}^{L} ,\tilde{A}_{1}^{U} } \right)$ and $\tilde{A}_{2} = \left( {\tilde{A}_{2}^{L} ,\tilde{A}_{2}^{U} } \right)$ be two any IT2FSs. Based on the likelihood, the comparison rules between $\tilde{A}_{1}$ and $\tilde{A}_{2}$ can be defined by:

1. if $I\left( {\tilde{A}_{1} \ge \tilde{A}_{2} } \right) = 1$, then $\tilde{A}_{1}$ is strictly preferred to $\tilde{A}_{2}$, denoted by $\tilde{A}_{1} \succ_{S} \tilde{A}_{2}$;
2. if $0.5 < I\left( {\tilde{A}_{1} \ge \tilde{A}_{2} } \right) < 1$, then $\tilde{A}_{1}$ is weakly preferred to $\tilde{A}_{2}$, denoted by $\tilde{A}_{1} \succ_{W} \tilde{A}_{2}$;
3. if $I\left( {\tilde{A}_{1} \ge \tilde{A}_{2} } \right) = 0.5$, then $\tilde{A}_{1}$ is indifferent to $\tilde{A}_{2}$, denoted by $\tilde{A}_{1} \sim \tilde{A}_{2}$.

### Power average operator of IT2FS

Power average operator, developed firstly by Yager [62], can be often seen as an effective technique to aggregate individual preference information. Then, the power average operator of IT2FS is developed in the following section.

#### Definition 7

[23] Assume that $\tilde{A}_{\xi } = \left( {\tilde{A}_{\xi }^{L} ,\tilde{A}_{\xi }^{U} } \right) = \left[ {\left( {\alpha_{\xi 1}^{L} ,\alpha_{\xi 2}^{L} ,\alpha_{\xi 3}^{L} ,\alpha_{\xi 4}^{L} ;h_{{\tilde{A}_{\xi } }}^{L} } \right),\left( {\alpha_{\xi 1}^{U} ,\alpha_{\xi 2}^{U} ,\alpha_{\xi 3}^{U} ,\alpha_{\xi 4}^{U} ;h_{{\tilde{A}_{\xi } }}^{U} } \right)} \right]$, $\left( {\xi = 1,2, \ldots M} \right)$ be a collection of IT2FSs. Then, the collective value of interval type-2 fuzzy power average (IT2FPA) operator is still an IT2FS, and

14 $$ \begin{gathered} IT2FPA\left( {\tilde{A}_{1} ,\tilde{A}_{2} , \cdots ,\tilde{A}_{M} } \right) \hfill \\ = \left[ \begin{gathered} \left( {\frac{{\sum\nolimits_{\xi = 1}^{M} {\left( {1 + T\left( {\tilde{A}_{\xi } } \right)} \right)\alpha_{\xi 1}^{L} } }}{{\sum\nolimits_{\xi = 1}^{M} {\left( {1 + T\left( {\tilde{A}_{\xi } } \right)} \right)} }},\frac{{\sum\nolimits_{\xi = 1}^{M} {\left( {1 + T\left( {\tilde{A}_{\xi } } \right)} \right)\alpha_{\xi 2}^{L} } }}{{\sum\nolimits_{\xi = 1}^{M} {\left( {1 + T\left( {\tilde{A}_{\xi } } \right)} \right)} }},\frac{{\sum\nolimits_{\xi = 1}^{M} {\left( {1 + T\left( {\tilde{A}_{\xi } } \right)} \right)\alpha_{\xi 3}^{L} } }}{{\sum\nolimits_{\xi = 1}^{M} {\left( {1 + T\left( {\tilde{A}_{\xi } } \right)} \right)} }},\frac{{\sum\nolimits_{\xi = 1}^{M} {\left( {1 + T\left( {\tilde{A}_{\xi } } \right)} \right)\alpha_{\xi 4}^{L} } }}{{\sum\nolimits_{\xi = 1}^{M} {\left( {1 + T\left( {\tilde{A}_{\xi } } \right)} \right)} }};\mathop {\min }\limits_{\xi = 1,2, \cdots ,M} \left( {h_{{\tilde{A}_{\xi } }}^{L} } \right)} \right), \hfill \\ \left( {\frac{{\sum\nolimits_{\xi = 1}^{M} {\left( {1 + T\left( {\tilde{A}_{\xi } } \right)} \right)\alpha_{\xi 1}^{U} } }}{{\sum\nolimits_{\xi = 1}^{M} {\left( {1 + T\left( {\tilde{A}_{\xi } } \right)} \right)} }},\frac{{\sum\nolimits_{\xi = 1}^{M} {\left( {1 + T\left( {\tilde{A}_{\xi } } \right)} \right)\alpha_{\xi 2}^{U} } }}{{\sum\nolimits_{\xi = 1}^{M} {\left( {1 + T\left( {\tilde{A}_{\xi } } \right)} \right)} }},\frac{{\sum\nolimits_{\xi = 1}^{M} {\left( {1 + T\left( {\tilde{A}_{\xi } } \right)} \right)\alpha_{\xi 3}^{U} } }}{{\sum\nolimits_{\xi = 1}^{M} {\left( {1 + T\left( {\tilde{A}_{\xi } } \right)} \right)} }},\frac{{\sum\nolimits_{\xi = 1}^{M} {\left( {1 + T\left( {\tilde{A}_{\xi } } \right)} \right)\alpha_{\xi 4}^{U} } }}{{\sum\nolimits_{\xi = 1}^{M} {\left( {1 + T\left( {\tilde{A}_{\xi } } \right)} \right)} }};\mathop {\min }\limits_{\xi = 1,2, \cdots ,M} \left( {h_{{\tilde{A}_{\xi } }}^{U} } \right)} \right) \hfill \\ \end{gathered} \right] \hfill \\ {\text{Where}}\,T\left( {\tilde{A}_{\xi } } \right) = \sum\nolimits_{\xi = 1,\xi \ne \psi }^{M} {\left( {1 - d\left( {\tilde{A}_{\xi } ,\tilde{A}_{\psi } } \right)} \right)} \hfill \\ \end{gathered} $$

By literature [23], the IT2FPA operator has some desirable properties, for example, idempotence, boundedness and monotonicity.

### The traditional ORESTE method

In this section, the traditional ORESTE method, developed initially by Roubens [20], is one of the most effective and reliable ranking methods for handing MCDM problems. The specific procedures of this method are presented as follows:

Step 1: Aggregate global preference scores (GPS).

Assume that $R_{j}$ is the original ranking of the important degree of criterion $C_{j} \left( {j = 1,2, \cdots ,n} \right)$ and $R_{j} \left( {X_{i} } \right)$ is the original ranking of the preference value of alternative $X_{i} \left( {i = 1,2, \cdots ,m} \right)$ under criterion $C_{j}$. Then, the GPS can be aggregated with

15 $$ G\left( {X_{ij} } \right) = \sqrt {\eta \left( {R_{j} } \right)^{2} + \left( {1 - \eta } \right)\left( {R_{j} \left( {X_{i} } \right)} \right)^{2} } , $$

where $\eta \in \left[ {0,1} \right]$ is the coefficient to declare the importance between $R_{j}$ and $R_{j} \left( {X_{i} } \right)$.

Step 2: Establish the global weak ranking (WR).

Based on Eq. (15), compute the global weak ranking $R\left( {X_{ij} } \right)$.

Step 3: Compute the weak ranking of $X_{i} \left( {i = 1,2, \cdots ,m} \right)$.

16 $$ \tilde{R}\left( {X_{i} } \right) = \sum\limits_{j = 1}^{n} {R\left( {X_{ij} } \right)} . $$

Step 4: Obtain the preference intensity (PI).

The average PI of $X_{i}$ over $X_{\kappa }$ can be defined as:

17 $$ P\left( {X_{i} ,X_{\kappa } } \right) = \frac{{\sum\nolimits_{j = 1}^{n} {\max \left[ {R\left( {X_{\kappa j} } \right) - R\left( {X_{ij} } \right),0} \right]} }}{{\left( {m - 1} \right)n^{2} }}. $$

The net PI of $X_{i}$ over $X_{\kappa }$ can be defined as:

18 $$ \Delta P\left( {X_{i} ,X_{\kappa } } \right) = P\left( {X_{i} ,X_{\kappa } } \right) - P\left( {X_{\kappa } ,X_{i} } \right). $$

Step 5: Construct the preference/indifference/incomparability (PIR) structure.

1. If $$\left| {P\left( {X_{i} ,X_{\kappa } } \right)} \right| \le \mu$$,

19 $$ {\text{Then}}\,\left\{ {\begin{array}{*{20}c} {X_{i} \, I \, X_{\kappa } ,{\text{ if }}\left| {P\left( {X_{i} ,X_{\kappa } } \right)} \right| \le \theta {\text{ and }}\left| {P\left( {X_{\kappa } ,X_{i} } \right)} \right| \le \theta \, } \\ {X_{i} \, R \, X_{\kappa } ,{\text{ if }}\left| {P\left( {X_{i} ,X_{\kappa } } \right)} \right| > \theta {\text{ and }}\left| {P\left( {X_{\kappa } ,X_{i} } \right)} \right| > \theta } \\ \end{array} } \right. $$

(2) If $$\left| {P\left( {X_{i} ,X_{\kappa } } \right)} \right| > \mu$$,
  20 $$ {\text{Then}}\,\left\{ {\begin{array}{*{20}c} {X_{i} \, P \, X_{\kappa } ,{\text{ if }}\frac{{\min \left( {P\left( {X_{i} ,X_{\kappa } } \right),P\left( {X_{\kappa } ,X_{i} } \right)} \right)}}{{\left| {\Delta P\left( {X_{i} ,X_{\kappa } } \right)} \right|}} < \vartheta {\text{ and }}P\left( {X_{i} ,X_{\kappa } } \right) > P\left( {X_{\kappa } ,X_{i} } \right)} \\ {X_{\kappa } \, P \, X_{i} ,{\text{ if }}\frac{{\min \left( {P\left( {X_{i} ,X_{\kappa } } \right),P\left( {X_{\kappa } ,X_{i} } \right)} \right)}}{{\left| {\Delta P\left( {X_{i} ,X_{\kappa } } \right)} \right|}} < \vartheta {\text{ and }}P\left( {X_{i} ,X_{\kappa } } \right) < P\left( {X_{\kappa } ,X_{i} } \right)} \\ {X_{i} \, R \, X_{\kappa } ,{\text{ if }}\frac{{\min \left( {P\left( {X_{i} ,X_{\kappa } } \right),P\left( {X_{\kappa } ,X_{i} } \right)} \right)}}{{\left| {\Delta P\left( {X_{i} ,X_{\kappa } } \right)} \right|}} \ge \vartheta \, } \\ \end{array} } \right., $$

where $\mu$, $\theta$, $\vartheta$ are three predefined thresholds [53].

Step 6: Determine the strong ranking based on the weak ranking and PIR.

## Assessment of RER under the stress of COVID-19 using the new IT2F-ORESTE method based on distance and likelihood

### Construct the indicator system for RER under the stress of COVID-19 by Delphi method

In this section, the Delphi approach is applied to construct a comprehensive indicator system for RER based on the interview with 35 magisterial and accomplished experts (20 experts from the regional economic field, 10 experts from government management field, 5 experts from medical care and public health field). These experts are invited to conduct questionnaire surveys on the indicators. The Delphi method are applied to construct the indicator system for RER under the stress of COVID-19. The questionnaire surveys with the above 35 experts are directed in following three rounds:

In the first round, the adaptability of the indicators (0 is no and 1 is yes) are appraised by experts. Then, the indicators with a score below 0.50 are removed, which contains Location advantages ($\tilde{C}_{2}$), Scientific and technological innovation talent resources allocation efficiency ($\tilde{C}_{12}$), Per capita fiscal expenditure ($\tilde{C}_{16}$).

In the second round, the Likert 5 scale are applied to evaluated the relative significance of every indicator (on a scale of 1, 3, 5, 7, and 9, respectively, which displays it is greatly unimportant, a little unimportant, medium, a little important, greatly important). The indicators with final score below 5.5 are removed. That is, the indicators deleted in the second round include Internet economy development environment ($\tilde{C}_{10}$), Small and medium-sized enterprises develop vitality ($\tilde{C}_{11}$), Scientific and technological innovation talent resources allocation efficiency ($\tilde{C}_{12}$), Optimization of financial structure ($\tilde{C}_{17}$), Government financial self-sufficient capacity ($\tilde{C}_{23}$). By means of inquiring experts, the remaining secondary indicator of Labor forces ($\tilde{T}_{3}$) is incorporated into Government's ability ($\tilde{T}_{5}$). Then, an adjusted indicator system is acquired.

In the third round, experts have no objection to the constructed indicator system.

**Fig. 2:**
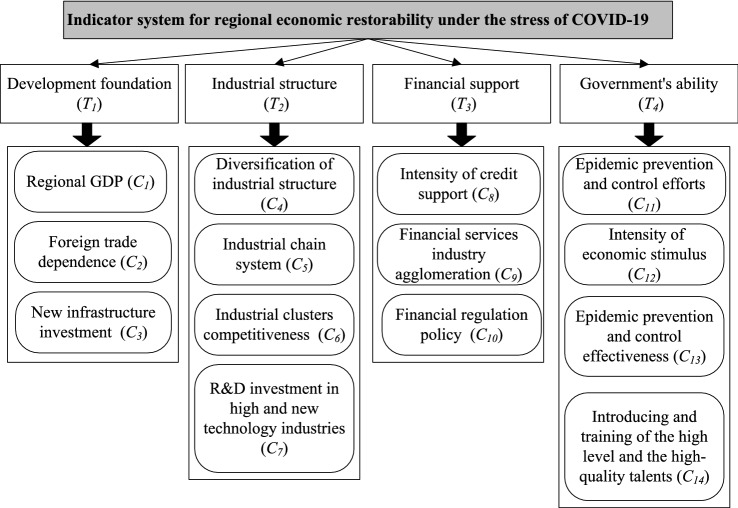
Indicator system for RER under the stress of COVID-19

Through three rounds of survey, the indicator system, which includes 4 first-level indicators and 15 s-level indicators, are finally obtained, shown in Fig. 2.

### The proposed IT2F-ORESTE method

This section develops the IT2F-ORESTE method based on distance measure and likelihood to deal with the assessment problem of RER under COVID-19 epidemic stress in which the preference values of alternatives are denoted by IT2FSs or crisp numbers and the weights of criteria are unknown.

The basic notations are shown in Table 2.

**Table 2:** Parameters and its meaning

Parameter	Meaning
$X_{i}$	The $i$ th alterative (region)
$C_{j}$	The $j$ th criterion (assessment indicators of RER)
$\omega_{j}$	The weight of $j$ th criterion (assessment indicators of RER)
$E_{l}$	The $l$ th expert
$\delta$	The number of quantitative indicators
$\overline{D}_{l}$	The initial decision matrix
$b_{ij}$	The converted linguistic indicator value
$L_{ij}$	The linguistic indicator value given by experts
$\tilde{\tilde{A}}_{ij}$	The corresponding IT2FSs
$\tilde{A}_{ij}^{\omega }$	The preference degree of $C_{j}$ for $C_{i}$
$\tilde{G}\left( {A_{ij} } \right)$	Global preference scores of $X_{i}$ with respect to the $C_{j}$
$\tilde{\tilde{R}}\left( {X_{i} } \right)$	The average preference degree of $X_{i}$
$\tilde{P}_{j} \left( {X_{i} ,X_{\kappa } } \right)$	The preference intensity $X_{i}$ over $X_{\kappa }$ with respect to $C_{j}$
$\tilde{P}\left( {X_{i} ,X_{\kappa } } \right)$	The average preference intensity $X_{i}$ over $X_{\kappa }$ with respect to $C_{j}$
$\Delta \tilde{P}\left( {X_{i} ,X_{\kappa } } \right)$	The net preference intensity $X_{i}$ over $X_{\kappa }$ with respect to $C_{j}$
$\varepsilon$	The preference threshold
$\lambda$	The indifference threshold
$\gamma$	The preference intensity indifference threshold

Assume that $X = \left\{ {X_{i} \left| {i = 1,2, \cdots ,m} \right.} \right\}$ is a set of alternatives (regionals), $C = \left\{ {C_{j} \left| {j = 1,2, \cdots ,n} \right.} \right\}$ is a set of criteria (assessment indicators of RER), $\omega = \left\{ {\omega_{j} \left| {j = 1,2, \cdots ,n} \right.} \right\}$ is a set of criteria weights and $E = \left\{ {E_{l} \left| {l = 1,2, \cdots ,q} \right.} \right\}$ is a set of experts. Assume that there are $\delta$ ($0 \le \delta \le n$) quantitative indicators and $n - \delta$ qualitative indicators. The proposed IT2F-ORESTE method contains three phases:

#### Phase I: Collect assessment indicator information of RER

In general, indicator system includes both the quantitative indicator (such as per capita GDP, fixed asset investment, total retail sales of consumer goods) and qualitative indicator (such as industrial transformation and upgrading capacity, business environment, financial support), in which the preference values of the quantitative indicator can be dimensionless and the preference values of the qualitative indicator cannot be quantified as crisp number [63].

The quantitative indicator information is obtained by investigation or estimation, while the qualitative indicator information is the experts’ subjective evaluation based on their experience, knowledge or ability. For solving the assessment problem with quantitative and qualitative indicator information in the same environments, next a transformation function that converts quantitative indicator information to the IT2FSs is developed.

##### Definition 8

Let $B = \left\{ {b_{j} \left| {j = 1,2, \cdots ,n} \right.} \right\}$ be a crisp number set. $b^{ - } = \min \left\{ {b_{j} \left| {j = 1,2, \cdots ,n} \right.} \right\}$, $b^{ + } = \max \left\{ {b_{j} \left| {j = 1,2, \cdots ,n} \right.} \right\}$. Then, $b_{j}$ corresponding linguistic terms (LTs) and IT2FSs are denoted as follows (Table 3):

**Table 3:** LTs and their corresponding IT2FSs

Crisp number interval	LTs	LTs	IT2FSs
$\left[ {b^{ - } ,b^{ - } + \frac{{\left( {b^{ + } - b^{ - } } \right)}}{7}} \right)$	Very unimportant (VN)	Extremely weak (EW)	[(0,0,0,0.1;1), (0,0,0,0.05;0.9)]
$\left[ {b^{ - } + \frac{{\left( {b^{ + } - b^{ - } } \right)}}{7},b^{ - } + \frac{{2\left( {b^{ + } - b^{ - } } \right)}}{7}} \right)$	Quite unimportant (QN)	Very weak (VW)	[(0,0.1,0.1,0.3;1), (0.05,0.1,0.1,0.2;0.9)]
$\left[ {b^{ - } + \frac{{2\left( {b^{ + } - b^{ - } } \right)}}{7},b^{ - } + \frac{{3\left( {b^{ + } - b^{ - } } \right)}}{7}} \right)$	Unimportant (U)	Weak (W)	[(0.1,0.3,0.3,0.5;1), (0.2,0.3,0.3,0.4;0.9)]
$\left[ {b^{ - } + \frac{{3\left( {b^{ + } - b^{ - } } \right)}}{7},b^{ - } + \frac{{4\left( {b^{ + } - b^{ - } } \right)}}{7}} \right)$	Medium (M)	Medium (M)	[(0.3,0.5,0.5,0.7;1), (0.4,0.5,0.5,0.6;0.9)]
$\left[ {b^{ - } + \frac{{4\left( {b^{ + } - b^{ - } } \right)}}{7},b^{ - } + \frac{{5\left( {b^{ + } - b^{ - } } \right)}}{7}} \right)$	Important (I)	Strong (S)	[(0.5,0.7,0.7,0.9;1), (0.6,0.7,0.7,0.8;0.9)]
$\left[ {b^{ - } + \frac{{5\left( {b^{ + } - b^{ - } } \right)}}{7},b^{ - } + \frac{{6\left( {b^{ + } - b^{ - } } \right)}}{7}} \right)$	Quite important (QI)	Very strong (VS)	[(0.7,0.9,0.9,1;1), (0.8,0.9,0.9,0.95;0.9)]
$\left[ {b^{ - } + \frac{{6\left( {b^{ + } - b^{ - } } \right)}}{7},b^{ + } } \right]$	Very important (VI)	Extremely strong (ES)	[(0.9,1,1,1;1), (0.95,1,1,1;0.9)]

##### Example 1

In 2020, the GDP of Qindao, Jinan, Yantai, Weifang, Linyi of Shandong province of China are 12,400.56, 10,140.91, 7816.42, 5872.20, 4805.25 (Unit:100 million RMB), respectively. The Crisp number interval are $\left[ {4805.25,5890.25} \right)$, $\left[ {5890.25,6975.25} \right)$, $\left[ {6975.25,8060.25} \right)$, $\left[ {8060.25,9145.25} \right)$, $\big[ 9145.25,10230.25 \big)$, $\left[ {10230.25,11315.25} \right)$, $\left[ {11315.25,12400.56} \right]$, respectively. Then, indicator information of the GDP of Qindao corresponding linguistic terms and IT2FSs are $\left\{ {{\text{Very strong }}\left( {{\text{VS}}} \right)} \right\}$ and [(0.9,1,1,1;1), (0.95,1,1,1;0.9)], respectively. Indicator information of the GDP of Qindao corresponding linguistic terms and IT2FSs are {Extremely Strong (ES)} and [(0.9,1,1,1;1), (0.95,1,1,1;0.9)], respectively, and so on.

Step 1: Convert $\delta$ ($0 \le \delta \le n$) quantitative indicator information obtained by investigation to the LTs based on the definition 8.

Step 2: Establish the initial decision matrix.

The initial decision matrix $\overline{D}_{l}$ including LTs converted by definition 8 and LTs given by expert $E_{l}$ is established as follows:

21 $$ \overline{D}_{l} = \left[ {\begin{array}{*{20}c} {b_{11} } & {b_{12} } & \cdots & {b_{1\delta } } & {L_{1,\delta + 1} } & \cdots & {L_{1,n} } \\ {b_{21} } & {b_{22} } & \cdots & {b_{2\delta } } & {L_{2,\delta + 1} } & \cdots & {L_{2,n} } \\ \vdots & \vdots & \ddots & \vdots & \vdots & \ddots & \vdots \\ {b_{m1} } & {b_{m2} } & \cdots & {b_{m\delta } } & {L_{m,\delta + 1} } & \cdots & {L_{m,n} } \\ \end{array} } \right], $$

where $b_{ij}$ ($i = = 1,2, \cdots m,j = 1,2, \cdots ,\delta$) denotes the converted linguistic indicator value, and $L_{ij}$ ($i = = 1,2, \cdots m,j = \delta + 1,\delta + 2, \cdots ,n$) denotes the linguistic indicator value given by experts.

Step 3: Normalize the linguistic decision matrix $\overline{D}_{l}$.

**Fig. 3:**
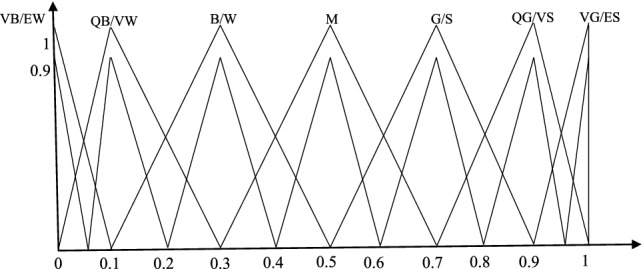
The MF of IT2FSs for LTs

In general, the decision matrix $\overline{D}_{l}$ should be normalized before solving the real assessment problems, except that all the assessment indicators have the same form. In this step, based on Table 4 and Fig. 3, the decision matrix $\overline{D}_{l}$ is normalized by utilizing the following equation:

22 $$ L_{ij} = \left\{ {\begin{array}{*{20}c} { \, L_{ij} {\text{ for benefit indicator}}} \\ {\left( {L_{ij} } \right)^{c} {\text{ for cost indicator}}} \\ \end{array} } \right.. $$

**Table 4:** The complementary relations

LT	VN/EW	QN/VW	N/W	M	I/S	QI/VS	VI/ES
(LT)^C^	VI/ES	QI/VS	I/S	M	N/W	NB/VW	VN/EW

Step 4: Convert the normalized LTs into the corresponding IT2FSs, which can be represented by:

23 $$ \tilde{\tilde{A}} = \left[ {\begin{array}{*{20}c} {\tilde{\tilde{A}}_{11} } & {\tilde{\tilde{A}}_{12} } & \cdots & {\tilde{\tilde{A}}_{1\delta } } & {\tilde{\tilde{A}}_{1,\delta + 1} } & \cdots & {\tilde{\tilde{A}}_{1,n} } \\ {\tilde{\tilde{A}}_{21} } & {\tilde{\tilde{A}}_{22} } & \cdots & {\tilde{\tilde{A}}_{2\delta } } & {\tilde{\tilde{A}}_{2,\delta + 1} } & \cdots & {\tilde{\tilde{A}}_{2,n} } \\ \vdots & \vdots & \ddots & \vdots & \vdots & \ddots & \vdots \\ {\tilde{\tilde{A}}_{m1} } & {\tilde{\tilde{A}}_{m2} } & \cdots & {\tilde{\tilde{A}}_{m\delta } } & {\tilde{\tilde{A}}_{m,\delta + 1} } & \cdots & {\tilde{\tilde{A}}_{m,n} } \\ \end{array} } \right], $$

where $\tilde{\tilde{A}}_{ij}$ ($i = 1,2, \cdots m,j = 1,2, \cdots ,n$) denotes the corresponding IT2FSs.

Step 5: Aggregate the converted IT2FSs by the weighted average (WA) operator. If the weights of the experts are not given, a general solution is that $$w_{l} = {\raise0.7ex\hbox{$1$} \!\mathord{\left/ {\vphantom {1 q}}\right.\kern-\nulldelimiterspace} \!\lower0.7ex\hbox{$q$}}$$, $$\left( {l = 1,2, \cdots ,q} \right)$$, then the IT2FS-WA operator can be defined as:

24 $$ \begin{gathered} \tilde{A}_{ij} = IT2FS - WA\left( {\left( {\tilde{\tilde{A}}_{ij} } \right)^{1} ,\left( {\tilde{\tilde{A}}_{ij} } \right)^{2} , \cdots ,\left( {\tilde{\tilde{A}}_{ij} } \right)^{q} } \right) = \sum\nolimits_{l = 1}^{q} {w_{l} \left( {\tilde{\tilde{A}}_{ij} } \right)^{l} } \hfill \\ = \left[ \begin{gathered} \left( {\sum\nolimits_{l = 1}^{q} {w_{l} a_{l1}^{L} } ,\sum\nolimits_{l = 1}^{q} {w_{l} a_{l2}^{L} } ,\sum\nolimits_{l = 1}^{q} {w_{l} a_{l3}^{L} } ,\sum\nolimits_{l = 1}^{q} {w_{l} a_{l4}^{L} } ;\mathop {\min }\limits_{l = 1,2, \cdots ,q} \left( {h_{{\tilde{\tilde{A}}_{l} }}^{L} } \right)} \right), \hfill \\ \left( {\sum\nolimits_{l = 1}^{q} {w_{l} a_{l1}^{U} } ,\sum\nolimits_{l = 1}^{q} {w_{l} a_{l2}^{U} } ,\sum\nolimits_{l = 1}^{q} {w_{l} a_{l3}^{U} } ,\sum\nolimits_{l = 1}^{q} {w_{l} a_{l4}^{U} } ;\mathop {\min }\limits_{l = 1,2, \cdots ,q} \left( {h_{{\tilde{\tilde{A}}_{l} }}^{U} } \right)} \right) \hfill \\ \end{gathered} \right]. \hfill \\ \end{gathered} $$

#### Phase II: Determinate the weight of indicator

The weights of indicators can have a significant impact on the assessment results. Nevertheless, it is hard to denote accurately the weights of indicators by using crisp number or LTs in the complex environments. In contrast, experts can make pairwise comparisons among indicators. In a more ideal situation, the preference degree (PD) between two indicators can be accurately measured by LTs. Therefore, in this paper, the preference relations (PRs) based on the IT2FSs are constructed to obtain the weights of indicators.

Step 6: Establish the PRs matrix with LTs.

Experts are invited to give the PDs between two indicators by LTs. Next, their LTs are converted into IT2FSs. Then, the converted IT2FSs are aggregated by WA operator. That is, the PRs matrix with IT2FSs is established as:

25 $$ \tilde{A}^{\omega } = \left[ {\begin{array}{*{20}c} {\tilde{A}_{11}^{\omega } } & {\tilde{A}_{12}^{\omega } } & \cdots & {\tilde{A}_{1n}^{\omega } } \\ {\tilde{A}_{21}^{\omega } } & {\tilde{A}_{22}^{\omega } } & \cdots & {\tilde{A}_{2n}^{\omega } } \\ \vdots & \vdots & \ddots & \vdots \\ {\tilde{A}_{n1}^{\omega } } & {\tilde{A}_{n2}^{\omega } } & \cdots & {\tilde{A}_{nn}^{\omega } } \\ \end{array} } \right], $$

where $\tilde{A}_{ij}^{\omega }$ ($i  = 1,2, \cdots n,j = 1,2, \cdots ,n$) denotes the corresponding IT2FSs and represents the PD of indicator $C_{j} \left( {j = 1,2, \cdots ,n} \right)$ for $C_{i} \left( {i = 1,2, \cdots ,n} \right)$. In particular, $\tilde{A}_{11}^{\omega } = \tilde{A}_{22}^{\omega } = \cdots = \tilde{A}_{nn}^{\omega } = \left[ {\left( {1,1,1,1;1} \right),\left( {1,1,1,1;0.9} \right)} \right]$.

Step 7: Compute the PDs of one indicator over the others.

The PD of indicator $C_{j} \left( {j = 1,2, \cdots ,n} \right)$ over the others can be computed by collecting the all elements (except $C_{jj}$) in the $i$ th row of matrix $\tilde{A}^{\omega }$ based on the IT2FPA operator (Eq. (26)).

26 $$ \begin{gathered} \tilde{A}_{j}^{\omega } = IT2FPA\left( {\tilde{A}_{j1}^{\omega } ,\tilde{A}_{j2}^{\omega } , \cdots ,\tilde{A}_{jn}^{\omega } } \right) \hfill \\ = \left[ \begin{gathered} \left( {\frac{{\sum\nolimits_{\xi = 1}^{n} {\left( {1 + T\left( {\tilde{A}_{j\xi }^{\omega } } \right)} \right)\alpha_{\xi 1}^{L} } }}{{\sum\nolimits_{\xi = 1}^{n} {\left( {1 + T\left( {\tilde{A}_{j\xi } } \right)} \right)} }},\frac{{\sum\nolimits_{\xi = 1}^{n} {\left( {1 + T\left( {\tilde{A}_{j\xi }^{\omega } } \right)} \right)\alpha_{\xi 2}^{L} } }}{{\sum\nolimits_{\xi = 1}^{n} {\left( {1 + T\left( {\tilde{A}_{j\xi } } \right)} \right)} }},\frac{{\sum\nolimits_{\xi = 1}^{n} {\left( {1 + T\left( {\tilde{A}_{j\xi }^{\omega } } \right)} \right)\alpha_{\xi 3}^{L} } }}{{\sum\nolimits_{\xi = 1}^{n} {\left( {1 + T\left( {\tilde{A}_{j\xi } } \right)} \right)} }},\frac{{\sum\nolimits_{\xi = 1}^{n} {\left( {1 + T\left( {\tilde{A}_{j\xi }^{\omega } } \right)} \right)\alpha_{\xi 4}^{L} } }}{{\sum\nolimits_{\xi = 1}^{n} {\left( {1 + T\left( {\tilde{A}_{j\xi } } \right)} \right)} }};\mathop {\min }\limits_{\xi = 1,2, \cdots ,n} \left( {h_{{\tilde{A}_{j\xi }^{\omega } }}^{L} } \right)} \right), \hfill \\ \left( {\frac{{\sum\nolimits_{\xi = 1}^{n} {\left( {1 + T\left( {\tilde{A}_{j\xi }^{\omega } } \right)} \right)\alpha_{\xi 1}^{U} } }}{{\sum\nolimits_{\xi = 1}^{n} {\left( {1 + T\left( {\tilde{A}_{j\xi }^{\omega } } \right)} \right)} }},\frac{{\sum\nolimits_{\xi = 1}^{n} {\left( {1 + T\left( {\tilde{A}_{j\xi }^{\omega } } \right)} \right)\alpha_{\xi 2}^{U} } }}{{\sum\nolimits_{\xi = 1}^{n} {\left( {1 + T\left( {\tilde{A}_{j\xi }^{\omega } } \right)} \right)} }},\frac{{\sum\nolimits_{\xi = 1}^{n} {\left( {1 + T\left( {\tilde{A}_{j\xi }^{\omega } } \right)} \right)\alpha_{\xi 3}^{U} } }}{{\sum\nolimits_{\xi = 1}^{n} {\left( {1 + T\left( {\tilde{A}_{j\xi }^{\omega } } \right)} \right)} }},\frac{{\sum\nolimits_{\xi = 1}^{n} {\left( {1 + T\left( {\tilde{A}_{j\xi }^{\omega } } \right)} \right)\alpha_{\xi 4}^{U} } }}{{\sum\nolimits_{\xi = 1}^{n} {\left( {1 + T\left( {\tilde{A}_{j\xi }^{\omega } } \right)} \right)} }};\mathop {\min }\limits_{\xi = 1,2, \cdots ,n} \left( {h_{{\tilde{A}_{j\xi }^{\omega } }}^{U} } \right)} \right) \hfill \\ \end{gathered} \right] \hfill \\ where\,T\left( {\tilde{A}_{j\xi }^{\omega } } \right) = \sum\nolimits_{\xi = 1,\xi \ne \psi }^{M} {\left( {1 - d\left( {\tilde{A}_{j\xi }^{\omega } ,\tilde{A}_{j\psi }^{\omega } } \right)} \right)} \hfill \\ \end{gathered} $$

Step 8: Compute the weight of indicator.

Based on the likelihood of two PDs between two indicators $I\left( {\tilde{A}_{i}^{\omega } \ge \tilde{A}_{j}^{\omega } } \right)$, the weight of indicator $C_{i} \left( {i = 1,2, \cdots ,n} \right)$ can be computed as:

27 $$ \omega_{i} = \frac{{I\left( {\tilde{A}_{i}^{\omega } \ge \tilde{A}_{j}^{\omega } } \right)}}{{\sum\nolimits_{i = 1}^{n} {I\left( {\tilde{A}_{i}^{\omega } \ge \tilde{A}_{j}^{\omega } } \right)} }}. $$

#### Phase III: Obtain the ranking result

The traditional ORESTE method denotes the decision-making information only by using general ranking [64]. Nevertheless, the downside of this method is a loss of much valuable information, which may obtain an unreasonable result. In order to further improve the drawbacks of this method, the distance measure based on the extend vertex method are applied to establish GPS function since it encompasses more preference information on the PR between the indicators and the PR between the alternatives than general ranking.

Step 9: Calculate the GPS $\tilde{G}\left( {A_{ij} } \right)$ of alternative $X_{i}$ with respect to the indicator $C_{j}$.

The maximum IT2FS $\tilde{A}_{{_{j} }}^{ + }$ of $X_{i}$ with respect to the $C_{j}$ are defined as follows:

28 $$ \tilde{A}_{{_{j} }}^{ + } = \left\{ {\begin{array}{*{20}c} {\mathop {\max }\limits_{i = 1,2, \cdots ,m} \left\{ {\tilde{A}_{ij} } \right\},{\text{ for the benefit indicator}}} \\ {\mathop {\min }\limits_{i = 1,2, \cdots ,m} \left\{ {\tilde{A}_{ij} } \right\},{\text{ for the cost indicator}}} \\ \end{array} } \right. $$

The weight of the most significant indicator $C_{j}$ are defined as follows:

29 $$ \omega^{ + } = \mathop {\max }\limits_{j = 1,2, \cdots ,n} \left\{ {\omega_{j} } \right\} = \mathop {\max }\limits_{j = 1,2, \cdots ,n} \left\{ {\tilde{A}_{j}^{\omega } } \right\} $$

Based on the extend vertex method, let the distance measure $d\left( {\tilde{A}_{ij} ,\tilde{A}_{{_{j} }}^{ + } } \right)$ replace the $R_{j} \left( {X_{i} } \right)$ and let the distance measure $d\left( {\omega_{j} ,\omega^{ + } } \right)$ replace $R_{j}$.

Then, the GPS $\tilde{G}\left( {X_{ij} } \right)$ can be calculated as follows:

30 $$ \tilde{G}\left( {X_{ij} } \right) = \sqrt {\rho \left( {d\left( {\tilde{A}_{ij} ,\tilde{A}_{{_{j} }}^{ + } } \right)} \right)^{2} + \left( {1 - \rho } \right)\left( {d\left( {\omega_{j} ,\omega^{ + } } \right)} \right)^{2} } , $$

where $\rho \in \left[ {0,1} \right]$ is the coefficient to declare the importance between $d\left( {\tilde{A}_{ij} ,\tilde{A}_{{_{j} }}^{ + } } \right)$ and $d\left( {\omega_{j} ,\omega^{ + } } \right)$. Obviously, the smaller $\tilde{G}\left( {X_{ij} } \right)$ is, the closer $\tilde{A}_{ij}$ is to $\tilde{A}_{{_{j} }}^{ + }$ and the better $\tilde{A}_{ij}$ should be.

Step 10: Establish the global WR.

The average PD of the alternative $X_{i}$ can be defined as follows:

31 $$ \tilde{\tilde{R}}\left( {X_{i} } \right) = \frac{1}{n}\sum\nolimits_{j = 1}^{n} {\tilde{G}\left( {X_{ij} } \right)} $$

Then, the WR can be obtained as follows:

If $$\tilde{\tilde{R}}\left( {X_{i} } \right) - \tilde{\tilde{R}}\left( {X_{\kappa } } \right) < 0$$, $$X_{i} \, P \, X_{\kappa }$$;

If $$\tilde{\tilde{R}}\left( {X_{i} } \right) - \tilde{\tilde{R}}\left( {X_{\kappa } } \right) = 0$$, $$X_{i} \, I \, X_{\kappa }$$.

Step 11: Construct the PIR structure of alternatives $X_{i} \left( {i = 1,2, \cdots ,m} \right)$.

1. Calculate the PIs.

The PI of $X_{i}$ over $X_{\kappa }$ with respect to $C_{j}$ can be defined as follows:

32 $$ \tilde{P}_{j} \left( {X_{i} ,X_{\kappa } } \right) = \max \left[ {\tilde{\tilde{R}}\left( {X_{\kappa j} } \right) - \tilde{\tilde{R}}\left( {X_{ij} } \right),0} \right] $$

The average PI of $X_{i}$ over $X_{\kappa }$ with respect to $C_{j}$ can be defined as:

33 $$ \tilde{P}\left( {X_{i} ,X_{\kappa } } \right) = \frac{{\sum\nolimits_{j = 1}^{n} {\max \left[ {\tilde{\tilde{R}}\left( {X_{\kappa j} } \right) - \tilde{\tilde{R}}\left( {X_{ij} } \right),0} \right]} }}{n}. $$

The net PI of $X_{i}$ over $X_{\kappa }$ can be defined as:

34 $$ \Delta \tilde{P}\left( {X_{i} ,X_{\kappa } } \right) = \tilde{P}\left( {X_{i} ,X_{\kappa } } \right) - \tilde{P}\left( {X_{\kappa } ,X_{i} } \right). $$

(2) Determine the preference threshold (PT) and the indifference threshold (IT).

The PT $\varepsilon$ can be defined as follows:

35 $$ \varepsilon = \frac{\gamma }{n}. $$

The IT $\lambda$ can be defined as follows:

36 $$ \left\{ {\begin{array}{*{20}c} {\lambda = \frac{{\left( {n + 2} \right)\gamma }}{2n},{\text{ if }}n{\text{ is odd}}} \\ {\lambda = \frac{\gamma }{2},{\text{ if }}n{\text{ is even}}} \\ \end{array} } \right., $$

where $\gamma$ is PI indifference threshold that $\gamma = \sqrt \rho * \left( {{\upsilon \mathord{\left/ {\vphantom {\upsilon 6}} \right. \kern-\nulldelimiterspace} 6}} \right)$ with $\upsilon$ being the minimal difference between LTs that sustain the indifference relation. The quantitative value of $\upsilon$ can be obtained on the basis of the real circumstances.

(3) Construct the PIR structure.

Based on the PT $\varepsilon$ and IT $\lambda$, the PIR structure is determined as follows:

37 $$ \left\{ {\begin{array}{*{20}l} {X_{i} \, P \, X_{\kappa } ,{\text{ if }}\left| {\Delta \tilde{P}\left( {X_{i} ,X_{\kappa } } \right)} \right| \ge \varepsilon {\text{ and }}\Delta \tilde{P}\left( {X_{i} ,X_{\kappa } } \right) > 0} \\ {X_{\kappa } \, P \, X_{i} ,{\text{ if }}\left| {\Delta \tilde{P}\left( {X_{i} ,X_{\kappa } } \right)} \right| \ge \varepsilon {\text{ and }}\Delta \tilde{P}\left( {X_{i} ,X_{\kappa } } \right) \le 0} \\ {X_{i} \, I \, X_{\kappa } {,}\,{\text{if }}\left| {\Delta \tilde{P}\left( {X_{i} ,X_{\kappa } } \right)} \right| < \varepsilon , \, \tilde{P}\left( {X_{i} ,X_{\kappa } } \right) \!<\! \lambda {\text{ and }}\tilde{P}\left( {X_{\kappa } ,X_{i} } \right) \!<\! \lambda \, } \\ {X_{i} \, R \, X_{\kappa } ,\,{\text{if }}\left| {\Delta \tilde{P}\left( {X_{i} ,X_{\kappa } } \right)} \right| < \varepsilon ,\tilde{P}\left( {X_{i} ,X_{\kappa } } \right) < \lambda {\text{ or }}\tilde{P}\left( {X_{\kappa } ,X_{i} } \right) < \lambda \, } \\ \end{array} } \right.. $$

Step 12: Obtain the strong ranking based on the WR and the PIR structure.

**Fig. 4:**
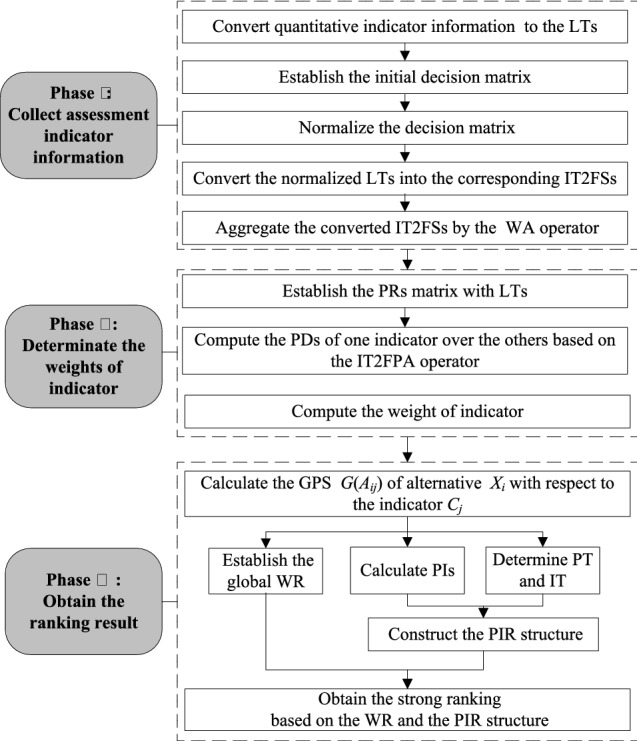
The flowchart of the developed new IT2F-ORESTE method

The developed IT2F-ORESTE method is an improved assessment method of RER under COVID-19 epidemic stress in which the preference values of alternatives are denoted by IT2FSs or crisp numbers and the weights of criteria are represented by IT2FSs. Compared with the forthcoming assessment method, the developed IT2F-ORESTE method can handle the indicator weights that are denoted as IT2FSs, which can to a great extent information loss in time of converting fuzzy weights into crisp number weights. What’s more, PIR structure is applied to distinguish the specific relationships between alternatives. Surprisingly, the incomparable relation that forthcoming assessment method are neglected is taken into consideration. For understanding the developed IT2F-ORESTE method better, the flowchart of this method is shown in Fig. 4.

## A case study: the assessment of RER of cities under the stress of COVID-19 epidemic

In this section, the developed IT2F-ORESTE method with distance measure and likelihood is applied to assess the RER of cities under the stress of COVID-19 epidemic.

### Case description

The COVID-19, as an on-going global pandemic continually spreading across the world, has led to a truly worldwide crisis. An increasing number of scholars and government officials have begun to put more emphasis on the geographically uneven impact and consequences of this pandemic. Different regions, in particular, are definitely discovered to possess a wide variation with regard to the efficacy of region policy/measure to contain it, and subsequent socio-economic consequences. Traditional regional structural advantages might lose advantages for economic resilience under the stress of COVID-19 epidemic. As an example, evidence has revealed that cities with dense market clustering and workforce base, or with wider global interconnections in supply chain have exhibited higher economic vulnerability.

Suppose RER of five cities, including $X_{1}$, $X_{2}$, $X_{3}$, $X_{4}$, $X_{5}$, are assessed under COVID-19 epidemic stress. In the indicator system shown as Fig. 2, the indicator $C_{1}$ is quantitative indicator with known data while the others are qualitative ones with unknown data. The weights of these indicators are unknown. Five experts ($E_{1}$, $E_{2}$ and $E_{3}$ from the regional economic field, $E_{4}$ from government management field, $E_{5}$ from medical care and public health field) are invited to give the initial preference value of indicator. The PDs between two indicators are represented by LTs. $\omega = \left\{ {\omega_{j} \left| {j = 1,2, \cdots ,n} \right.} \right\}$ is a set of criteria weights. Let L1 = {Extremely weak (EW), Very weak (VW), Weak (W), Medium (M), Strong (S), Very strong (VS), Extremely strong (ES)} be a LTs for assessing the qualitative indicators. Let L2 = {Very unimportant (VN), Quite unimportant (QN), Unimportant (U), Medium (M), Important (I), Quite important (QI), Very important (VI)} be a LTs for assessing the PDs between two indicators.

### Solving the case by the developed IT2F-ORESTE method

#### Phase I: Collect assessment indicator information of RER

Step 1: Convert quantitative indicator information obtained by investigation to the LTs based on the definition 8.

Quantitative indicator values are from the corresponding city statistical yearbook of 2020. The regional GDP ($C_{1}$) of each city can be converted into the LTs, shown in Table 5 ($C_{1}$ is the benefit indicator).

**Table 5:** Convert the values of GDP into the LTs

City	$X_{1}$	$X_{2}$	$X_{3}$	$X_{4}$	$X_{5}$
$C_{1}$	12,400.56	10,140.91	7816.42	5872.20	4805.25
LTs	ES	S	W	EW	EW
IT2FSs	[(0.9,1,1,1;1), (0.95,1,1,1;0.9)]	[(0.5,0.7,0.7,0.9;1), (0.6,0.7,0.7,0.8;0.9)]	[(0.1,0.3,0.3,0.5;1), (0.2,0.3,0.3,0.4;0.9)]	[(0,0,0,0.1;1), (0,0,0,0.05;0.9)]	[(0,0,0,0.1;1), (0,0,0,0.05;0.9)]

Unit ($C_{1}$):100 million RMB

Step 2: Establish the initial linguistic decision matrix.

The evaluations on the cities over the 14 indicators given by the 5 experts are shown in Tables 25, 26, 27, 28, 29 (see the Appendix).

Step 3: Normalize the decision matrix $\overline{D}_{l}$.

In this decision matrixes, each indicator corresponds to benefit type, and thus, it is not necessary to perform the normalization.

Step 4: Convert the normalized LTs into the corresponding IT2FSs.

Step 5: Aggregate the converted IT2FSs by the WA operator. In this case, the weights of the experts are not given, it is supposed that $$w_{l} = {\raise0.7ex\hbox{$1$} \!\mathord{\left/ {\vphantom {1 5}}\right.\kern-\nulldelimiterspace} \!\lower0.7ex\hbox{$5$}}$$, $$\left( {l = 1,2, \cdots ,5} \right)$$, then:

$$\begin{aligned}\tilde{A}_{11} &= \left[ {\left( {0.90,1.00,1.00,1.00;1} \right),}\right.\\ &\left.{\left( {0.95,1.00,1.00,1.00;0.9} \right)} \right]\end{aligned}$$;

$$\begin{aligned}\tilde{A}_{12} &= \left[ {\left( {0.78,0.94,0.94,1.00;1} \right),}\right.\\ &\left.{\left( {0.86,0.94,0.94,0.91;0.9} \right)} \right]\end{aligned}$$.

$$\begin{aligned}\tilde{A}_{13} &= \left[ {\left( {0.34,0.54,0.54,0.74;1} \right),}\right.\\ &\left.{\left( {0.44,0.54,0.54,0.64;0.9} \right)} \right]\end{aligned}$$;

$$\begin{aligned}\tilde{A}_{14} &= \left[ {\left( {0.70,0.88,0.88,0.98;1} \right),}\right.\\ &\left.{\left( {0.79,0.88,0.88,0.93;0.9} \right)} \right]\end{aligned}$$.

$$\begin{aligned}\tilde{A}_{15} &= \left[ {\left( {0.62,0.82,0.82,0.94;1} \right),}\right.\\ &\left.{\left( {0.72,0.82,0.82,0.88;0.9} \right)} \right]\end{aligned}$$;

$$\begin{aligned}\tilde{A}_{16} &= \left[ {\left( {0.74,0.90,0.90,0.98;1} \right),}\right.\\ &\left.{\left( {0.82,0.90,0.90,0.94;0.9} \right)} \right]\end{aligned}$$.

$$\begin{aligned}\tilde{A}_{17} &= \left[ {\left( {0.46,0.66,0.66,0.84;1} \right),}\right.\\ &\left.{\left( {0.56,0.66,0.66,0.75;0.9} \right)} \right]\end{aligned}$$;

$$\begin{aligned}\tilde{A}_{18} &= \left[ {\left( {0.50,0.70,0.70,0.88;1} \right),}\right.\\ &\left.{\left( {0.60,0.70,0.70,0.79;0.9} \right)} \right]\end{aligned}$$.

$$\begin{aligned}\tilde{A}_{19} &= \left[ {\left( {0.62,0.82,0.82,0.96;1} \right),}\right.\\ &\left.{\left( {0.72,0.82,0.82,0.89;0.9} \right)} \right]\end{aligned}$$;

$$\begin{aligned}\tilde{A}_{110} &= \left[ {\left( {0.54,0.74,0.74,0.88;1} \right),}\right.\\ &\left.{\left( {0.64,0.74,0.74,0.81;0.9} \right)} \right]\end{aligned}$$.

$$\begin{aligned}\tilde{A}_{111} &= \left[ {\left( {0.18,0.38,0.38,0.58;1} \right),}\right.\\ &\left.{\left( {0.28,0.38,0.38,0.48;0.9} \right)} \right]\end{aligned}$$;

$$\begin{aligned}\tilde{A}_{112} &= \left[ {\left( {0.38,0.58,0.58,0.78;1} \right),}\right.\\ &\left.{\left( {0.48,0.58,0.58,0.68;0.9} \right)} \right]\end{aligned}$$.

$$\begin{aligned}\tilde{A}_{113} &= \left[ {\left( {0.22,0.42,0.42,0.62;1} \right),}\right.\\ &\left.{\left( {0.32,0.42,0.42,0.52;0.9} \right)} \right]\end{aligned}$$;

$$\begin{aligned}\tilde{A}_{114} &= \left[ {\left( {0.62,0.80,0.80,0.94;1} \right),}\right.\\ &\left.{\left( {0.71,0.80,0.80,0.87;0.9} \right)} \right]\end{aligned}$$.

$$\begin{aligned}\tilde{A}_{21} &= \left[ {\left( {0.50,0.70,0.70,0.90;1} \right),}\right.\\ &\left.{\left( {0.60,0.70,0.70,0.80;0.9} \right)} \right]\end{aligned}$$;

$$\begin{aligned}\tilde{A}_{22} &= \left[ {\left( {0.38,0.58,0.58,0.78;1} \right),}\right.\\ &\left.{\left( {0.48,0.58,0.58,0.68;0.9} \right)} \right]\end{aligned}$$.

$$\begin{aligned}\tilde{A}_{23} &= \left[ {\left( {0.42,0.62,0.62,0.82;1} \right),}\right.\\ &\left.{\left( {0.52,0.62,0.62,0.72;0.9} \right)} \right]\end{aligned}$$;

$$\begin{aligned}\tilde{A}_{24} &= \left[ {\left( {0.46,0.66,0.66,0.84;1} \right),}\right.\\ &\left.{\left( {0.56,0.66,0.66,0.75;0.9} \right)} \right]\end{aligned}$$.

$$\begin{aligned}\tilde{A}_{25} &= \left[ {\left( {0.38,0.58,0.58,0.78;1} \right),}\right.\\ &\left.{\left( {0.48,0.58,0.58,0.68;0.9} \right)} \right]\end{aligned}$$;

$$\begin{aligned}\tilde{A}_{26} &= \left[ {\left( {0.38,0.58,0.58,0.78;1} \right),}\right.\\ &\left.{\left( {0.48,0.58,0.58,0.68;0.9} \right)} \right]\end{aligned}$$.

$$\begin{aligned}\tilde{A}_{27} &= \left[ {\left( {0.62,0.82,0.82,0.96;1} \right),}\right.\\ &\left.{\left( {0.72,0.82,0.82,0.89;0.9} \right)} \right]\end{aligned}$$;

$$\begin{aligned}\tilde{A}_{28} &= \left[ {\left( {0.58,0.78,0.78,0.94;1} \right),}\right.\\ &\left.{\left( {0.68,0.78,0.78,0.86;0.9} \right)} \right]\end{aligned}$$.

$$\begin{aligned}\tilde{A}_{29} &= \left[ {\left( {0.50,0.70,0.70,0.88;1} \right),}\right.\\ &\left.{\left( {0.60,0.70,0.70,0.79;0.9} \right)} \right]\end{aligned}$$;

$$\begin{aligned}\tilde{A}_{210} &= \left[ {\left( {0.62,0.82,0.82,0.94;1} \right),}\right.\\ &\left.{\left( {0.72,0.82,0.82,0.88;0.9} \right)} \right]\end{aligned}$$.

$$\begin{aligned}\tilde{A}_{211} &= \left[ {\left( {0.34,0.54,0.54,0.74;1} \right),}\right.\\ &\left.{\left( {0.44,0.54,0.54,0.64;0.9} \right)} \right]\end{aligned}$$;

$$\begin{aligned}\tilde{A}_{212} &= \left[ {\left( {0.50,0.70,0.70,0.90;1} \right),}\right.\\ &\left.{\left( {0.60,0.70,0.70,0.80;0.9} \right)} \right]\end{aligned}$$.

$$\begin{aligned}\tilde{A}_{213} &= \left[ {\left( {0.42,0.62,0.62,0.82;1} \right),}\right.\\ &\left.{\left( {0.52,0.62,0.62,0.72;0.9} \right)} \right]\end{aligned}$$;

$$\begin{aligned}\tilde{A}_{214} &= \left[ {\left( {0.58,0.78,0.78,0.92;1} \right),}\right.\\ &\left.{\left( {0.68,0.78,0.78,0.85;0.9} \right)} \right]\end{aligned}$$.

$$\begin{aligned}\tilde{A}_{31} &= \left[ {\left( {0.10,0.30,0.30,0.50;1} \right),}\right.\\ &\left.{\left( {0.20,0.30,0.30,0.40;0.9} \right)} \right]\end{aligned}$$;

$$\begin{aligned}\tilde{A}_{32} &= \left[ {\left( {0.58,0.78,0.78,0.94;1} \right),}\right.\\ &\left.{\left( {0.68,0.78,0.78,0.86;0.9} \right)} \right]\end{aligned}$$.

$$\begin{aligned}\tilde{A}_{33} &= \left[ {\left( {0.18,0.38,0.38,0.58;1} \right),}\right.\\ &\left.{\left( {0.28,0.38,0.38,0.48;0.9} \right)} \right]\end{aligned}$$;

$$\begin{aligned}\tilde{A}_{34} &= \left[ {\left( {0.50,0.70,0.70,0.86;1} \right),}\right.\\ &\left.{\left( {0.60,0.70,0.70,0.78;0.9} \right)} \right]\end{aligned}$$.

$$\begin{aligned}\tilde{A}_{35} &= \left[ {\left( {0.34,0.54,0.54,0.72;1} \right),}\right.\\ &\left.{\left( {0.44,0.54,0.54,0.63;0.9} \right)} \right]\end{aligned}$$;

$$\begin{aligned}\tilde{A}_{36} &= \left[ {\left( {0.38,0.58,0.58,0.78;1} \right),}\right.\\ &\left.{\left( {0.48,0.58,0.58,0.68;0.9} \right)} \right]\end{aligned}$$.

$$\begin{aligned}\tilde{A}_{37} &= \left[ {\left( {0.50,0.70,0.70,0.90;1} \right),}\right.\\ &\left.{\left( {0.60,0.70,0.70,0.80;0.9} \right)} \right]\end{aligned}$$;

$$\begin{aligned}\tilde{A}_{38} &= \left[ {\left( {0.38,0.58,0.58,0.78;1} \right),}\right.\\ &\left.{\left( {0.48,0.58,0.58,0.68;0.9} \right)} \right]\end{aligned}$$.

$$\begin{aligned}\tilde{A}_{39} &= \left[ {\left( {0.22,0.42,0.42,0.62;1} \right),}\right.\\ &\left.{\left( {0.32,0.42,0.42,0.52;0.9} \right)} \right]\end{aligned}$$;

$$\begin{aligned}\tilde{A}_{310} &= \left[ {\left( {0.34,0.54,0.54,0.74;1} \right),}\right.\\ &\left.{\left( {0.44,0.54,0.54,0.64;0.9} \right)} \right]\end{aligned}$$.

$$\begin{aligned}\tilde{A}_{311} &= \left[ {\left( {0.58,0.78,0.78,0.94;1} \right),}\right.\\ &\left.{\left( {0.68,0.78,0.78,0.86;0.9} \right)} \right]\end{aligned}$$;

$$\begin{aligned}\tilde{A}_{312} &= \left[ {\left( {0.42,0.62,0.62,0.82;1} \right),}\right.\\ &\left.{\left( {0.52,0.62,0.62,0.72;0.9} \right)} \right]\end{aligned}$$.

$$\begin{aligned}\tilde{A}_{313} &= \left[ {\left( {0.54,0.74,0.74,0.92;1} \right),}\right.\\ &\left.{\left( {0.64,0.74,0.74,0.83;0.9} \right)} \right]\end{aligned}$$;

$$\begin{aligned}\tilde{A}_{314} &= \left[ {\left( {0.58,0.78,0.78,0.94;1} \right),}\right.\\ &\left.{\left( {0.68,0.78,0.78,0.86;0.9} \right)} \right]\end{aligned}$$.

$$\begin{aligned}\tilde{A}_{41}& = \left[ {\left( {0.00,0.00,0.00,0.10;1} \right),}\right.\\ &\left.{\left( {0.00,0.00,0.00,0.50;0.9} \right)} \right]\end{aligned}$$;

$$\begin{aligned}\tilde{A}_{42} &= \left[ {\left( {0.46,0.66,0.66,0.86;1} \right),}\right.\\ &\left.{\left( {0.56,0.66,0.66,0.76;0.9} \right)} \right]\end{aligned}$$.

$$\begin{aligned}\tilde{A}_{43} &= \left[ {\left( {0.34,0.54,0.54,0.74;1} \right),}\right.\\ &\left.{\left( {0.44,0.54,0.54,0.64;0.9} \right)} \right]\end{aligned}$$;

$$\begin{aligned}\tilde{A}_{44} &= \left[ {\left( {0.42,0.62,0.62,0.80;1} \right),}\right.\\ &\left.{\left( {0.52,0.62,0.62,0.71;0.9} \right)} \right]\end{aligned}$$.

$$\begin{aligned}\tilde{A}_{45} &= \left[ {\left( {0.20,0.38,0.38,0.58;1} \right),}\right.\\ &\left.{\left( {0.29,0.38,0.38,0.48;0.9} \right)} \right]\end{aligned}$$;

$$\begin{aligned}\tilde{A}_{46} &= \left[ {\left( {0.22,0.42,0.42,0.62;1} \right),}\right.\\ &\left.{\left( {0.32,0.42,0.42,0.52;0.9} \right)} \right]\end{aligned}$$.

$$\begin{aligned}\tilde{A}_{47} &= \left[ {\left( {0.58,0.78,0.78,0.92;1} \right),}\right.\\ &\left.{\left( {0.68,0.78,0.78,0.85;0.9} \right)} \right]\end{aligned}$$;

$$\begin{aligned}\tilde{A}_{48} &= \left[ {\left( {0.50,0.70,0.70,0.88;1} \right),}\right.\\ &\left.{\left( {0.60,0.70,0.70,0.79;0.9} \right)} \right]\end{aligned}$$.

$$\begin{aligned}\tilde{A}_{49} &= \left[ {\left( {0.22,0.42,0.42,0.62;1} \right),}\right.\\ &\left.{\left( {0.32,0.42,0.42,0.52;0.9} \right)} \right]\end{aligned}$$;

$$\begin{aligned}\tilde{A}_{410} &= \left[ {\left( {0.42,0.62,0.62,0.80;1} \right),}\right.\\ &\left.{\left( {0.52,0.62,0.62,0.71;0.9} \right)} \right]\end{aligned}$$.

$$\begin{aligned}\tilde{A}_{411} &= \left[ {\left( {0.62,0.82,0.82,0.94;1} \right),}\right.\\ &\left.{\left( {0.72,0.82,0.82,0.88;0.9} \right)} \right]\end{aligned}$$;

$$\begin{aligned}\tilde{A}_{412} &= \left[ {\left( {0.54,0.74,0.74,0.92;1} \right),}\right.\\ &\left.{\left( {0.64,0.74,0.74,0.83;0.9} \right)} \right]\end{aligned}$$.

$$\begin{aligned}\tilde{A}_{413} &= \left[ {\left( {0.58,0.78,0.78,0.94;1} \right),}\right.\\ &\left.{\left( {0.68,0.78,0.78,0.86;0.9} \right)} \right]\end{aligned}$$;

$$\begin{aligned}\tilde{A}_{414} &= \left[ {\left( {0.46,0.66,0.66,0.86;1} \right),}\right.\\ &\left.{\left( {0.56,0.66,0.66,0.76;0.9} \right)} \right]\end{aligned}$$.

$$\begin{aligned}\tilde{A}_{51} &= \left[ {\left( {0.00,0.00,0.00,0.10;1} \right),}\right.\\ &\left.{\left( {0.00,0.00,0.00,0.50;0.9} \right)} \right]\end{aligned}$$;

$$\begin{aligned}\tilde{A}_{52} &= \left[ {\left( {0.38,0.58,0.58,0.78;1} \right),}\right.\\ &\left.{\left( {0.48,0.58,0.58,0.68;0.9} \right)} \right]\end{aligned}$$.

$$\begin{aligned}\tilde{A}_{53} &= \left[ {\left( {0.40,0.58,0.58,0.74;1} \right),}\right.\\ &\left.{\left( {0.49,0.58,0.58,0.66;0.9} \right)} \right]\end{aligned}$$;

$$\begin{aligned}\tilde{A}_{54} &= \left[ {\left( {0.54,0.74,0.74,0.88;1} \right),}\right.\\ &\left.{\left( {0.64,0.74,0.74,0.81;0.9} \right)} \right]\end{aligned}$$.

$$\begin{aligned}\tilde{A}_{55} &= \left[ {\left( {0.46,0.66,0.66,0.86;1} \right),}\right.\\ &\left.{\left( {0.56,0.66,0.66,0.76;0.9} \right)} \right]\end{aligned}$$;

$$\begin{aligned}\tilde{A}_{56} &= \left[ {\left( {0.38,0.58,0.58,0.78;1} \right),}\right.\\ &\left.{\left( {0.48,0.58,0.58,0.68;0.9} \right)} \right]\end{aligned}$$.

$$\begin{aligned}\tilde{A}_{57} &= \left[ {\left( {0.08,0.26,0.26,0.46;1} \right),}\right.\\ &\left.{\left( {0.17,0.26,0.26,0.36;0.9} \right)} \right]\end{aligned}$$;

$$\begin{aligned}\tilde{A}_{58} &= \left[ {\left( {0.42,0.62,0.62,0.82;1} \right),}\right.\\ &\left.{\left( {0.52,0.62,0.62,0.72;0.9} \right)} \right]\end{aligned}$$.

$$\begin{aligned}\tilde{A}_{59} &= \left[ {\left( {0.16,0.34,0.34,0.54;1} \right),}\right.\\ &\left.{\left( {0.25,0.34,0.34,0.44;0.9} \right)} \right]\end{aligned}$$;

$$\begin{aligned}\tilde{A}_{510} &= \left[ {\left( {0.38,0.58,0.58,0.76;1} \right),}\right.\\ &\left.{\left( {0.48,0.58,0.58,0.67;0.9} \right)} \right]\end{aligned}$$.

$$\begin{aligned}\tilde{A}_{511} &= \left[ {\left( {0.66,0.86,0.86,0.98;1} \right),}\right.\\ &\left.{\left( {0.76,0.86,0.86,0.92;0.9} \right)} \right]\end{aligned}$$;

$$\begin{aligned}\tilde{A}_{512} &= \left[ {\left( {0.58,0.78,0.78,0.94;1} \right),}\right.\\ &\left.{\left( {0.68,0.78,0.78,0.86;0.9} \right)} \right]\end{aligned}$$.

$$\begin{aligned}\tilde{A}_{513} &= \left[ {\left( {0.66,0.86,0.86,0.98;1} \right),}\right.\\ &\left.{\left( {0.76,0.86,0.86,0.92;0.9} \right)} \right]\end{aligned}$$;

$$\begin{aligned}\tilde{A}_{514} &= \left[ {\left( {0.14,0.30,0.30,0.50;1} \right),}\right.\\ &\left.{\left( {0.22,0.30,0.30,0.40;0.9} \right)} \right]\end{aligned}$$.

#### Phase II: Determinate the weights of indicator

In this case, the PDs between any two indicators are measured by LTs, and the PRs based on the IT2FSs are constructed to get the weights of indicators.

Step 6: Establish the PRs matrix with LTs.

The same five experts are invited to give the PDs between two indicators by LTs. In particular, the LT $O = \left[ {\left( {1,1,1,1;1} \right),\left( {1,1,1,1;0.9} \right)} \right]$. The initial linguistic PRs matrixes are shown in Tables 30, 31, 32, 33, 34 (see the Appendix).

Next, their LTs are converted into IT2FSs, and the converted IT2FSs are aggregated by WA operator. In this case, the weights of the experts are not given, and it is supposed that $$w_{l} = {\raise0.7ex\hbox{$1$} \!\mathord{\left/ {\vphantom {1 5}}\right.\kern-\nulldelimiterspace} \!\lower0.7ex\hbox{$5$}}$$, $$\left( {l = 1,2, \cdots ,5} \right)$$. Then, the PRs matrix with IT2FSs can be obtained:

$$\begin{aligned}\tilde{A}_{11}^{\omega }& = \left[ {\left( {1,1,1,1;1} \right),}\right. \\ & \left.{\left( {1,1,1,1;0.9} \right)} \right]\end{aligned}$$

$$\begin{aligned}\tilde{A}_{12}^{\omega }& = \left[ {\left( {0.40,0.56,0.56,0.70;1} \right),}\right. \\ & \left.{\left( {0.48,0.56,0.56,0.63;0.9} \right)} \right]\end{aligned}$$.

$$\begin{aligned}\tilde{A}_{13}^{\omega }& = \left[ {\left( {0.20,0.36,0.36,0.54;1} \right),}\right. \\ & \left.{\left( {0.28,0.36,0.36,0.45;0.9} \right)} \right]\end{aligned}$$;

$$\begin{aligned}\tilde{A}_{14}^{\omega }& = \left[ {\left( {0.36,0.54,0.54,0.72;1} \right),}\right. \\ & \left.{\left( {0.45,0.54,0.54,0.63;0.9} \right)} \right]\end{aligned}$$.

$$\begin{aligned}\tilde{A}_{15}^{\omega }& = \left[ {\left( {0.12,0.26,0.26,0.46;1} \right),}\right. \\ & \left.{\left( {0.19,0.26,0.26,0.36;0.9} \right)} \right]\end{aligned}$$;

$$\begin{aligned}\tilde{A}_{16}^{\omega }& = \left[ {\left( {0.54,0.72,0.72,0.86;1} \right),}\right. \\ & \left.{\left( {0.63,0.72,0.72,0.79;0.9} \right)} \right]\end{aligned}$$.

$$\begin{aligned}\tilde{A}_{17}^{\omega }& = \left[ {\left( {0.44,0.60,0.60,0.76;1} \right),}\right. \\ & \left.{\left( {0.52,0.60,0.60,0.68;0.9} \right)} \right]\end{aligned}$$;

$$\begin{aligned}\tilde{A}_{18}^{\omega }& = \left[ {\left( {0.54,0.70,0.70,0.82;1} \right),}\right. \\ & \left.{\left( {0.62,0.70,0.70,0.76;0.9} \right)} \right]\end{aligned}$$.

$$\begin{aligned}\tilde{A}_{19}^{\omega }& = \left[ {\left( {0.22,0.36,0.36,0.54;1} \right),}\right. \\ & \left.{\left( {0.29,0.36,0.36,0.45;0.9} \right)} \right]\end{aligned}$$;

$$\begin{aligned}\tilde{A}_{110}^{\omega }& = \left[ {\left( {0.28,0.46,0.46,0.64;1} \right),}\right. \\ & \left.{\left( {0.37,0.46,0.46,0.55;0.9} \right)} \right]\end{aligned}$$.

$$\begin{aligned}\tilde{A}_{111}^{\omega }& = \left[ {\left( {0.42,0.60,0.60,0.76;1} \right),}\right. \\ & \left.{\left( {0.51,0.60,0.60,0.68;0.9} \right)} \right]\end{aligned}$$;

$$\begin{aligned}\tilde{A}_{112}^{\omega }& = \left[ {\left( {0.42,0.62,0.62,0.80;1} \right),}\right. \\ & \left.{\left( {0.52,0.62,0.62,0.71;0.9} \right)} \right]\end{aligned}$$.

$$\begin{aligned}\tilde{A}_{113}^{\omega }& = \left[ {\left( {0.50,0.68,0.68,0.84;1} \right),}\right. \\ & \left.{\left( {0.59,0.68,0.68,0.76;0.9} \right)} \right]\end{aligned}$$;

$$\begin{aligned}\tilde{A}_{114}^{\omega }& = \left[ {\left( {0.46,0.62,0.62,0.74;1} \right),}\right. \\ & \left.{\left( {0.54,0.62,0.62,0.68;0.9} \right)} \right]\end{aligned}$$.

$$\begin{aligned}\tilde{A}_{21}^{\omega }& = \left[ {\left( {0.30,0.44,0.44,0.60;1} \right),}\right. \\ & \left.{\left( {0.37,0.44,0.44,0.52;0.9} \right)} \right]\end{aligned}$$;

$$\begin{aligned}\tilde{A}_{22}^{\omega }& = \left[ {\left( {1,1,1,1;1} \right),}\right. \\ & \left.{\left( {1,1,1,1;0.9} \right)} \right]\end{aligned}$$.

$$\begin{aligned}\tilde{A}_{23}^{\omega }& = \left[ {\left( {0.34,0.54,0.54,0.74;1} \right),}\right. \\ & \left.{\left( {0.44,0.54,0.54,0.64;0.9} \right)} \right]\end{aligned}$$;

$$\begin{aligned}\tilde{A}_{24}^{\omega }& = \left[ {\left( {0.28,0.44,0.44,0.62;1} \right),}\right. \\ & \left.{\left( {0.36,0.44,0.44,0.53;0.9} \right)} \right]\end{aligned}$$.

$$\begin{aligned}\tilde{A}_{25}^{\omega }& = \left[ {\left( {0.38,0.58,0.58,0.78;1} \right),}\right. \\ & \left.{\left( {0.48,0.58,0.58,0.68;0.9} \right)} \right]\end{aligned}$$;

$$\begin{aligned}\tilde{A}_{26}^{\omega }& = \left[ {\left( {0.44,0.58,0.58,0.70;1} \right),}\right. \\ & \left.{\left( {0.51,0.58,0.58,0.64;0.9} \right)} \right]\end{aligned}$$.

$$\begin{aligned}\tilde{A}_{27}^{\omega }& = \left[ {\left( {0.50,0.60,0.60,0.68;1} \right),}\right. \\ & \left.{\left( {0.55,0.60,0.60,0.64;0.9} \right)} \right]\end{aligned}$$;

$$\begin{aligned}\tilde{A}_{28}^{\omega }& = \left[ {\left( {0.46,0.66,0.66,0.82;1} \right),}\right. \\ & \left.{\left( {0.56,0.66,0.66,0.74;0.9} \right)} \right]\end{aligned}$$.

$$\begin{aligned}\tilde{A}_{29}^{\omega }& = \left[ {\left( {0.36,0.54,0.54,0.72;1} \right),}\right. \\ & \left.{\left( {0.45,0.54,0.54,0.63;0.9} \right)} \right]\end{aligned}$$;

$$\begin{aligned}\tilde{A}_{210}^{\omega }& = \left[ {\left( {0.32,0.50,0.50,0.68;1} \right),}\right. \\ & \left.{\left( {0.41,0.50,0.50,0.59;0.9} \right)} \right]\end{aligned}$$.

$$\begin{aligned}\tilde{A}_{211}^{\omega }& = \left[ {\left( {0.38,0.58,0.58,0.76;1} \right),}\right. \\ & \left.{\left( {0.48,0.58,0.58,0.67;0.9} \right)} \right]\end{aligned}$$;

$$\begin{aligned}\tilde{A}_{212}^{\omega }& = \left[ {\left( {0.18,0.32,0.32,0.48;1} \right),}\right. \\ & \left.{\left( {0.25,0.32,0.32,0.40;0.9} \right)} \right]\end{aligned}$$.

$$\begin{aligned}\tilde{A}_{213}^{\omega }& = \left[ {\left( {0.44,0.60,0.60,0.76;1} \right),}\right. \\ & \left.{\left( {0.52,0.60,0.60,0.68;0.9} \right)} \right]\end{aligned}$$;

$$\begin{aligned}\tilde{A}_{214}^{\omega }& = \left[ {\left( {0.26,0.42,0.42,0.60;1} \right),}\right. \\ & \left.{\left( {0.34,0.42,0.42,0.51;0.9} \right)} \right]\end{aligned}$$.

$$\begin{aligned}\tilde{A}_{31}^{\omega }& = \left[ {\left( {0.38,0.56,0.56,0.72;1} \right),}\right. \\ & \left.{\left( {0.47,0.56,0.56,0.64;0.9} \right)} \right]\end{aligned}$$;

$$\begin{aligned}\tilde{A}_{32}^{\omega }& = \left[ {\left( {0.26,0.46,0.46,0.66;1} \right),}\right. \\ & \left.{\left( {0.36,0.46,0.46,0.56;0.9} \right)} \right]\end{aligned}$$.

$$\begin{aligned}\tilde{A}_{33}^{\omega }& = \left[ {\left( {1,1,1,1;1} \right),}\right. \\ & \left.{\left( {1,1,1,1;0.9} \right)} \right]\end{aligned}$$;

$$\begin{aligned}\tilde{A}_{34}^{\omega }& = \left[ {\left( {0.26,0.40,0.40,0.56;1} \right),}\right. \\ & \left.{\left( {0.33,0.40,0.40,0.48;0.9} \right)} \right]\end{aligned}$$.

$$\begin{aligned}\tilde{A}_{35}^{\omega }& = \left[ {\left( {0.30,0.44,0.44,0.60;1} \right),}\right. \\ & \left.{\left( {0.37,0.44,0.44,0.52;0.9} \right)} \right]\end{aligned}$$;

$$\begin{aligned}\tilde{A}_{36}^{\omega }& = \left[ {\left( {0.46,0.66,0.66,0.82;1} \right),}\right. \\ & \left.{\left( {0.56,0.66,0.66,0.74;0.9} \right)} \right]\end{aligned}$$.

$$\begin{aligned}\tilde{A}_{37}^{\omega }& = \left[ {\left( {0.54,0.72,0.72,0.88;1} \right),}\right. \\ & \left.{\left( {0.63,0.72,0.72,0.80;0.9} \right)} \right]\end{aligned}$$;

$$\begin{aligned}\tilde{A}_{38}^{\omega }& = \left[ {\left( {0.48,0.62,0.62,0.74;1} \right),}\right. \\ & \left.{\left( {0.55,0.62,0.62,0.68;0.9} \right)} \right]\end{aligned}$$.

$$\begin{aligned}\tilde{A}_{39}^{\omega }& = \left[ {\left( {0.28,0.46,0.46,0.64;1} \right),}\right. \\ & \left.{\left( {0.37,0.46,0.46,0.55;0.9} \right)} \right]\end{aligned}$$;

$$\begin{aligned}\tilde{A}_{310}^{\omega }& = \left[ {\left( {0.38,0.52,0.52,0.66;1} \right),}\right. \\ & \left.{\left( {0.45,0.52,0.52,0.59;0.9} \right)} \right]\end{aligned}$$.

$$\begin{aligned}\tilde{A}_{311}^{\omega }& = \left[ {\left( {0.38,0.56,0.56,0.72;1} \right),}\right. \\ & \left.{\left( {0.47,0.56,0.56,0.64;0.9} \right)} \right]\end{aligned}$$;

$$\begin{aligned}\tilde{A}_{312}^{\omega }& = \left[ {\left( {0.50,0.70,0.70,0.88;1} \right),}\right. \\ & \left.{\left( {0.60,0.70,0.70,0.79;0.9} \right)} \right]\end{aligned}$$.

$$\begin{aligned}\tilde{A}_{313}^{\omega }& = \left[ {\left( {0.38,0.56,0.56,0.72;1} \right),}\right. \\ & \left.{\left( {0.47,0.56,0.56,0.64;0.9} \right)} \right]\end{aligned}$$;

$$\begin{aligned}\tilde{A}_{314}^{\omega }& = \left[ {\left( {0.38,0.52,0.52,0.68;1} \right),}\right. \\ & \left.{\left( {0.45,0.52,0.52,0.60;0.9} \right)} \right]\end{aligned}$$.

$$\begin{aligned}\tilde{A}_{41}^{\omega }& = \left[ {\left( {0.28,0.46,0.46,0.64;1} \right),}\right. \\ & \left.{\left( {0.37,0.46,0.46,0.55;0.9} \right)} \right]\end{aligned}$$;

$$\begin{aligned}\tilde{A}_{42}^{\omega }& = \left[ {\left( {0.38,0.56,0.56,0.72;1} \right),}\right. \\ & \left.{\left( {0.47,0.56,0.56,0.64;0.9} \right)} \right]\end{aligned}$$.

$$\begin{aligned}\tilde{A}_{43}^{\omega }& = \left[ {\left( {0.44,0.60,0.60,0.74;1} \right),}\right. \\ & \left.{\left( {0.52,0.60,0.60,0.67;0.9} \right)} \right]\end{aligned}$$;

$$\begin{aligned}\tilde{A}_{44}^{\omega }& = \left[ {\left( {1,1,1,1;1} \right),}\right. \\ & \left.{\left( {1,1,1,1;0.9} \right)} \right]\end{aligned}$$.

$$\begin{aligned}\tilde{A}_{45}^{\omega }& = \left[ {\left( {0.58,0.78,0.78,0.92;1} \right),}\right. \\ & \left.{\left( {0.68,0.78,0.78,0.85;0.9} \right)} \right]\end{aligned}$$;

$$\begin{aligned}\tilde{A}_{46}^{\omega }& = \left[ {\left( {0.30,0.46,0.46,0.64;1} \right),}\right. \\ & \left.{\left( {0.38,0.46,0.46,0.55;0.9} \right)} \right]\end{aligned}$$.

$$\begin{aligned}\tilde{A}_{47}^{\omega }& = \left[ {\left( {0.20,0.38,0.38,0.58;1} \right),}\right. \\ & \left.{\left( {0.29,0.38,0.38,0.48;0.9} \right)} \right]\end{aligned}$$;

$$\begin{aligned}\tilde{A}_{48}^{\omega }& = \left[ {\left( {0.40,0.56,0.56,0.72;1} \right),}\right. \\ & \left.{\left( {0.48,0.56,0.56,0.64;0.9} \right)} \right]\end{aligned}$$.

$$\begin{aligned}\tilde{A}_{49}^{\omega }& = \left[ {\left( {0.54,0.74,0.74,0.88;1} \right),}\right. \\ & \left.{\left( {0.64,0.74,0.74,0.81;0.9} \right)} \right]\end{aligned}$$;

$$\begin{aligned}\tilde{A}_{410}^{\omega }& = \left[ {\left( {0.36,0.54,0.54,0.72;1} \right),}\right. \\ & \left.{\left( {0.45,0.54,0.54,0.63;0.9} \right)} \right]\end{aligned}$$.

$$\begin{aligned}\tilde{A}_{411}^{\omega }& = \left[ {\left( {0.36,0.50,0.50,0.64;1} \right),}\right. \\ & \left.{\left( {0.43,0.50,0.50,0.57;0.9} \right)} \right]\end{aligned}$$;

$$\begin{aligned}\tilde{A}_{412}^{\omega }& = \left[ {\left( {0.52,0.68,0.68,0.82;1} \right),}\right. \\ & \left.{\left( {0.60,0.68,0.68,0.75;0.9} \right)} \right]\end{aligned}$$.

$$\begin{aligned}\tilde{A}_{413}^{\omega }& = \left[ {\left( {0.62,0.80,0.80,0.92;1} \right),}\right. \\ & \left.{\left( {0.71,0.80,0.80,0.86;0.9} \right)} \right]\end{aligned}$$;

$$\begin{aligned}\tilde{A}_{414}^{\omega }& = \left[ {\left( {0.30,0.42,0.42,0.56;1} \right),}\right. \\ & \left.{\left( {0.36,0.42,0.42,0.49;0.9} \right)} \right]\end{aligned}$$.

$$\begin{aligned}\tilde{A}_{51}^{\omega }& = \left[ {\left( {0.46,0.66,0.66,0.80;1} \right),}\right. \\ & \left.{\left( {0.56,0.66,0.66,0.73;0.9} \right)} \right]\end{aligned}$$;

$$\begin{aligned}\tilde{A}_{52}^{\omega }& = \left[ {\left( {0.22,0.42,0.42,0.62;1} \right),}\right. \\ & \left.{\left( {0.32,0.42,0.42,0.52;0.9} \right)} \right]\end{aligned}$$.

$$\begin{aligned}\tilde{A}_{53}^{\omega }& = \left[ {\left( {0.40,0.56,0.56,0.70;1} \right),}\right. \\ & \left.{\left( {0.48,0.56,0.56,0.63;0.9} \right)} \right]\end{aligned}$$;

$$\begin{aligned}\tilde{A}_{54}^{\omega }& = \left[ {\left( {0.08,0.22,0.22,0.42;1} \right),}\right. \\ & \left.{\left( {0.15,0.22,0.22,0.32;0.9} \right)} \right]\end{aligned}$$.

$$\begin{aligned}\tilde{A}_{55}^{\omega }& = \left[ {\left( {1,1,1,1;1} \right),}\right. \\ & \left.{\left( {1,1,1,1;0.9} \right)} \right]\end{aligned}$$;

$$\begin{aligned}\tilde{A}_{56}^{\omega }& = \left[ {\left( {0.50,0.68,0.68,0.82;1} \right),}\right. \\ & \left.{\left( {0.59,0.68,0.68,0.75;0.9} \right)} \right]\end{aligned}$$.

$$\begin{aligned}\tilde{A}_{57}^{\omega }& = \left[ {\left( {0.32,0.48,0.48,0.64;1} \right),}\right. \\ & \left.{\left( {0.36,0.48,0.48,0.56;0.9} \right)} \right]\end{aligned}$$;

$$\begin{aligned}\tilde{A}_{58}^{\omega }& = \left[ {\left( {0.36,0.54,0.54,0.72;1} \right),}\right. \\ & \left.{\left( {0.45,0.54,0.54,0.63;0.9} \right)} \right]\end{aligned}$$.

$$\begin{aligned}\tilde{A}_{59}^{\omega }& = \left[ {\left( {0.16,0.32,0.32,0.50;1} \right),}\right. \\ & \left.{\left( {0.24,0.32,0.32,0.41;0.9} \right)} \right]\end{aligned}$$;

$$\begin{aligned}\tilde{A}_{510}^{\omega }& = \left[ {\left( {0.32,0.48,0.48,0.64;1} \right),}\right. \\ & \left.{\left( {0.40,0.48,0.48,0.56;0.9} \right)} \right]\end{aligned}$$.

$$\begin{aligned}\tilde{A}_{511}^{\omega }& = \left[ {\left( {0.34,0.46,0.46,0.60;1} \right),}\right. \\ & \left.{\left( {0.40,0.46,0.46,0.53;0.9} \right)} \right]\end{aligned}$$;

$$\begin{aligned}\tilde{A}_{512}^{\omega }& = \left[ {\left( {0.24,0.42,0.42,0.62;1} \right),}\right. \\ & \left.{\left( {0.33,0.42,0.42,0.52;0.9} \right)} \right]\end{aligned}$$.

$$\begin{aligned}\tilde{A}_{513}^{\omega }& = \left[ {\left( {0.40,0.56,0.56,0.70;1} \right),}\right. \\ & \left.{\left( {0.48,0.56,0.56,0.63;0.9} \right)} \right]\end{aligned}$$;

$$\begin{aligned}\tilde{A}_{514}^{\omega }& = \left[ {\left( {0.22,0.42,0.42,0.62;1} \right),}\right. \\ & \left.{\left( {0.32,0.42,0.42,0.52;0.9} \right)} \right]\end{aligned}$$.

$$\begin{aligned}\tilde{A}_{61}^{\omega }& = \left[ {\left( {0.14,0.28,0.28,0.46;1} \right),}\right. \\ & \left.{\left( {0.21,0.28,0.28,0.37;0.9} \right)} \right]\end{aligned}$$;

$$\begin{aligned}\tilde{A}_{62}^{\omega }& = \left[ {\left( {0.30,0.42,0.42,0.56;1} \right),}\right.\\ & \left.{42\left( {0.36,0.,0.42,0.49;0.9} \right)} \right]\end{aligned}$$.

$$\begin{aligned}\tilde{A}_{63}^{\omega }& = \left[ {\left( {0.18,0.34,0.34,0.54;1} \right),}\right. \\ & \left.{\left( {0.26,0.34,0.34,0.44;0.9} \right)} \right]\end{aligned}$$;

$$\begin{aligned}\tilde{A}_{64}^{\omega }& = \left[ {\left( {0.36,0.54,0.54,0.70;1} \right),}\right. \\ & \left.{\left( {0.45,0.54,0.54,0.62;0.9} \right)} \right]\end{aligned}$$.

$$\begin{aligned}\tilde{A}_{65}^{\omega }& = \left[ {\left( {0.18,0.32,0.32,0.50;1} \right),}\right. \\ & \left.{\left( {0.25,0.32,0.32,0.41;0.9} \right)} \right]\end{aligned}$$;

$$\begin{aligned}\tilde{A}_{66}^{\omega }& = \left[ {\left( {1,1,1,1;1} \right),}\right. \\ & \left.{\left( {1,1,1,1;0.9} \right)} \right]\end{aligned}$$.

$$\begin{aligned}\tilde{A}_{67}^{\omega }& = \left[ {\left( {0.44,0.62,0.62,0.78;1} \right),}\right. \\ & \left.{\left( {0.53,0.62,0.62,0.70;0.9} \right)} \right]\end{aligned}$$;

$$\begin{aligned}\tilde{A}_{68}^{\omega }& = \left[ {\left( {0.44,0.60,0.60,0.74;1} \right),}\right. \\ & \left.{\left( {0.52,0.60,0.60,0.67;0.9} \right)} \right]\end{aligned}$$.

$$\begin{aligned}\tilde{A}_{69}^{\omega }& = \left[ {\left( {0.52,0.66,0.66,0.78;1} \right),}\right. \\ & \left.{\left( {0.59,0.66,0.66,0.72;0.9} \right)} \right]\end{aligned}$$;

$$\begin{aligned}\tilde{A}_{610}^{\omega }& = \left[ {\left( {0.30,0.44,0.44,0.60;1} \right),}\right. \\ & \left.{\left( {0.37,0.44,0.44,0.52;0.9} \right)} \right]\end{aligned}$$.

$$\begin{aligned}\tilde{A}_{611}^{\omega }& = \left[ {\left( {0.28,0.44,0.44,0.60;1} \right),}\right. \\ & \left.{\left( {0.36,0.44,0.44,0.52;0.9} \right)} \right]\end{aligned}$$;

$$\begin{aligned}\tilde{A}_{612}^{\omega }& = \left[ {\left( {0.36,0.54,0.54,0.72;1} \right),}\right. \\ & \left.{\left( {0.45,0.54,0.54,0.63;0.9} \right)} \right]\end{aligned}$$.

$$\begin{aligned}\tilde{A}_{613}^{\omega }& = \left[ {\left( {0.42,0.62,0.62,0.80;1} \right),}\right. \\ & \left.{\left( {0.52,0.62,0.62,0.71;0.9} \right)} \right]\end{aligned}$$;

$$\begin{aligned}\tilde{A}_{614}^{\omega }& = \left[ {\left( {0.44,0.62,0.62,0.78;1} \right),}\right. \\ & \left.{\left( {0.53,0.62,0.62,0.70;0.9} \right)} \right]\end{aligned}$$.

$$\begin{aligned}\tilde{A}_{71}^{\omega }& = \left[ {\left( {0.22,0.34,0.34,0.48;1} \right),}\right. \\ & \left.{\left( {0.28,0.34,0.34,0.41;0.9} \right)} \right]\end{aligned}$$;

$$\begin{aligned}\tilde{A}_{72}^{\omega }& = \left[ {\left( {0.32,0.40,0.40,0.50;1} \right),}\right. \\ & \left.{\left( {0.36,0.40,0.40,0.54;0.9} \right)} \right]\end{aligned}$$.

$$\begin{aligned}\tilde{A}_{73}^{\omega }& = \left[ {\left( {0.12,0.28,0.28,0.46;1} \right),}\right. \\ & \left.{\left( {0.20,0.28,0.28,0.37;0.9} \right)} \right]\end{aligned}$$;

$$\begin{aligned}\tilde{A}_{74}^{\omega }& = \left[ {\left( {0.42,0.62,0.62,0.80;1} \right),}\right. \\ & \left.{\left( {0.52,0.62,0.62,0.71;0.9} \right)} \right]\end{aligned}$$.

$$\begin{aligned}\tilde{A}_{75}^{\omega }& = \left[ {\left( {0.36,0.52,0.52,0.68;1} \right),}\right. \\ & \left.{\left( {0.44,0.52,0.52,0.60;0.9} \right)} \right]\end{aligned}$$;

$$\begin{aligned}\tilde{A}_{76}^{\omega }& = \left[ {\left( {0.22,0.38,0.38,0.56;1} \right),}\right. \\ & \left.{\left( {0.30,0.38,0.38,0.47;0.9} \right)} \right]\end{aligned}$$.

$$\begin{aligned}\tilde{A}_{77}^{\omega }& = \left[ {\left( {1,1,1,1;1} \right),}\right. \\ & \left.{\left( {1,1,1,1;0.9} \right)} \right]\end{aligned}$$;

$$\begin{aligned}\tilde{A}_{78}^{\omega }& = \left[ {\left( {0.54,0.72,0.72,0.86;1} \right),}\right. \\ & \left.{\left( {0.63,0.72,0.72,0.79;0.9} \right)} \right]\end{aligned}$$.

$$\begin{aligned}\tilde{A}_{79}^{\omega }& = \left[ {\left( {0.42,0.62,0.62,0.80;1} \right),}\right. \\ & \left.{\left( {0.52,0.62,0.62,0.71;0.9} \right)} \right]\end{aligned}$$;

$$\begin{aligned}\tilde{A}_{710}^{\omega }& = \left[ {\left( {0.36,0.54,0.54,0.72;1} \right),}\right. \\ & \left.{\left( {0.45,0.54,0.54,0.63;0.9} \right)} \right]\end{aligned}$$.

$$\begin{aligned}\tilde{A}_{711}^{\omega }& = \left[ {\left( {0.46,0.64,0.64,0.78;1} \right),}\right. \\ & \left.{\left( {0.55,0.64,0.64,0.71;0.9} \right)} \right]\end{aligned}$$;

$$\begin{aligned}\tilde{A}_{712}^{\omega }& = \left[ {\left( {0.20,0.32,0.32,0.50;1} \right),}\right. \\ & \left.{\left( {0.26,0.32,0.32,0.41;0.9} \right)} \right]\end{aligned}$$.

$$\begin{aligned}\tilde{A}_{713}^{\omega }& = \left[ {\left( {0.30,0.50,0.50,0.70;1} \right),}\right. \\ & \left.{\left( {0.40,0.50,0.50,0.60;0.9} \right)} \right]\end{aligned}$$;

$$\begin{aligned}\tilde{A}_{714}^{\omega }& = \left[ {\left( {0.42,0.58,0.58,0.70;1} \right),}\right. \\ & \left.{\left( {0.50,0.58,0.58,0.64;0.9} \right)} \right]\end{aligned}$$.

$$\begin{aligned}\tilde{A}_{81}^{\omega }& = \left[ {\left( {0.36,0.50,0.50,0.64;1} \right),}\right. \\ & \left.{\left( {0.43,0.50,0.50,0.57;0.9} \right)} \right]\end{aligned}$$;

$$\begin{aligned}\tilde{A}_{82}^{\omega }& = \left[ {\left( {0.18,0.34,0.34,0.54;1} \right),}\right. \\ & \left.{\left( {0.26,0.34,0.34,0.44;0.9} \right)} \right]\end{aligned}$$.

$$\begin{aligned}\tilde{A}_{83}^{\omega }& = \left[ {\left( {0.26,0.38,0.38,0.52;1} \right),}\right. \\ & \left.{\left( {0.32,0.38,0.38,0.54;0.9} \right)} \right]\end{aligned}$$;

$$\begin{aligned}\tilde{A}_{84}^{\omega }& = \left[ {\left( {0.28,0.44,0.44,0.60;1} \right),}\right. \\ & \left.{\left( {0.36,0.44,0.44,0.52;0.9} \right)} \right]\end{aligned}$$.

$$\begin{aligned}\tilde{A}_{85}^{\omega }& = \left[ {\left( {0.28,0.46,0.46,0.64;1} \right),}\right. \\ & \left.{\left( {0.37,0.46,0.46,0.55;0.9} \right)} \right]\end{aligned}$$;

$$\begin{aligned}\tilde{A}_{86}^{\omega }& = \left[ {\left( {0.26,0.40,0.40,0.56;1} \right),}\right. \\ & \left.{\left( {0.33,0.40,0.40,0.48;0.9} \right)} \right]\end{aligned}$$.

$$\begin{aligned}\tilde{A}_{87}^{\omega }& = \left[ {\left( {0.14,0.28,0.28,0.46;1} \right),}\right. \\ & \left.{\left( {0.21,0.28,0.28,0.37;0.9} \right)} \right]\end{aligned}$$;

$$\begin{aligned}\tilde{A}_{88}^{\omega }& = \left[ {\left( {1,1,1,1;1} \right),}\right. \\ & \left.{\left( {1,1,1,1;0.9} \right)} \right]\end{aligned}$$.

$$\begin{aligned}\tilde{A}_{89}^{\omega }& = \left[ {\left( {0.20,0.38,0.38,0.58;1} \right),}\right. \\ & \left.{\left( {0.29,0.38,0.38,0.48;0.9} \right)} \right]\end{aligned}$$;

$$\begin{aligned}\tilde{A}_{810}^{\omega }& = \left[ {\left( {0.28,0.46,0.46,0.66;1} \right),}\right. \\ & \left.{\left( {0.37,0.46,0.46,0.56;0.9} \right)} \right]\end{aligned}$$.

$$\begin{aligned}\tilde{A}_{811}^{\omega }& = \left[ {\left( {0.36,0.42,0.42,0.50;1} \right),}\right. \\ & \left.{\left( {0.39,0.42,0.42,0.46;0.9} \right)} \right]\end{aligned}$$;

$$\begin{aligned}\tilde{A}_{812}^{\omega }& = \left[ {\left( {0.30,0.42,0.42,0.58;1} \right),}\right. \\ & \left.{\left( {0.36,0.42,0.42,0.50;0.9} \right)} \right]\end{aligned}$$.

$$\begin{aligned}\tilde{A}_{813}^{\omega }& = \left[ {\left( {0.16,0.32,0.32,0.50;1} \right),}\right. \\ & \left.{\left( {0.24,0.32,0.32,0.41;0.9} \right)} \right]\end{aligned}$$;

$$\begin{aligned}\tilde{A}_{814}^{\omega }& = \left[ {\left( {0.26,0.46,0.46,0.66;1} \right),}\right. \\ & \left.{\left( {0.36,0.46,0.46,0.56;0.9} \right)} \right]\end{aligned}$$.

$$\begin{aligned}\tilde{A}_{91}^{\omega }& = \left[ {\left( {0.28,0.46,0.46,0.64;1} \right),}\right. \\ & \left.{\left( {0.37,0.46,0.46,0.55;0.9} \right)} \right]\end{aligned}$$;

$$\begin{aligned}\tilde{A}_{92}^{\omega }& = \left[ {\left( {0.28,0.46,0.46,0.64;1} \right),}\right. \\ & \left.{\left( {0.37,0.46,0.46,0.55;0.9} \right)} \right]\end{aligned}$$.

$$\begin{aligned}\tilde{A}_{93}^{\omega }& = \left[ {\left( {0.28,0.46,0.46,0.64;1} \right),}\right. \\ & \left.{\left( {0.37,0.46,0.46,0.55;0.9} \right)} \right]\end{aligned}$$;

$$\begin{aligned}\tilde{A}_{94}^{\omega }& = \left[ {\left( {0.12,0.26,0.26,0.46;1} \right),}\right. \\ & \left.{\left( {0.19,0.26,0.26,0.36;0.9} \right)} \right]\end{aligned}$$.

$$\begin{aligned}\tilde{A}_{95}^{\omega }& = \left[ {\left( {0.40,0.54,0.54,0.68;1} \right),}\right. \\ & \left.{\left( {0.47,0.54,0.54,0.61;0.9} \right)} \right]\end{aligned}$$;

$$\begin{aligned}\tilde{A}_{96}^{\omega }& = \left[ {\left( {0.22,0.34,0.34,0.48;1} \right),}\right. \\ & \left.{\left( {0.28,0.34,0.34,0.41;0.9} \right)} \right]\end{aligned}$$.

$$\begin{aligned}\tilde{A}_{97}^{\omega }& = \left[ {\left( {0.20,0.38,0.38,0.58;1} \right),}\right. \\ & \left.{\left( {0.29,0.38,0.38,0.48;0.9} \right)} \right]\end{aligned}$$;

$$\begin{aligned}\tilde{A}_{98}^{\omega }& = \left[ {\left( {0.42,0.62,0.62,0.80;1} \right),}\right. \\ & \left.{\left( {0.52,0.62,0.62,0.71;0.9} \right)} \right]\end{aligned}$$.

$$\begin{aligned}\tilde{A}_{99}^{\omega }& = \left[ {\left( {1,1,1,1;1} \right),}\right. \\ & \left.{\left( {1,1,1,1;0.9} \right)} \right]\end{aligned}$$;

$$\begin{aligned}\tilde{A}_{910}^{\omega }& = \left[ {\left( {0.32,0.48,0.48,0.64;1} \right),}\right. \\ & \left.{\left( {0.40,0.48,0.48,0.56;0.9} \right)} \right]\end{aligned}$$.

$$\begin{aligned}\tilde{A}_{911}^{\omega }& = \left[ {\left( {0.46,0.66,0.66,0.82;1} \right),}\right. \\ & \left.{\left( {0.56,0.66,0.66,0.74;0.9} \right)} \right]\end{aligned}$$;

$$\begin{aligned}\tilde{A}_{912}^{\omega }& = \left[ {\left( {0.48,0.66,0.66,0.82;1} \right),}\right. \\ & \left.{\left( {0.57,0.66,0.66,0.74;0.9} \right)} \right]\end{aligned}$$.

$$\begin{aligned}\tilde{A}_{913}^{\omega }& = \left[ {\left( {0.20,0.38,0.38,0.58;1} \right),}\right. \\ & \left.{\left( {0.29,0.38,0.38,0.48;0.9} \right)} \right]\end{aligned}$$;

$$\begin{aligned}\tilde{A}_{914}^{\omega }& = \left[ {\left( {0.32,0.50,0.50,0.70;1} \right),}\right. \\ & \left.{\left( {0.41,0.50,0.50,0.60;0.9} \right)} \right]\end{aligned}$$.

$$\begin{aligned}\tilde{A}_{101}^{\omega }& = \left[ {\left( {0.36,0.52,0.52,0.68;1} \right),}\right. \\ & \left.{\left( {0.44,0.52,0.52,0.60;0.9} \right)} \right]\end{aligned}$$;

$$\begin{aligned}\tilde{A}_{102}^{\omega }& = \left[ {\left( {0.32,0.50,0.50,0.68;1} \right),}\right. \\ & \left.{\left( {0.41,0.50,0.50,0.59;0.9} \right)} \right]\end{aligned}$$.

$$\begin{aligned}\tilde{A}_{103}^{\omega }& = \left[ {\left( {0.34,0.48,0.48,0.62;1} \right),}\right. \\ & \left.{\left( {0.41,0.48,0.48,0.55;0.9} \right)} \right]\end{aligned}$$;

$$\begin{aligned}\tilde{A}_{104}^{\omega }& = \left[ {\left( {0.28,0.46,0.46,0.64;1} \right),}\right. \\ & \left.{\left( {0.37,0.46,0.46,0.55;0.9} \right)} \right]\end{aligned}$$.

$$\begin{aligned}\tilde{A}_{105}^{\omega }& = \left[ {\left( {0.36,0.52,0.52,0.68;1} \right),}\right. \\ & \left.{\left( {0.44,0.52,0.52,0.60;0.9} \right)} \right]\end{aligned}$$;

$$\begin{aligned}\tilde{A}_{106}^{\omega }& = \left[ {\left( {0.40,0.56,0.56,0.70;1} \right),}\right. \\ & \left.{\left( {0.48,0.56,0.56,0.63;0.9} \right)} \right]\end{aligned}$$.

$$\begin{aligned}\tilde{A}_{107}^{\omega }& = \left[ {\left( {0.24,0.42,0.42,0.62;1} \right),}\right. \\ & \left.{\left( {0.33,0.42,0.42,0.52;0.9} \right)} \right]\end{aligned}$$;

$$\begin{aligned}\tilde{A}_{108}^{\omega }& = \left[ {\left( {0.34,0.54,0.54,0.72;1} \right),}\right. \\ & \left.{\left( {0.44,0.54,0.54,0.63;0.9} \right)} \right]\end{aligned}$$.

$$\begin{aligned}\tilde{A}_{109}^{\omega }& = \left[ {\left( {0.36,0.52,0.52,0.68;1} \right),}\right. \\ & \left.{\left( {0.44,0.52,0.52,0.60;0.9} \right)} \right]\end{aligned}$$;

$$\begin{aligned}\tilde{A}_{1010}^{\omega }& = \left[ {\left( {1,1,1,1;1} \right),}\right. \\ & \left.{\left( {1,1,1,1;0.9} \right)} \right]\end{aligned}$$.

$$\begin{aligned}\tilde{A}_{1011}^{\omega }& = \left[ {\left( {0.44,0.62,0.62,0.80;1} \right),}\right. \\ & \left.{\left( {0.53,0.62,0.62,0.71;0.9} \right)} \right]\end{aligned}$$;

$$\begin{aligned}\tilde{A}_{1012}^{\omega }& = \left[ {\left( {0.54,0.72,0.72,0.86;1} \right),}\right. \\ & \left.{\left( {0.63,0.72,0.72,0.79;0.9} \right)} \right]\end{aligned}$$.

$$\begin{aligned}\tilde{A}_{1013}^{\omega }& = \left[ {\left( {0.44,0.60,0.60,0.74;1} \right),}\right. \\ & \left.{\left( {0.52,0.60,0.60,0.67;0.9} \right)} \right]\end{aligned}$$;

$$\begin{aligned}\tilde{A}_{1014}^{\omega }& = \left[ {\left( {0.26,0.46,0.46,0.66;1} \right),}\right. \\ & \left.{\left( {0.36,0.46,0.46,0.56;0.9} \right)} \right]\end{aligned}$$.

$$\begin{aligned}\tilde{A}_{111}^{\omega }& = \left[ {\left( {0.38,0.58,0.58,0.76;1} \right),}\right. \\ & \left.{\left( {0.48,0.58,0.58,0.67;0.9} \right)} \right]\end{aligned}$$;

$$\begin{aligned}\tilde{A}_{112}^{\omega }& = \left[ {\left( {0.24,0.42,0.42,0.62;1} \right),}\right. \\ & \left.{\left( {0.33,0.42,0.42,0.52;0.9} \right)} \right]\end{aligned}$$.

$$\begin{aligned}\tilde{A}_{113}^{\omega }& = \left[ {\left( {0.28,0.44,0.44,0.62;1} \right),}\right. \\ & \left.{\left( {0.36,0.44,0.44,0.53;0.9} \right)} \right]\end{aligned}$$;

$$\begin{aligned}\tilde{A}_{114}^{\omega }& = \left[ {\left( {0.36,0.50,0.50,0.64;1} \right),}\right. \\ & \left.{\left( {0.43,0.50,0.50,0.57;0.9} \right)} \right]\end{aligned}$$.

$$\begin{aligned}\tilde{A}_{115}^{\omega }& = \left[ {\left( {0.40,0.54,0.54,0.66;1} \right),}\right. \\ & \left.{\left( {0.47,0.54,0.54,0.60;0.9} \right)} \right]\end{aligned}$$;

$$\begin{aligned}\tilde{A}_{116}^{\omega }& = \left[ {\left( {0.40,0.56,0.56,0.72;1} \right),}\right. \\ & \left.{\left( {0.48,0.56,0.56,0.64;0.9} \right)} \right]\end{aligned}$$.

$$\begin{aligned}\tilde{A}_{117}^{\omega }& = \left[ {\left( {0.26,0.40,0.40,0.56;1} \right),}\right. \\ & \left.{\left( {0.33,0.40,0.40,0.48;0.9} \right)} \right]\end{aligned}$$;

$$\begin{aligned}\tilde{A}_{118}^{\omega }& = \left[ {\left( {0.50,0.58,0.58,0.64;1} \right),}\right. \\ & \left.{\left( {0.54,0.58,0.58,0.61;0.9} \right)} \right]\end{aligned}$$.

$$\begin{aligned}\tilde{A}_{119}^{\omega }& = \left[ {\left( {0.18,0.34,0.34,0.54;1} \right),}\right. \\ & \left.{\left( {0.26,0.34,0.34,0.44;0.9} \right)} \right]\end{aligned}$$;

$$\begin{aligned}\tilde{A}_{1110}^{\omega }& = \left[ {\left( {0.20,0.38,0.38,0.56;1} \right),}\right. \\ & \left.{\left( {0.29,0.38,0.38,0.47;0.9} \right)} \right]\end{aligned}$$.

$$\begin{aligned}\tilde{A}_{1111}^{\omega }& = \left[ {\left( {1,1,1,1;1} \right),}\right. \\ & \left.{\left( {1,1,1,1;0.9} \right)} \right]\end{aligned}$$;

$$\begin{aligned}\tilde{A}_{1112}^{\omega }& = \left[ {\left( {0.48,0.64,0.64,0.78;1} \right),}\right. \\ & \left.{\left( {0.56,0.64,0.64,0.71;0.9} \right)} \right]\end{aligned}$$.

$$\begin{aligned}\tilde{A}_{1113}^{\omega }& = \left[ {\left( {0.30,0.50,0.50,0.70;1} \right),}\right. \\ & \left.{\left( {0.40,0.50,0.50,0.60;0.9} \right)} \right]\end{aligned}$$;

$$\begin{aligned}\tilde{A}_{1114}^{\omega }& = \left[ {\left( {0.54,0.72,0.72,0.84;1} \right),}\right. \\ & \left.{\left( {0.63,0.72,0.72,0.78;0.9} \right)} \right]\end{aligned}$$.

$$\begin{aligned}\tilde{A}_{121}^{\omega }& = \left[ {\left( {0.20,0.38,0.38,0.58;1} \right),}\right. \\ & \left.{\left( {0.29,0.38,0.38,0.48;0.9} \right)} \right]\end{aligned}$$;

$$\begin{aligned}\tilde{A}_{122}^{\omega }& = \left[ {\left( {0.52,0.68,0.68,0.82;1} \right),}\right. \\ & \left.{\left( {0.60,0.68,0.68,0.75;0.9} \right)} \right]\end{aligned}$$.

$$\begin{aligned}\tilde{A}_{123}^{\omega }& = \left[ {\left( {0.12,0.30,0.30,0.50;1} \right),}\right. \\ & \left.{\left( {0.21,0.30,0.30,0.40;0.9} \right)} \right]\end{aligned}$$;

$$\begin{aligned}\tilde{A}_{124}^{\omega }& = \left[ {\left( {0.18,0.32,0.32,0.48;1} \right),}\right. \\ & \left.{\left( {0.25,0.32,0.32,0.40;0.9} \right)} \right]\end{aligned}$$.

$$\begin{aligned}\tilde{A}_{125}^{\omega }& = \left[ {\left( {0.38,0.58,0.58,0.76;1} \right),}\right. \\ & \left.{\left( {0.48,0.58,0.58,0.67;0.9} \right)} \right]\end{aligned}$$;

$$\begin{aligned}\tilde{A}_{126}^{\omega }& = \left[ {\left( {0.28,0.46,0.46,0.64;1} \right),}\right. \\ & \left.{\left( {0.37,0.46,0.46,0.55;0.9} \right)} \right]\end{aligned}$$.

$$\begin{aligned}\tilde{A}_{127}^{\omega }& = \left[ {\left( {0.50,0.68,0.68,0.80;1} \right),}\right. \\ & \left.{\left( {0.59,0.68,0.68,0.74;0.9} \right)} \right]\end{aligned}$$;

$$\begin{aligned}\tilde{A}_{128}^{\omega }& = \left[ {\left( {0.42,0.58,0.58,0.70;1} \right),}\right. \\ & \left.{\left( {0.50,0.58,0.58,0.64;0.9} \right)} \right]\end{aligned}$$.

$$\begin{aligned}\tilde{A}_{129}^{\omega }& = \left[ {\left( {0.18,0.34,0.34,0.52;1} \right),}\right. \\ & \left.{\left( {0.26,0.34,0.34,0.43;0.9} \right)} \right]\end{aligned}$$;

$$\begin{aligned}\tilde{A}_{1210}^{\omega }& = \left[ {\left( {0.14,0.28,0.28,0.46;1} \right),}\right. \\ & \left.{\left( {0.21,0.28,0.28,0.37;0.9} \right)} \right]\end{aligned}$$.

$$\begin{aligned}\tilde{A}_{1211}^{\omega }& = \left[ {\left( {0.22,0.36,0.36,0.52;1} \right),}\right. \\ & \left.{\left( {0.29,0.36,0.36,0.44;0.9} \right)} \right]\end{aligned}$$;

$$\begin{aligned}\tilde{A}_{1212}^{\omega }& = \left[ {\left( {1,1,1,1;1} \right),}\right. \\ & \left.{\left( {1,1,1,1;0.9} \right)} \right]\end{aligned}$$.

$$\begin{aligned}\tilde{A}_{1213}^{\omega }& = \left[ {\left( {0.30,0.44,0.44,0.60;1} \right),}\right. \\ & \left.{\left( {0.37,0.44,0.44,0.52;0.9} \right)} \right]\end{aligned}$$;

$$\begin{aligned}\tilde{A}_{1214}^{\omega }& = \left[ {\left( {0.54,0.72,0.72,0.88;1} \right),}\right. \\ & \left.{\left( {0.62,0.72,0.72,0.80;0.9} \right)} \right]\end{aligned}$$.

$$\begin{aligned}\tilde{A}_{131}^{\omega }& = \left[ {\left( {0.16,0.32,0.32,0.50;1} \right),}\right. \\ & \left.{\left( {0.24,0.32,0.32,0.41;0.9} \right)} \right]\end{aligned}$$;

$$\begin{aligned}\tilde{A}_{132}^{\omega }& = \left[ {\left( {0.24,0.40,0.40,0.56;1} \right),}\right. \\ & \left.{\left( {0.32,0.40,0.40,0.48;0.9} \right)} \right]\end{aligned}$$.

$$\begin{aligned}\tilde{A}_{133}^{\omega }& = \left[ {\left( {0.28,0.44,0.44,0.62;1} \right),}\right. \\ & \left.{\left( {0.36,0.44,0.44,0.53;0.9} \right)} \right]\end{aligned}$$;

$$\begin{aligned}\tilde{A}_{134}^{\omega }& = \left[ {\left( {0.08,0.20,0.20,0.38;1} \right),}\right. \\ & \left.{\left( {0.14,0.20,0.20,0.29;0.9} \right)} \right]\end{aligned}$$.

$$\begin{aligned}\tilde{A}_{135}^{\omega }& = \left[ {\left( {0.30,0.44,0.44,0.60;1} \right),}\right. \\ & \left.{\left( {0.37,0.44,0.44,0.52;0.9} \right)} \right]\end{aligned}$$;

$$\begin{aligned}\tilde{A}_{136}^{\omega }& = \left[ {\left( {0.20,0.38,0.38,0.58;1} \right),}\right. \\ & \left.{\left( {0.29,0.38,0.38,0.48;0.9} \right)} \right]\end{aligned}$$.

$$\begin{aligned}\tilde{A}_{137}^{\omega }& = \left[ {\left( {0.30,0.50,0.50,0.70;1} \right),}\right. \\ & \left.{\left( {0.40,0.50,0.50,0.60;0.9} \right)} \right]\end{aligned}$$;

$$\begin{aligned}\tilde{A}_{138}^{\omega }& = \left[ {\left( {0.50,0.68,0.68,0.84;1} \right),}\right. \\ & \left.{\left( {0.59,0.68,0.68,0.76;0.9} \right)} \right]\end{aligned}$$.

$$\begin{aligned}\tilde{A}_{139}^{\omega }& = \left[ {\left( {0.42,0.62,0.62,0.80;1} \right),}\right. \\ & \left.{\left( {0.52,0.62,0.62,0.71;0.9} \right)} \right]\end{aligned}$$;

$$\begin{aligned}\tilde{A}_{1310}^{\omega }& = \left[ {\left( {0.26,0.40,0.40,0.56;1} \right),}\right. \\ & \left.{\left( {0.33,0.40,0.40,0.48;0.9} \right)} \right]\end{aligned}$$.

$$\begin{aligned}\tilde{A}_{1311}^{\omega }& = \left[ {\left( {0.30,0.50,0.50,0.70;1} \right),}\right. \\ & \left.{\left( {0.40,0.50,0.50,0.60;0.9} \right)} \right]\end{aligned}$$;

$$\begin{aligned}\tilde{A}_{1312}^{\omega }& = \left[ {\left( {0.40,0.56,0.56,0.70;1} \right),}\right. \\ & \left.{\left( {0.48,0.56,0.56,0.63;0.9} \right)} \right]\end{aligned}$$.

$$\begin{aligned}\tilde{A}_{1313}^{\omega }& = \left[ {\left( {1,1,1,1;1} \right),}\right. \\ & \left.{\left( {1,1,1,1;0.9} \right)} \right]\end{aligned}$$;

$$\begin{aligned}\tilde{A}_{1314}^{\omega }& = \left[ {\left( {0.22,0.36,0.36,0.54;1} \right),}\right. \\ & \left.{\left( {0.29,0.36,0.36,0.45;0.9} \right)} \right]\end{aligned}$$.

$$\begin{aligned}\tilde{A}_{141}^{\omega }& = \left[ {\left( {0.26,0.38,0.38,0.54;1} \right),}\right. \\ & \left.{\left( {0.32,0.38,0.38,0.55;0.9} \right)} \right]\end{aligned}$$;

$$\begin{aligned}\tilde{A}_{142}^{\omega }& = \left[ {\left( {0.40,0.58,0.58,0.74;1} \right),}\right. \\ & \left.{\left( {0.49,0.58,0.58,0.66;0.9} \right)} \right]\end{aligned}$$.

$$\begin{aligned}\tilde{A}_{143}^{\omega }& = \left[ {\left( {0.32,0.48,0.48,0.62;1} \right),}\right. \\ & \left.{\left( {0.40,0.48,0.48,0.55;0.9} \right)} \right]\end{aligned}$$;

$$\begin{aligned}\tilde{A}_{144}^{\omega }& = \left[ {\left( {0.44,0.58,0.58,0.70;1} \right),}\right. \\ & \left.{\left( {0.51,0.58,0.58,0.64;0.9} \right)} \right]\end{aligned}$$.

$$\begin{aligned}\tilde{A}_{145}^{\omega }& = \left[ {\left( {0.38,0.58,0.58,0.78;1} \right),}\right. \\ & \left.{\left( {0.48,0.58,0.58,0.68;0.9} \right)} \right]\end{aligned}$$;

$$\begin{aligned}\tilde{A}_{146}^{\omega }& = \left[ {\left( {0.22,0.38,0.38,0.56;1} \right),}\right. \\ & \left.{\left( {0.30,0.38,0.38,0.47;0.9} \right)} \right]\end{aligned}$$.

$$\begin{aligned}\tilde{A}_{147}^{\omega }& = \left[ {\left( {0.30,0.42,0.42,0.58;1} \right),}\right. \\ & \left.{\left( {0.36,0.42,0.42,0.50;0.9} \right)} \right]\end{aligned}$$;

$$\begin{aligned}\tilde{A}_{148}^{\omega }& = \left[ {\left( {0.34,0.54,0.54,0.74;1} \right),}\right. \\ & \left.{\left( {0.44,0.54,0.54,0.64;0.9} \right)} \right]\end{aligned}$$.

$$\begin{aligned}\tilde{A}_{149}^{\omega }& = \left[ {\left( {0.30,0.50,0.50,0.68;1} \right),}\right. \\ & \left.{\left( {0.40,0.50,0.50,0.59;0.9} \right)} \right]\end{aligned}$$;

$$\begin{aligned}\tilde{A}_{1410}^{\omega }& = \left[ {\left( {0.34,0.54,0.54,0.74;1} \right),}\right. \\ & \left.{\left( {0.44,0.54,0.54,0.64;0.9} \right)} \right]\end{aligned}$$.

$$\begin{aligned}\tilde{A}_{1411}^{\omega }& = \left[ {\left( {0.16,0.28,0.28,0.46;1} \right),}\right. \\ & \left.{\left( {0.22,0.28,0.28,0.37;0.9} \right)} \right]\end{aligned}$$;

$$\begin{aligned}\tilde{A}_{1412}^{\omega }& = \left[ {\left( {0.12,0.28,0.28,0.46;1} \right),}\right. \\ & \left.{\left( {0.20,0.28,0.28,0.37;0.9} \right)} \right]\end{aligned}$$.

$$\begin{aligned}\tilde{A}_{1413}^{\omega }& = \left[ {\left( {0.46,0.64,0.64,0.78;1} \right),}\right. \\ & \left.{\left( {0.55,0.64,0.64,0.71;0.9} \right)} \right]\end{aligned}$$;

$$\begin{aligned}\tilde{A}_{1414}^{\omega }& = \left[ {\left( {1,1,1,1;1} \right),}\right. \\ & \left.{\left( {1,1,1,1;0.9} \right)} \right]\end{aligned}$$.

Step 7: Compute the PDs of one indicator over the others.

The PD of indicator $C_{j} \left( {j = 1,2, \cdots ,14} \right)$ over the others can be computed by collecting the all elements (except $C_{jj}$) in the $i$ th row of matrix $\tilde{A}^{\omega }$ based on the Eq. (26).

$$ \begin{aligned}  \tilde{A}_{1}^{\omega } &= IT2FPA\left( {\tilde{A}_{12}^{\omega } ,\tilde{A}_{13}^{\omega } , \cdots ,\tilde{A}_{114}^{\omega } } \right)\\
& \quad  = \left[ \left( {0.3536,0.4759,0.4759,0.5348;1} \right),\right.\\
& \qquad\left. \left( {0.4013,0.4759,0.4759,0.5928;0.9} \right) \right]\end{aligned} $$

$$ \begin{aligned}
\tilde{A}_{2}^{\omega }& = IT2FPA\left( {\tilde{A}_{21}^{\omega } ,\tilde{A}_{23}^{\omega } , \cdots ,\tilde{A}_{214}^{\omega } } \right)\\
&\quad  = \left[ \left( {0.3316,0.4008,0.4008,0.4953;1} \right),\right.\\
&\qquad \left.\left( {0.3028,0.4008,0.4008,0.4518;0.9} \right) \right]
\end{aligned} $$

$$ \begin{aligned}
\tilde{A}_{4}^{\omega } &= IT2FPA\left( {\tilde{A}_{41}^{\omega } ,\tilde{A}_{42}^{\omega } , \cdots ,\tilde{A}_{414}^{\omega } } \right)\\
&\quad  = \left[ \left( {0.1426,0.2325,0.2325,0.2971;1} \right),\right.\\
&\qquad \left.\left( {0.1668,0.2325,0.2325,0.2719;0.9} \right) \right]
\end{aligned} $$

$$ \begin{aligned}
\tilde{A}_{12}^{\omega } &= IT2FPA\left( {\tilde{A}_{121}^{\omega } ,\tilde{A}_{122}^{\omega } , \cdots ,\tilde{A}_{1214}^{\omega } } \right) \\
&\quad = \left[ \left( 0.2954,0.3995,0.3995,0.4218;1 \right),\right.\\
&\qquad \left. \left( {0.3149,0.3995,0.3995,0.4027;0.9} \right) \right]
\end{aligned} $$

$$ \begin{aligned}
\tilde{A}_{13}^{\omega } &= IT2FPA\left( {\tilde{A}_{131}^{\omega } ,\tilde{A}_{132}^{\omega } , \cdots ,\tilde{A}_{1314}^{\omega } } \right)\\
&\quad  = \left[ \left( {0.3665,0.4957,0.4957,0.5418;1} \right),\right.\\
&\qquad \left.\left( {0.3948,0.4957,0.4957,0.5173;0.9} \right) \right]
\end{aligned} $$

$$ \begin{aligned}
\tilde{A}_{14}^{\omega } &= IT2FPA\left( {\tilde{A}_{141}^{\omega } ,\tilde{A}_{142}^{\omega } , \cdots ,\tilde{A}_{1413}^{\omega } } \right)\\
&\quad  = \left[ \left( {0.1764,0.2718,0.2718,0.3284;1} \right),\right.\\
&\qquad \left.\left( {0.2046,0.2718,0.2718,0.3083;0.9} \right) \right]
\end{aligned}$$

Step 8: Compute the weight of indicator.

Based on Eq. (13), the likelihood matrix of the indicator preference is obtained which shown in Table 6.

**Table 6:** The likelihood matrix of the indicator preference

$I\left( {\tilde{A}_{i}^{\omega } \ge \tilde{A}_{j}^{\omega } } \right)$	$\tilde{A}_{1}^{\omega }$	$\tilde{A}_{2}^{\omega }$	$\tilde{A}_{3}^{\omega }$	$\tilde{A}_{4}^{\omega }$	$\tilde{A}_{5}^{\omega }$	$\tilde{A}_{6}^{\omega }$	$\tilde{A}_{7}^{\omega }$
$\tilde{A}_{1}^{\omega }$	0.5000	0.7913	0.9173	0.9698	0.5653	0.4145	0.9022
$\tilde{A}_{2}^{\omega }$	0.3488	0.5000	0.8129	0.9159	0.3406	0.2626	0.7755
$\tilde{A}_{3}^{\omega }$	0.1834	0.2967	0.5000	0.8217	0.1779	0.1483	0.4719
$\tilde{A}_{4}^{\omega }$	0.1043	0.1692	0.2785	0.5000	0.1066	0.0876	0.2265
$\tilde{A}_{5}^{\omega }$	0.6289	0.7910	0.9235	0.9693	0.5000	0.4261	0.9087
$\tilde{A}_{6}^{\omega }$	0.7583	0.8616	0.9461	0.9823	0.7260	0.5000	0.9383
$\tilde{A}_{7}^{\omega }$	0.2161	0.3390	0.6723	0.8731	0.1996	0.1622	0.5000
$\tilde{A}_{8}^{\omega }$	0.8260	0.9079	0.9664	0.9965	0.8096	0.7157	0.9610
$\tilde{A}_{9}^{\omega }$	0.4291	0.6699	0.8867	0.9578	0.3980	0.2903	0.8624
$\tilde{A}_{10}^{\omega }$	0.5141	0.7322	0.9067	0.9617	0.4741	0.3423	0.8890
$\tilde{A}_{11}^{\omega }$	0.8397	0.9162	0.9702	0.9996	0.8256	0.7342	0.9651
$\tilde{A}_{12}^{\omega }$	0.2655	0.4577	0.7802	0.9136	0.2572	0.1985	0.7323
$\tilde{A}_{13}^{\omega }$	0.5766	0.7660	0.9127	0.9653	0.5273	0.3870	0.8961
$\tilde{A}_{14}^{\omega }$	0.1287	0.2071	0.3753	0.7018	0.1319	0.1091	0.3079
$I\left( {\tilde{A}_{i}^{\omega } \ge \tilde{A}_{j}^{\omega } } \right)$	$\tilde{A}_{8}^{\omega }$	$\tilde{A}_{9}^{\omega }$	$\tilde{A}_{10}^{\omega }$	$\tilde{A}_{11}^{\omega }$	$\tilde{A}_{12}^{\omega }$	$\tilde{A}_{13}^{\omega }$	$\tilde{A}_{14}^{\omega }$
$\tilde{A}_{1}^{\omega }$	0.3294	0.7329	0.6568	0.3159	0.8606	0.6145	0.9559
$\tilde{A}_{2}^{\omega }$	0.2128	0.4438	0.3853	0.2053	0.6500	0.3674	0.8882
$\tilde{A}_{3}^{\omega }$	0.1241	0.2308	0.2022	0.1209	0.3456	0.1915	0.7415
$\tilde{A}_{4}^{\omega }$	0.0701	0.1308	0.1211	0.0672	0.1773	0.1126	0.4043
$\tilde{A}_{5}^{\omega }$	0.3377	0.7444	0.6663	0.3218	0.8666	0.6265	0.9549
$\tilde{A}_{6}^{\omega }$	0.4480	0.8412	0.7957	0.4315	0.9161	0.7611	0.9710
$\tilde{A}_{7}^{\omega }$	0.1355	0.2634	0.2269	0.1320	0.4030	0.2154	0.8081
$\tilde{A}_{8}^{\omega }$	0.5000	0.8965	0.8655	0.5627	0.9460	0.8353	0.9874
$\tilde{A}_{9}^{\omega }$	0.2311	0.5000	0.4607	0.2206	0.7849	0.4394	0.9378
$\tilde{A}_{10}^{\omega }$	0.2710	0.6656	0.5000	0.2544	0.8307	0.5263	0.9441
$\tilde{A}_{11}^{\omega }$	0.6062	0.9075	0.8817	0.5000	0.9509	0.8509	0.9908
$\tilde{A}_{12}^{\omega }$	0.1646	0.3541	0.2992	0.1606	0.5000	0.2793	0.8765
$\tilde{A}_{13}^{\omega }$	0.3074	0.7069	0.6122	0.2917	0.8480	0.5000	0.9498
$\tilde{A}_{14}^{\omega }$	0.0888	0.1634	0.1504	0.0850	0.2264	0.1393	0.5000

Based on the likelihood of two PDs between two indicators $I\left( {\tilde{A}_{i}^{\omega } \ge \tilde{A}_{j}^{\omega } } \right)$, the weight of indicator $C_{i} \left( {i = 1,2, \cdots ,14} \right)$ can be computed by applying Eq. (27) as shown in Table 7.

**Table 7:** The weight of indicator

$\omega_{j}$	$\omega_{1}$	$\omega_{2}$	$\omega_{3}$	$\omega_{4}$	$\omega_{5}$	$\omega_{6}$	$\omega_{7}$
	0.0875	0.0653	0.0419	0.0235	0.0888	0.0999	0.0473
$\omega_{j}$	$\omega_{8}$	$\omega_{9}$	$\omega_{10}$	$\omega_{11}$	$\omega_{12}$	$\omega_{13}$	$\omega_{14}$
	0.1082	0.0741	0.0810	0.1097	0.0573	0.0850	0.0305

#### Phase III: Obtain the ranking result

Step 9: Calculate the GPS $\tilde{G}\left( {A_{ij} } \right)$ of alternative $X_{i}$ with respect to the indicator $C_{j}$.

By applying Eq.  (28), the maximum IT2FS $\tilde{A}_{{_{j} }}^{ + }$ of alternative $X_{i}$ with respect to the indicator $C_{j}$ can be acquired. And by applying Eq. (29), the weight of the most significant indicator $C_{j}$ can be acquired. By applying Eq. (10), the $d\left( {\tilde{A}_{ij} ,\tilde{A}_{{_{j} }}^{ + } } \right)$ can be acquired, which shown in Table 8. At the same time, the $d\left( {\omega_{j} ,\omega^{ + } } \right)$ can also be acquired, which shown in Table 9.

**Table 8:** The $d\left( {\tilde{A}_{ij} ,\tilde{A}_{{_{j} }}^{ + } } \right)$ between each city and best one on each indicator

$d\left( {\tilde{A}_{ij} ,\tilde{A}_{{_{j} }}^{ + } } \right)$	$C_{1}$	$C_{2}$	$C_{3}$	$C_{4}$	$C_{5}$	$C_{6}$	$C_{7}$
$X_{1}$	0.0000	0.0000	0.0800	0.0000	0.0000	0.0000	0.1531
$X_{2}$	0.2942	0.3456	0.0000	0.2110	0.2267	0.3087	0.0000
$X_{3}$	0.6867	0.1543	0.2400	0.1741	0.2696	0.3087	0.1107
$X_{4}$	0.9634	0.2667	0.0800	0.2506	0.4221	0.4674	0.0400
$X_{5}$	0.9634	0.3456	0.0470	0.1373	0.1476	0.3087	0.5454
$d\left( {\tilde{A}_{ij} ,\tilde{A}_{{_{j} }}^{ + } } \right)$	$C_{8}$	$C_{9}$	$C_{10}$	$C_{11}$	$C_{12}$	$C_{13}$	$C_{14}$
$X_{1}$	0.0766	0.0000	0.0766	0.4658	0.1930	0.4259	0.0000
$X_{2}$	0.0000	0.1134	0.0000	0.3063	0.0738	0.2267	0.0247
$X_{3}$	0.1930	0.3893	0.2665	0.0738	0.1531	0.1107	0.0229
$X_{4}$	0.0766	0.3893	0.3860	0.0400	0.0369	0.0738	0.1346
$X_{5}$	0.1531	0.4654	0.4620	0.0000	0.0000	0.0000	0.4854

**Table 9:** The $d\left( {\omega_{j} ,\omega^{ + } } \right)$ between each weight of indicator and the best one

$\omega_{j}$	$\omega_{1}$	$\omega_{2}$	$\omega_{3}$	$\omega_{4}$	$\omega_{5}$	$\omega_{6}$	$\omega_{7}$
$d\left( {\omega_{j} ,\omega^{ + } } \right)$	0.0222	0.0444	0.0678	0.0862	0.0209	0.0098	0.0624
$\omega_{j}$	$\omega_{8}$	$\omega_{9}$	$\omega_{10}$	$\omega_{11}$	$\omega_{12}$	$\omega_{13}$	$\omega_{14}$
$d\left( {\omega_{j} ,\omega^{ + } } \right)$	0.0015	0.0356	0.0287	0.0000	0.0523	0.0247	0.0792

Without losing of generality, suppose $\rho = 0.5$. Then, by applying Eq. (30), the GPS $\tilde{G}\left( {X_{ij} } \right)$ can be calculated, which shown in Table 10.

**Table 10:** The GP scores of cities

$\tilde{G}\left( {X_{ij} } \right)$	$C_{1}$	$C_{2}$	$C_{3}$	$C_{4}$	$C_{5}$	$C_{6}$	$C_{7}$
$X_{1}$	0.0157	0.0314	0.0742	0.0610	0.0148	0.0069	0.1169
$X_{2}$	0.2086	0.2464	0.0479	0.1612	0.1610	0.2184	0.0441
$X_{3}$	0.4858	0.1135	0.1763	0.1374	0.1912	0.2184	0.0899
$X_{4}$	0.6814	0.1912	0.0742	0.1874	0.2988	0.3306	0.2524
$X_{5}$	0.6814	0.2464	0.0583	0.1146	0.1054	0.2184	0.3881
$\tilde{G}\left( {X_{ij} } \right)$	$C_{8}$	$C_{9}$	$C_{10}$	$C_{11}$	$C_{12}$	$C_{13}$	$C_{14}$
$X_{1}$	0.0542	0.0252	0.0578	0.3294	0.1414	0.3017	0.0560
$X_{2}$	0.0011	0.0840	0.0203	0.2166	0.0640	0.1613	0.0587
$X_{3}$	0.1365	0.2764	0.1895	0.0522	0.1144	0.0802	0.0583
$X_{4}$	0.0542	0.2764	0.2737	0.0283	0.0453	0.0550	0.1104
$X_{5}$	0.1083	0.3300	0.3273	0.0000	0.0370	0.0175	0.3478

Step 10: Establish the global WR.

By applying Eq. (31), the average PD of the alternative $X_{i}$ can be defined as follows:

$$\tilde{\tilde{R}}\left( {X_{1} } \right) = 0.0919$$, $$\tilde{\tilde{R}}\left( {X_{2} } \right) = 0.1210$$, $$\tilde{\tilde{R}}\left( {X_{3} } \right) = 0.1657$$, $$\tilde{\tilde{R}}\left( {X_{4} } \right) = 0.1899$$, $$\tilde{\tilde{R}}\left( {X_{5} } \right) = 0.2129$$.

Then, the WR can be obtained as follows: $X_{1} \succ X_{2} \succ X_{3} \succ \, X_{4} \succ X_{5}$.

Step 11: Construct the PIR structure of alternatives $X_{i} \left( {i = 1,2, \cdots ,5} \right)$.

1. Calculate the PIs.

By applying Eq. (32) and Eq. (33), the average PI of $X_{i}$ over $X_{\kappa }$ with respect to $C_{j}$ can be obtained, which shown in Table 11.

**Table 11:** The average PI of $X_{i}$ over $X_{\kappa }$ with respect to $C_{j}$

$\tilde{P}\left( {X_{i} ,X_{\kappa } } \right)$	$\tilde{P}\left( {X_{1} ,X_{\kappa } } \right)$	$\tilde{P}\left( {X_{2} ,X_{\kappa } } \right)$	$\tilde{P}\left( {X_{3} ,X_{\kappa } } \right)$	$\tilde{P}\left( {X_{4} ,X_{\kappa } } \right)$	$\tilde{P}\left( {X_{5} ,X_{\kappa } } \right)$
$X_{1}$	0.0000	0.0216	0.0395	0.0460	0.0524
$X_{2}$	0.0662	0.0000	0.0288	0.0263	0.0350
$X_{3}$	0.1133	0.0735	0.0000	0.0216	0.0319
$X_{4}$	0.1515	0.1096	0.0601	0.0000	0.0335
$X_{5}$	0.1531	0.1230	0.0791	0.0421	0.0000

By applying Eq. (34), the net PI of $X_{i}$ over $X_{\kappa }$ with respect to $C_{j}$ can be acquired, which shown in Table 12.

(2) Determine the preference threshold (PT) and the indifference threshold (IT).

**Table 12:** The net PI of $X_{i}$ over $X_{\kappa }$ with respect to $C_{j}$

$\Delta \tilde{P}\left( {X_{i} ,X_{\kappa } } \right)$	$\Delta \tilde{P}\left( {X_{1} ,X_{\kappa } } \right)$	$\Delta \tilde{P}\left( {X_{2} ,X_{\kappa } } \right)$	$\Delta \tilde{P}\left( {X_{3} ,X_{\kappa } } \right)$	$\Delta \tilde{P}\left( {X_{4} ,X_{\kappa } } \right)$	$\Delta \tilde{P}\left( {X_{5} ,X_{\kappa } } \right)$
$X_{1}$	0.0000	− 0.0446	− 0.0738	− 0.1055	− 0.0907
$X_{2}$	0.0446	0.0000	− 0.0447	− 0.0833	− 0.0880
$X_{3}$	0.0738	0.0447	0.0000	− 0.0385	− 0.0472
$X_{4}$	0.1055	0.0833	0.0385	0.0000	− 0.0086
$X_{5}$	0.0907	0.0880	0.0472	0.0086	0.0000

Without loss of generality, let $\upsilon = 0.9$, based on the distance between two adjacent interval type-2 fuzzy numbers shown in Table 3, then $\gamma \in \left[ {0,0.105} \right]$. Let $\gamma = 0.02$, by applying Eq. (35) and Eq. (36), then $\varepsilon = 0.00143$,$\lambda = 0.01$.

Step 12: Obtain the strong ranking based on the WR and the PIR structure.

The rank of RER of cities under the stress of COVID-19 are obtained as: $X_{1} \succ X_{2} \succ X_{3} \succ \, X_{4} \succ X_{5}$. Obviously, the strong ranking result is the same as the weak ranking.

(3) Construct the PIR structure.

Based on the PT $\varepsilon$ and IT $\lambda$, the PIR structure is determined as shown in Table 13.

**Table 13:** The PIR structure of pairwise cities

PIR	$X_{1}$	$X_{2}$	$X_{3}$	$X_{4}$	$X_{5}$
$X_{1}$	–	$>$	$>$	$>$	$>$
$X_{2}$	$<$	–	$>$	$>$	$>$
$X_{3}$	$<$	$<$	–	$>$	$>$
$X_{4}$	$<$	$<$	$<$	–	$>$
$X_{5}$	$<$	$<$	$<$	$<$	–

Based on the weight of each indicator in Table 5, the four most significance indicator are epidemic prevention and control efforts ($\tilde{C}_{11}$), intensity of credit support ($\tilde{C}_{8}$), industrial clusters competitiveness ($\tilde{C}_{6}$) and industrial chain system ($\tilde{C}_{5}$). By means of inquiring experts, this result is consistent with real case. Because epidemic prevention and control is the foundation and guarantee for the rapid recovery of regional economy. The epidemic has interrupted the business plans of enterprises and made them face difficulties in capital turnover. Credit support is an important means to maintain the normal operation of enterprises. At the same time, the impact of COVID-19 on regional economic development is typical of external shocks, so the more stable the industrial chain, the stronger the competitiveness of industrial clusters, the stronger the ability of regional economy to resist external shocks. At present, based on perfect industrial chain and strong industrial cluster competitiveness, city $X_{1}$ has the highest level of regional economic development. Therefore, the ranking result accords with reality.

More importantly, the above ranking result can provide some valuable reference information for government departments at all levels with targeted operation. They can formulate targeted economic recovery policies according to the regional economic recovery capacity of each city.

### Exploration of effects of parameters $\gamma$, $\varepsilon$ and $\lambda$ on final ranking results for this case

Furthermore, for exploring the effects of the parameters $\gamma$, $\varepsilon$ and $\lambda$ on ranking results in this real case, based on the range of $\gamma \in \left[ {0,0.105} \right]$, distinct parameter $\gamma$ can be assigned to acquire the parameters $\varepsilon$ and $\lambda$ in five scenarios, and the corresponding results are shown in Table 14.

**Table 14:** Parameters of three thresholds in five scenarios

	Scenario 1	Scenario 2	Scenario 3	Scenario 4	Scenario 5
$\gamma$	0.02	0.04	0.06	0.08	0.10
$\varepsilon$	0.00143	0.00286	0.00429	0.00571	0.00714
$\lambda$	0.01	0.02	0.03	0.04	0.05

By applying the developed IT2F-ORESTE method, the PIR structure between cities can be acquired according with the three thresholds ($\gamma$, $\varepsilon$ and $\lambda$). In five different scenarios, the cities ranking based on the corresponding PIR structure all remain $X_{1} \succ X_{2} \succ X_{3} \succ \, X_{4} \succ X_{5}$. Though with the gradual increase of $\gamma$, the values of $\varepsilon$ and $\lambda$ change accordingly, the ranking results demonstrate a better degree of stability. That is, in this case (*n* = 14), the values of parameters $\gamma$, $\varepsilon$ and $\lambda$ may have no important effect on the ranking result.

### Comparison analyses with the traditional ORESTE method

In this section, the case is solved by the traditional ORESTE method and the comparison are made with the IT2F-ORESTE method to demonstrate the superiority of the developed new method.

Step 1: Aggregate global preference scores (GPS).

Assume that $R_{j}$ is the original ranking of the important degree of criterion $C_{j} \left( {j = 1,2, \cdots ,14} \right)$ and $R_{j} \left( {X_{i} } \right)$ is the original ranking of the preference value of alternative $X_{i} \left( {i = 1,2, \cdots ,5} \right)$ under criterion $C_{j}$. The results are shown in Table 15.

**Table 15:** The initial Besson^’^s ranking of indicators and cities

	$C_{1}$	$C_{2}$	$C_{3}$	$C_{4}$	$C_{5}$	$C_{6}$	$C_{7}$
	5	9	12	14	4	3	11
$X_{1}$	1	1	4	1	1	1	4
$X_{2}$	2	3	1	2	2	2	1
$X_{3}$	3	2	3	5	4	3	3
$X_{4}$	4	4	4	4	5	5	2
$X_{5}$	4	5	2	3	3	3	5

	$C_{8}$	$C_{9}$	$C_{10}$	$C_{11}$	$C_{12}$	$C_{13}$	$C_{14}$
	2	8	7	1	10	6	13
$X_{1}$	2	2	2	5	5	5	1
$X_{2}$	1	1	1	4	1	2	2
$X_{3}$	5	4	5	3	4	4	3
$X_{4}$	2	3	3	2	3	3	4
$X_{5}$	4	5	4	1	2	1	5

Let $\eta = 0.5$, the GPS can be aggregated by applying Eq. (15), and which is shown in Table 16.

**Table 16:** GPS under each indicator

	$C_{1}$	$C_{2}$	$C_{3}$	$C_{4}$	$C_{5}$	$C_{6}$	$C_{7}$
$X_{1}$	3.6056	6.4031	8.9443	9.9247	2.9155	2.2361	8.2765
$X_{2}$	3.8079	6.7082	8.5147	10.0000	3.1623	2.5495	7.8102
$X_{3}$	4.1231	6.5192	8.7464	10.5119	4.0000	3.0000	8.0623
$X_{4}$	4.5277	6.9642	8.9442	10.2956	4.5277	4.1231	7.9057
$X_{5}$	4.5277	7.2801	8.6023	10.1242	3.5355	3.0000	8.5440

	$C_{8}$	$C_{9}$	$C_{10}$	$C_{11}$	$C_{12}$	$C_{13}$	$C_{14}$
$X_{1}$	2.0000	5.8310	5.1478	3.6056	7.9057	5.5227	9.2195
$X_{2}$	1.5811	5.7009	5.0000	2.9155	7.1063	4.4721	9.3005
$X_{3}$	3.8079	6.3246	6.0828	2.2361	7.6158	5.0990	9.4340
$X_{4}$	2.0000	6.0415	5.3852	1.5811	7.3824	4.7434	9.6177
$X_{5}$	3.1623	6.6708	5.7009	1.0000	7.2111	4.3012	9.8489

Step 2: Establish the global weak ranking (WR).

Based on the Eq. (15), compute the global weak ranking $R\left( {X_{ij} } \right)$.

Step 3: Compute the weak ranking of $X_{i} \big( i = 1,2, \cdots ,5 \big)$.

Based on the Eq. (16), $\tilde{R}\left( {X_{1} } \right) = 81.5379$, $\tilde{R}\left( {X_{2} } \right) = 78.6293$, $\tilde{R}\left( {X_{3} } \right) = 85.5630$, $\tilde{R}\left( {X_{4} } \right) = 84.0396$, $\tilde{R}\left( {X_{5} } \right) = 83.5090$.

Then, the WR can be obtained as follows:

$$ X_{3} \succ X_{5} \succ X_{4} \succ \, X_{1} \succ X_{2} $$

Step 4: Obtain the preference intensity (PI).

By applying Eq. (17), the average PI of $X_{i}$ over $X_{\kappa }$ with respect to $C_{j}$ can be obtained, which shown in Table 17.

**Table 17:** The average PI of $X_{i}$ over $X_{\kappa }$ with respect to $C_{j}$

$\tilde{P}\left( {X_{i} ,X_{\kappa } } \right)$	$\tilde{P}\left( {X_{1} ,X_{\kappa } } \right)$	$\tilde{P}\left( {X_{2} ,X_{\kappa } } \right)$	$\tilde{P}\left( {X_{3} ,X_{\kappa } } \right)$	$\tilde{P}\left( {X_{4} ,X_{\kappa } } \right)$	$\tilde{P}\left( {X_{5} ,X_{\kappa } } \right)$
$X_{1}$	0.0000	0.0050	0.0032	0.0047	0.0062
$X_{2}$	0.0016	0.0000	0.0011	0.0017	0.0026
$X_{3}$	0.0083	0.0100	0.0000	0.0056	0.0056
$X_{4}$	0.0079	0.0086	0.0037	0.0000	0.0048
$X_{5}$	0.0087	0.0089	0.0031	0.0035	0.0000

By applying Eq. (18), the net PI of $X_{i}$ over $X_{\kappa }$ with respect to $C_{j}$ can be acquired, which shown in Table 18.

**Table 18:** The net PI of $X_{i}$ over $X_{\kappa }$ with respect to $C_{j}$

$\Delta \tilde{P}\left( {X_{i} ,X_{\kappa } } \right)$	$\Delta \tilde{P}\left( {X_{1} ,X_{\kappa } } \right)$	$\Delta \tilde{P}\left( {X_{2} ,X_{\kappa } } \right)$	$\Delta \tilde{P}\left( {X_{3} ,X_{\kappa } } \right)$	$\Delta \tilde{P}\left( {X_{4} ,X_{\kappa } } \right)$	$\Delta \tilde{P}\left( {X_{5} ,X_{\kappa } } \right)$
$X_{1}$	0.0000	0.0034	− 0.0006	− 0.0032	− 0.0025
$X_{2}$	− 0.0034	0.0000	− 0.0089	− 0.0069	− 0.0027
$X_{3}$	0.0006	0.0089	0.0000	0.0019	0.0025
$X_{4}$	0.0032	0.0069	− 0.0019	0.0000	0.0013
$X_{5}$	0.0025	0.0027	− 0.0025	− 0.0013	0.0000

Step 5: Construct the preference/indifference/incomparability (PIR) structure.

Let $\mu = 0.001$, $\theta = 0.2$ and $\vartheta = 4$, based on Eq. (19) and Eq. (20), the PIR structure is determined as shown in Table 19.

**Table 19:** The PIR structure of pairwise cities

PIR	$X_{1}$	$X_{2}$	$X_{3}$	$X_{4}$	$X_{5}$
$X_{1}$	–	$<$	$>$	$>$	$>$
$X_{2}$	$>$	–	$>$	$>$	$>$
$X_{3}$	$<$	$<$	–	$>$	$>$
$X_{4}$	$<$	$<$	$<$	–	$>$
$X_{5}$	$<$	$<$	$<$	$<$	–

Step 6: Determine the strong ranking based on the weak ranking and PIR.

The WR can be obtained as follows:

$$ X_{2} \succ X_{1} \succ X_{3} \succ \, X_{4} \succ X_{5} $$

Actually, it is intuitive to see the ranking result based on the IT2F-OREST method is coordinate with the real situation, and the ranking result based on the traditional ORESTE method is contrary to the real situation. Therefore, the developed IT2F-OREST method is more reliable. In theory, compared with the traditional OREST method, the developed IT2F-OREST method has the following four advantages:

1. The decision matrixes obtained by applying the developed IT2F-OREST method can gather as much evaluation information as possible from experts by taking advantage of IT2FS. But the traditional OREST method matrix is simply expressed by ranking and it is very hard for experts to reach a consensus on the rankings.
2. Comparing with the traditional OREST method, which the ranking is simply based on the distance measure, the developed IT2F-OREST method can more effectively reflect the conflicts between IT2FSs by the distance measure and likelihood measure.
3. Comparing with the traditional OREST method, the developed IT2F-OREST method is more reasonable and reliable by developing the PI indifference threshold $\mu$ based on the IT2FS indifference threshold.
4. About the calculation process, the developed IT2F-OREST method is more practical and flexible than the traditional OREST method. Because when a new alternative is added, the traditional OREST method have to re-adjust the ranking and recalculate the preference scores of all the alternatives but the developed IT2F-OREST method simply needs to calculate the preference scores of the added new alternative.

### Comparison analyses with the other interval type-2 fuzzy MCDM method

Next, for demonstrating in further detail, the superiority of the developed IT2F-OREST method, Mathew et al.’ IT2F- TOPSIS method [17], Wu et al.’ IT2F- VIKOR method [18], as well as Wang et al.’ IT2F-MULTIMOORA method [19] are applied to solve the above-mentioned case of RER under COVID-19 epidemic stress. For guaranteeing the consistency of all the above-mentioned MADM methods, the distance measure based on Eq. (10) are applying to obtain the distance between the IT2FSs.

#### Comparing with the IT2F-TOPSIS method

TOPSIS method is a more popular MADM method based on the utility value theory [17]. First, based on Eq. (10), the negative ideal separation matrix is built by computing $d\left( {\tilde{A}_{ij} ,\tilde{A}_{{_{j} }}^{ - } } \right)$, where $\tilde{A}_{{_{j} }}^{ - }$ is the worst $\tilde{A}_{ij}$, which is shown in Table 20. In addition, the positive ideal separation matrix has been built, which is the same as Tables 8.

**Table 20:** The $d\left( {\tilde{A}_{ij} ,\tilde{A}_{{_{j} }}^{ - } } \right)$ between each city and worst one on each indicator

$d\left( {\tilde{A}_{ij} ,\tilde{A}_{{_{j} }}^{ - } } \right)$	$C_{1}$	$C_{2}$	$C_{3}$	$C_{4}$	$C_{5}$	$C_{6}$	$C_{7}$
$X_{1}$	0.9634	0.3456	0.1600	0.2110	0.4221	0.4674	0.3926
$X_{2}$	0.6867	0.0000	0.2400	0.0000	0.1964	0.1600	0.5454
$X_{3}$	0.2942	0.1930	0.0000	0.0369	0.1527	0.1600	0.4363
$X_{4}$	0.0000	0.0800	0.1600	0.0400	0.0000	0.0000	0.5054
$X_{5}$	0.0000	0.0000	0.1970	0.0738	0.2763	0.1600	0.0000

$d\left( {\tilde{A}_{ij} ,\tilde{A}_{{_{j} }}^{ - } } \right)$	$C_{8}$	$C_{9}$	$C_{10}$	$C_{11}$	$C_{12}$	$C_{13}$	$C_{14}$
$X_{1}$	0.1165	0.4654	0.1899	0.0000	0.0000	0.0000	0.4854
$X_{2}$	0.1930	0.3526	0.2665	0.1600	0.1200	0.2000	0.4317
$X_{3}$	0.0000	0.0766	0.0000	0.3927	0.0400	0.3163	0.4652
$X_{4}$	0.1165	0.0766	0.0766	0.4259	0.1564	0.3528	0.3528
$X_{5}$	0.0400	0.0000	0.0369	0.4658	0.1930	0.4259	0.0000

Next, compute the relative closeness $R_{1} \left( {X_{i} } \right)$ based on Eq. (38).

38 $$ \begin{aligned} R_{1} \left( {X_{i} } \right) & = \frac{{\sum\nolimits_{j = 1}^{14} {\omega_{j} d\left( {\tilde{A}_{ij} ,\tilde{A}_{{_{j} }}^{ - } } \right)} }}{{\sum\nolimits_{j = 1}^{14} {\omega_{j} d\left( {\tilde{A}_{ij} ,\tilde{A}_{{_{j} }}^{ - } } \right)} + \sum\nolimits_{j = 1}^{14} {\omega_{j} d\left( {\tilde{A}_{ij} ,\tilde{A}_{{_{j} }}^{ + } } \right)} }}\\ & \quad \left( {i = 1,2, \cdots ,5} \right). \end{aligned} $$

The detailed numerical results derived by the IT2F-TOPSIS method is shown in Table 21.

**Table 21:** The detailed numerical results derived by the IT2F- TOPSIS method

	$\sum\nolimits_{j = 1}^{14} {\omega_{j} d\left( {\tilde{A}_{ij} ,\tilde{A}_{{_{j} }}^{ - } } \right)}$	$\sum\nolimits_{j = 1}^{14} {\omega_{j} d\left( {\tilde{A}_{ij} ,\tilde{A}_{{_{j} }}^{ + } } \right)}$	$R_{1} \left( {X_{i} } \right)$	Rank
$X_{1}$	0.2986	0.1234	0.7075	1
$X_{2}$	0.2526	0.1705	0.5970	2
$X_{3}$	0.1815	0.2426	0.4280	3
$X_{4}$	0.1577	0.2823	0.3584	4
$X_{5}$	0.1562	0.2851	0.3539	5

In final, rank the cities based on the $R_{1} \left( {X_{i} } \right)$ and get $X_{1} \succ X_{2} \succ X_{3} \succ \, X_{4} \succ X_{5}$.

Though the ranking result based on the IT2F-TOPSIS method is the same as the developed IT2F-OREST, the IT2F-TOPSIS method lose sight of the PIR relations. That is to say, the IT2F-TOPSIS method cannot takes into consideration their relationships flexibly, but in the developed IT2F-OREST method, some errors in the process of evaluation can be noticed by applying different thresholds. Generally, as the threshold changes, the difference between indicators may become smaller and smaller. In addition, in the IT2F-TOPSIS method, the relationships are strictly examined by the preference scores. If only the preference scores are the same, there is only indifference relationships, and if the preference are not same, there is a preference relationship.

#### Comparing with the IT2F-VIKOR method

VIKOR method as a classic MADM method to integrating utility values is widely recognized [18]. However, it has to be centered on the compromise solution. Let $\psi = 0.5$ denotes the strategy of the maximum group utility. Next, compute the relative closeness $R_{2} \left( {X_{i} } \right)$ based on Eq. (39).

39 $$ R_{2} \left( {X_{i} } \right) = \psi \left( {\sum\nolimits_{j = 1}^{14} {\omega_{j} d\left( {\tilde{A}_{ij} ,\tilde{A}_{{_{j} }}^{ + } } \right)} } \right) + \left( {1 - \psi } \right)\sum\nolimits_{j = 1}^{14} {\omega_{j} d\left( {\tilde{A}_{ij} ,\tilde{A}_{{_{j} }}^{ - } } \right)} \left( {i = 1,2, \cdots ,5} \right). $$

The detailed numerical results derived by the IT2F-VIKOR method is shown in Table 22.

**Table 22:** The detailed numerical results derived by the IT2F-TOPSIS method ($\psi = 0.5$)

	$\sum\nolimits_{j = 1}^{14} {\omega_{j} d\left( {\tilde{A}_{ij} ,\tilde{A}_{{_{j} }}^{ - } } \right)}$	$\sum\nolimits_{j = 1}^{14} {\omega_{j} d\left( {\tilde{A}_{ij} ,\tilde{A}_{{_{j} }}^{ + } } \right)}$	$R_{1} \left( {X_{i} } \right)$	Rank
$X_{1}$	0.2986	0.1234	0.2110	4
$X_{2}$	0.2526	0.1705	0.2115	5
$X_{3}$	0.1815	0.2426	0.2121	3
$X_{4}$	0.1577	0.2823	0.2200	2
$X_{5}$	0.1562	0.2851	0.2206	1

In final, rank the cities based on the $R_{2} \left( {X_{i} } \right)$ and get $X_{5} \succ X_{4} \succ X_{3} \succ \, X_{1} \succ X_{2}$.

Obviously, the ranking result based on the IT2F-VIKOR method is the exact opposite of the ranking result based on the developed IT2F-OREST method. Compared with the new method, IT2F-VIKOR method widens the differences between two cities by regret measure and only divides the relationships into PI relation. In addition, it is difficult to obtain the strategy of the maximum group utility reasonably, but in real case, it has essential effect toward the ranking result.

#### Comparing with the IT2F-MULTIMOORA method

The MULTIMOORA method is a multiple objectives optimization method, which contains the ratio system, the reference point method and the full multiplicative form method [19].

First, based on Eq. (40) and the aggregate results in the step 5 of Phase I (“Solving the case by the developed IT2F-ORESTE method”), the normalization decision matrix is constructed.

40 $$ \begin{gathered} \overline{\tilde{A}}_{ij} = \left[ {\left( {\frac{{\alpha_{ij1}^{L} }}{{g_{j} }},\frac{{\alpha_{ij2}^{L} }}{{g_{j} }},\frac{{\alpha_{ij3}^{L} }}{{g_{j} }},\frac{{\alpha_{ij4}^{L} }}{{g_{j} }};h_{{\tilde{A}_{ij} }}^{L} } \right),\left( {\frac{{\alpha_{ij1}^{U} }}{{g_{j} }},\frac{{\alpha_{ij2}^{U} }}{{g_{j} }},\frac{{\alpha_{ij3}^{U} }}{{g_{j} }},\frac{{\alpha_{ij4}^{U} }}{{g_{j} }};h_{{\tilde{A}_{ij} }}^{U} } \right)} \right]\\ \quad \left( {i = 1,2, \cdots ,5;j = 1,2, \cdots ,14} \right) \hfill \\ = \left[ {\left( {\overline{\alpha }_{ij1}^{L} ,\overline{\alpha }_{ij2}^{L} ,\overline{\alpha }_{ij3}^{L} ,\overline{\alpha }_{ij4}^{L} ;h_{{\tilde{A}_{ij} }}^{L} } \right),\left( {\overline{\alpha }_{ij1}^{L} ,\overline{\alpha }_{ij2}^{L} ,\overline{\alpha }_{ij3}^{L} ,\overline{\alpha }_{ij4}^{L} ;h_{{\tilde{A}_{ij} }}^{U} } \right)} \right] \hfill \\ \end{gathered} $$

In which, the parameter $g_{j} = \sqrt {\sum\nolimits_{i = 1}^{5} {\sum\nolimits_{\xi = 1}^{4} {\left( {\alpha_{ij\xi }^{L} } \right)^{2} + \sum\nolimits_{i = 1}^{5} {\sum\nolimits_{\xi = 1}^{4} {\left( {\alpha_{ij\xi }^{U} } \right)^{2} } } } } }$.

Second, because of each indicator corresponds to benefit type, based on the Eq. (41), the ration of each city can be calculated:

41 $$ \Re_{1} \left( {X_{i} } \right) = \sum\limits_{j = 1}^{14} {\omega_{j} \overline{\tilde{A}}_{ij} } . $$

The numerical results are:

$$\begin{aligned}
\Re_{1} \left( {X_{1} } \right) & = \left[ \left( {0.1515,0.2026,0.2026,0.2405;1} \right),\right.\\
& \quad \left. \left( {0.1771,0.2026,0.2026,0.2215;0.9} \right) \right]
\end{aligned} $$

$$ \begin{aligned}
\Re_{1} \left( {X_{2} } \right) &= \left[ \left( {0.1307,0.1866,0.1866,0.2378;1} \right),\right.\\
&\quad \left.\left( {0.1586,0.1866,0.1866,0.2122;0.9} \right) \right]
\end{aligned} $$

$$ \begin{aligned}
\Re_{1} \left( {X_{3} } \right) &= \left[ \left( {0.1071,0.1630,0.1630,0.2156;1} \right),\right.\\
&\quad \left.\left( {0.1350,0.1630,0.1630,0.1893;0.9} \right) \right]
\end{aligned} $$

$$ \begin{aligned}
&\Re_{1} \left( {X_{4} } \right) = \left[ \left( {0.1049,0.1548,0.1548,0.2025;1} \right),\right.\\
&\quad \left.\left( {0.1298,0.1548,0.1548,0.1911;0.9} \right) \right]
\end{aligned} $$

$$ \begin{aligned}
\Re_{1} \left( {X_{5} } \right) &= \left[ \left( {0.1065,0.1555,0.1555,0.2027;1} \right),\right.\\
&\quad \left. \left( {0.1310,0.1555,0.1555,0.1916;0.9} \right) \right]
\end{aligned}$$

Based on the definition 7, the ranking result is: $X_{1} \succ X_{2} \succ X_{3} \succ \, X_{4} \succ X_{5}$.

Third, by using the data in Tables 5 and 11, based on Eq. (42), the reference point can be calculated:

42 $$ \Re_{2} \left( {X_{i} } \right) = \mathop {\max \omega_{j} }\limits_{j} \left( {{{d\left( {\tilde{A}_{ij} ,\tilde{A}_{{_{j} }}^{ + } } \right)} \mathord{\left/ {\vphantom {{d\left( {\tilde{A}_{ij} ,\tilde{A}_{{_{j} }}^{ + } } \right)} {d\left( {\tilde{A}_{ij} ,\tilde{A}_{{_{j} }}^{ - } } \right)}}} \right. \kern-\nulldelimiterspace} {d\left( {\tilde{A}_{ij} ,\tilde{A}_{{_{j} }}^{ - } } \right)}}} \right). $$

The numerical results are:

$$\Re_{2} \left( {X_{1} } \right) = 0.0711$$,$$\Re_{2} \left( {X_{2} } \right) = 0.2100$$,$$\Re_{2} \left( {X_{3} } \right) = 0.3766$$,$$\Re_{2} \left( {X_{4} } \right) = 0.4082$$,$$\Re_{2} \left( {X_{5} } \right) = 1.0141$$.

And the ranking result is: $X_{5} \succ X_{4} \succ X_{3} \succ \, X_{2} \succ X_{1}$.

Next, based on the Eq. (43), the full multiplicative form method can be calculated:

43 $$ \Re_{3} \left( {X_{i} } \right) = {\raise0.7ex\hbox{${\prod\nolimits_{j = 1}^{\vartheta } {\left( {\overline{\tilde{A}}_{ij} } \right)^{{\omega_{j} }} } }$} \!\mathord{\left/ {\vphantom {{\prod\nolimits_{j = 1}^{\vartheta } {\left( {\overline{\tilde{A}}_{ij} } \right)^{{\omega_{j} }} } } {\prod\nolimits_{j = \vartheta + 1}^{14} {\left( {\overline{\tilde{A}}_{ij} } \right)^{{\omega_{j} }} } }}}\right.\kern-\nulldelimiterspace} \!\lower0.7ex\hbox{${\prod\nolimits_{j = \vartheta + 1}^{14} {\left( {\overline{\tilde{A}}_{ij} } \right)^{{\omega_{j} }} } }$}}, $$

where $\prod\nolimits_{j = 1}^{\vartheta } {\left( {\overline{\tilde{A}}_{ij} } \right)^{{\omega_{j} }} }$ is the product of the weighted normalized scores of all benefit criteria and $\prod\nolimits_{j = \vartheta + 1}^{14} {\left( {\overline{\tilde{A}}_{ij} } \right)^{{\omega_{j} }} }$ is the product of the weighted normalized scores of all cost criteria.

The numerical results are:

$$ \begin{aligned}
\Re_{3} \left( {X_{1} } \right) = \left[ \left( {0.1336,0.1913,0.1913,0.2345;1} \right),\right.\\
\left.\left( {0.1632,0.1913,0.1913,0.2134;0.9} \right) \right]
\end{aligned} $$

$$ \begin{aligned}
&\Re_{3} \left( {X_{2} } \right) = \left[ \left( {0.1276,0.1842,0.1842,0.2360;1} \right),\right.\\
&\quad \left.\left( {0.1560,0.1842,0.1842,0.2102;0.9} \right) \right]
\end{aligned} $$

$$ \begin{aligned}
&\Re_{3} \left( {X_{3} } \right) = \left[ \left( {0.0992,0.1596,0.1596,0.2141;1} \right),\right.\\
&\quad \left.\left( {0.1301,0.1596,0.1596,0.1870;0.9} \right) \right]
\end{aligned} $$

$$ \begin{aligned}
&\Re_{3} \left( {X_{1} } \right) = \left[ \left( {0.0000,0.0000,0.0000,0.1835;1} \right),\right.\\
&\quad \left.\left( {0.0000,0.0000,0.0000,0.1885;0.9} \right) \right]
\end{aligned}$$

$$ \begin{aligned}
&\Re_{3} \left( {X_{1} } \right) = \left[ \left( {0.0000,0.0000,0.0000,0.1820;1} \right),\right.\\
&\quad \left.\left( {0.0000,0.0000,0.0000,0.1866;0.9} \right) \right]
\end{aligned}$$

Based on the definition 7, the ranking result is: $X_{1} \succ X_{2} \succ X_{3} \succ \, X_{4} \succ X_{5}$.

Finally, by applying the above three method, the comprehensive ranking result under the IT2F-MULTIMOORA method is shown in Table 23, that is: $X_{1} \succ X_{2} \succ X_{3} \succ \, X_{4} \succ X_{5}$.

**Table 23:** The comprehensive ranking result under the IT2F-MULTIMOORA method

	Ration system	Reference point	Full multiplicative	Final ranking
$X_{1}$	1	5	1	1
$X_{2}$	2	4	2	2
$X_{3}$	3	3	3	3
$X_{4}$	4	2	4	4
$X_{5}$	5	1	5	5

The IT2F-MULTIMOORA method takes into consideration the ranking results of three methods synthetically. However, this synthetical method has no more detailed division as the developed IT2F-OREST method. Most importantly, if the three methods obtain completely different ranking results, it will be very hard to obtain the ultimate reasonable ranking result.

#### Comparison summary

Based on the above analyses, compared with the other three interval type-2 fuzzy MCDM methods, the developed IT2F-OREST method has the following special superiorities:

1. The developed IT2F-OREST method can give a detailed ranking result by using the PIR relationships among cities. Compared with above three methods, it can not only provide preference relationships, but also provide incomparable relationships and indifference relationships, which can reduce information distortion in the calculation and evaluation process. The results obtained by this method are more reliable and reasonable.
2. The application strategies obtained by the developed IT2F-OREST method are more realistic and practical. The ranking results can change with different thresholds. Therefore, decision maker can adjust the parameters based on the real situation and physical truth to obtain the comprehensive ranking results.
3. The preference index of each pair of alternatives is computed based on the likelihood of IT2FSs. In this method, the ranking results are acquired based on the comprehensive pairwise comparison of all alternatives, making them more precise and persuasive. In addition, the IT2F-OREST method are developed by applying the extended vertex method for distance measure, which is a simple expression that needs few calculations.
4. In addition, the developed IT2F-OREST method can solve the MCDM problems with the quantitative and qualitative indicators and the weights of indicators are unknown.

In detail, the comparisons over these methods are simplified as shown in Table 24.

**Table 24:** The comparisons over the four methods

	Criteria type	Theoretical basis	Relation
Traditional ORESTE	Quantitative	Rank	PIR
IT2F-TOPSIS	Qualitative	Distance	PI
IT2F-VIKOR	Qualitative	Distance	PI
IT2F-MULTIMOORA	Qualitative	Distance	PI
The proposed IT2F-ORESTE	Qualitative and Quantitative	Distance	PIR

### Practical implications

For improving the regional economic restorability, some valuable practical implications based on the above evaluation results are suggested as follows:

1. The administrative department should speed up the improvement of the emergency support system, take the emergency supplies support as an important part, and establish a corresponding reserve of emergency supplies. Relevant working mechanisms and emergency plans should be formulated in accordance with the principle of centralized management and unified allocation, and effectively improve emergency response capabilities.
2. After the COVID-19 outbreak is effectively contained, it is important to resume work and production in a timely manner to promote stable economic and social development. On one hand, the production order should be restored in different areas by zoning and grading, on the other hand, the resumption of work and production across the industrial chain need to be promoted.
3. The government should encourage commercial banks to speed up innovation and upgrading of financial services, and make full use of technologies such as big data, blockchain and artificial intelligence to speed up digital transformation. On the supply side, the supervision council will guide financial institutions to increase credit supply and provide greater support to the real economy, especially to enterprises heavily affected by the epidemic. On the demand side, the administration need to effectively expand domestic demand and make up for the shortage of external demand to help enterprises, especially foreign trade enterprises, effectively cope with the impact of the epidemic.

## Conclusions

COVID-19 pandemic is considered to be the notorious economic shock arising throughout the year 2020. As a result, although the negative effects of the deadly CODIV-19 pandemic are still presented currently, the regional economic recovery phase must be projected to start due to the fact that regional economic development has plummeted to historic bottom. Quantitative research on the impact of major public health events on economic system can provide scientific support for improving the RER. In this paper, by developing an improved IT2F-ORESTE method based on the DM and likelihood of IT2FS, RER of different regions under the stress of COVID-19 are determined. First, some formulas are developed to convert quantitative PVs to IT2FSs for combining the quantitative and qualitative indicator information. Then, the vertex method for DM is extended to encompass IT2FSs. Furthermore, a comprehensive discussion between the improved IT2F-ORESTE method, the traditional ORESTE method and two representative IT2F-MCDM methods, are developed to demonstrate the validity and reliability of the improved IT2F-ORESTE method. The case study presents a helpful reference for government departments to improve the RER.

The main contribution of this study to the current RER problem is the development of an improved IT2F-ORESTE method based on the DM and likelihood of IT2FS for dealing with RER assessment problem. Compared with current ORESTE method, the proposed method has some desirable capabilities for addressing MCDM-based RER problem. First, the proposed IT2F-OREST method can not only provide preference relationships, but also provide incomparable relationships and indifference relationships, which can reduce information distortion in the calculation and evaluation process. And it is developed by applying the extended vertex method for distance measure, which is a simple expression that needs few calculations. Second, decision maker can adjust the parameters based on the real situation and physical truth to obtain the comprehensive ranking results. More importantly, the proposed method can solve the MCDM problems with the quantitative and qualitative indicators and the weights of indicators are unknown.

There more or less are limitations existed in this study, which need to be remedied in the future. The optimum solution acquired by the proposed IT2F-ORESTE method may be inferior under certain criteria. In the future, the following aspects are worthy of further study. First, whether there are other meaningful indicators under more complex environments that can be included to improve indicator system is worthy of investigation. Second, in the process of evaluation, different experts may provide different ranking results, the consensus process among experts is a meaningful issue. Some recently developed consensus optimization models can be introduced to improve the performance of method. Last but not least, for improving reliability and accuracy, some other meaningful ORESTE method with different fuzzy information forms can be used to resolve RER problem [65, 66]. Third, the application scopes of the improved IT2F-ORESTE method will be extended further to include economic—ecological complex system restorability, regional economic competitiveness, enterprise sustainable development ability, etc.

## Data Availability

The authors confirm that the data supporting the findings of this study are available within the article.

## Appendix

See Tables 25, 26, 27, 28, 29, 30, 31, 32, 33, 34.

**Table 25:** Initial decision matrix $\overline{D}_{1}$ given by $E_{1}$

	$C_{2}$	$C_{3}$	$C_{4}$	$C_{5}$	$C_{6}$	$C_{7}$	$C_{8}$	$C_{9}$	$C_{10}$	$C_{11}$	$C_{12}$	$C_{13}$	$C_{14}$
$X_{1}$	VS	S	ES	M	S	VS	S	S	VS	M	S	M	S
$X_{2}$	S	S	S	M	M	VS	VS	S	VS	M	S	M	M
$X_{3}$	VS	W	M	M	S	S	S	M	M	S	S	VS	S
$X_{4}$	S	W	W	VW	W	M	S	M	VS	VS	S	S	S
$X_{5}$	M	VW	M	S	M	W	M	M	S	VS	VS	VS	VW

**Table 26:** Initial decision matrix $\overline{D}_{2}$ given by $E_{2}$

	$C_{2}$	$C_{3}$	$C_{4}$	$C_{5}$	$C_{6}$	$C_{7}$	$C_{8}$	$C_{9}$	$C_{10}$	$C_{11}$	$C_{12}$	$C_{13}$	$C_{14}$
$X_{1}$	ES	M	VS	VS	ES	S	S	VS	M	M	W	M	ES
$X_{2}$	S	S	M	S	M	S	S	VS	VS	M	S	M	VS
$X_{3}$	S	M	S	VS	S	S	M	W	W	S	S	S	S
$X_{4}$	S	M	S	M	M	S	VS	W	S	VS	S	VS	S
$X_{5}$	M	W	VS	S	M	VW	M	W	VS	VS	S	VS	VW

**Table 27:** Initial decision matrix $\overline{D}_{3}$ given by $E_{3}$

	$C_{2}$	$C_{3}$	$C_{4}$	$C_{5}$	$C_{6}$	$C_{7}$	$C_{8}$	$C_{9}$	$C_{10}$	$C_{11}$	$C_{12}$	$C_{13}$	$C_{14}$
$X_{1}$	ES	M	S	VS	VS	M	S	VS	M	W	M	W	VS
$X_{2}$	S	M	S	W	M	S	VS	S	VS	M	S	S	S
$X_{3}$	S	W	M	M	M	S	M	W	S	S	M	S	S
$X_{4}$	M	M	VS	M	W	VS	M	M	M	VS	S	VS	M
$X_{5}$	S	VS	VS	S	M	W	S	VW	W	VS	S	VS	W

**Table 28:** Initial decision matrix $\overline{D}_{4}$ given by $E_{4}$

	$C_{2}$	$C_{3}$	$C_{4}$	$C_{5}$	$C_{6}$	$C_{7}$	$C_{8}$	$C_{9}$	$C_{10}$	$C_{11}$	$C_{12}$	$C_{13}$	$C_{14}$
$X_{1}$	VS	M	VS	VS	VS	M	M	S	VS	W	M	M	S
$X_{2}$	M	S	VS	S	S	VS	S	S	VS	M	S	S	VS
$X_{3}$	VS	M	VS	W	M	S	S	M	M	VS	M	S	VS
$X_{4}$	S	S	S	W	M	VS	S	W	S	VS	S	S	S
$X_{5}$	S	VS	VS	S	S	W	S	M	S	S	S	S	M

**Table 29:** Initial decision matrix $\overline{D}_{5}$ given by $E_{5}$

	$C_{2}$	$C_{3}$	$C_{4}$	$C_{5}$	$C_{6}$	$C_{7}$	$C_{8}$	$C_{9}$	$C_{10}$	$C_{11}$	$C_{12}$	$C_{13}$	$C_{14}$
$X_{1}$	VS	M	VS	VS	ES	S	VS	VS	VS	W	M	W	S
$X_{2}$	W	M	S	S	M	VS	S	M	M	S	S	S	VS
$X_{3}$	S	W	VS	M	M	S	M	M	S	VS	S	S	VS
$X_{4}$	S	S	M	M	W	VS	S	M	W	M	VS	S	S
$X_{5}$	M	S	M	M	S	W	S	W	W	VS	VS	VS	M

**Table 30:** The initial linguistic PRs matrix $\overline{D}_{L1}$ given by $E_{1}$

PD	$C_{1}$	$C_{2}$	$C_{3}$	$C_{4}$	$C_{5}$	$C_{6}$	$C_{7}$	$C_{8}$	$C_{9}$	$C_{10}$	$C_{11}$	$C_{12}$	$C_{13}$	$C_{14}$
$C_{1}$	O	W	M	M	S	VS	VS	S	W	VW	W	M	S	ES
$C_{2}$	S	O	M	S	S	S	VS	VS	VS	M	M	VW	VW	VS
$C_{3}$	M	M	O	EW	VW	VS	S	ES	M	EW	ES	VS	S	S
$C_{4}$	M	W	ES	O	M	VS	W	VW	VS	S	M	ES	VS	W
$C_{5}$	W	W	VS	M	O	W	S	VS	W	S	S	M	VS	S
$C_{6}$	VW	W	VW	VW	S	O	VS	VS	VS	VS	EW	W	VS	VW
$C_{7}$	VW	VW	W	S	W	VW	O	VS	W	VW	W	S	M	ES
$C_{8}$	W	VW	EW	VS	VW	VW	VW	O	VW	M	ES	EW	W	M
$C_{9}$	S	VW	M	VW	S	VW	S	VS	O	VS	S	VS	M	W
$C_{10}$	VS	M	ES	W	W	VW	S	M	VW	O	VS	ES	VS	S
$C_{11}$	S	M	EW	M	W	ES	VS	EW	W	VW	O	M	S	W
$C_{12}$	M	VS	VW	EW	M	S	W	ES	VW	EW	M	O	VW	S
$C_{13}$	W	VS	W	VW	VW	VW	M	S	M	VW	W	VS	O	W
$C_{14}$	EW	VW	W	S	W	VS	EW	M	S	W	S	W	S	O

**Table 31:** The initial linguistic PRs matrix $\overline{D}_{L2}$ given by $E_{2}$

PD	$C_{1}$	$C_{2}$	$C_{3}$	$C_{4}$	$C_{5}$	$C_{6}$	$C_{7}$	$C_{8}$	$C_{9}$	$C_{10}$	$C_{11}$	$C_{12}$	$C_{13}$	$C_{14}$
$C_{1}$	O	VW	EW	M	VW	ES	S	ES	VW	M	S	VS	S	W
$C_{2}$	VS	O	S	M	M	VS	ES	W	S	VS	S	W	S	VW
$C_{3}$	ES	W	O	VW	EW	W	S	VW	VW	VS	W	S	M	VW
$C_{4}$	M	M	VS	O	VS	M	W	W	M	S	S	S	M	S
$C_{5}$	VS	M	ES	VW	O	VS	VW	S	EW	W	VW	W	M	W
$C_{6}$	EW	VW	S	M	VW	O	S	W	S	EW	W	VS	S	VS
$C_{7}$	W	EW	W	S	VS	W	O	S	VS	M	ES	EW	W	W
$C_{8}$	EW	S	VS	S	W	S	W	O	W	S	EW	S	W	W
$C_{9}$	VS	W	VS	M	ES	W	VW	S	O	M	VS	S	W	VW
$C_{10}$	M	VW	VW	W	S	ES	M	W	M	O	VW	VS	M	W
$C_{11}$	W	W	S	W	VS	S	EW	ES	VW	VS	O	VW	S	M
$C_{12}$	VW	S	W	W	S	VW	ES	W	W	VW	VS	O	S	ES
$C_{13}$	W	W	M	M	M	W	S	S	S	M	W	W	O	S
$C_{14}$	S	VS	VS	W	S	VW	S	S	VS	S	M	EW	W	O

**Table 32:** The initial linguistic PRs matrix $\overline{D}_{L3}$ given by $E_{3}$

PD	$C_{1}$	$C_{2}$	$C_{3}$	$C_{4}$	$C_{5}$	$C_{6}$	$C_{7}$	$C_{8}$	$C_{9}$	$C_{10}$	$C_{11}$	$C_{12}$	$C_{13}$	$C_{14}$
$C_{1}$	O	VS	S	VS	VW	S	EW	M	S	W	S	S	M	ES
$C_{2}$	VW	O	M	M	M	W	ES	VS	VW	VW	W	W	ES	VW
$C_{3}$	W	M	O	W	S	M	ES	S	M	S	M	S	W	S
$C_{4}$	VW	M	S	O	VS	VW	S	ES	VS	VW	EW	S	S	ES
$C_{5}$	VS	M	W	VW	O	M	W	M	W	VS	ES	VW	ES	W
$C_{6}$	W	S	M	VS	M	O	M	ES	EW	S	S	S	S	S
$C_{7}$	ES	EW	EW	W	S	M	O	M	S	VS	VS	VW	W	W
$C_{8}$	M	VW	W	EW	M	EW	M	O	M	S	VW	S	W	M
$C_{9}$	W	VS	M	VW	S	ES	W	M	O	EW	VS	VW	VW	S
$C_{10}$	S	VS	W	VS	VW	W	VW	W	ES	O	S	W	VS	M
$C_{11}$	W	S	M	ES	EW	W	VW	VS	VW	W	O	S	M	VS
$C_{12}$	W	S	W	W	VS	W	VS	W	VS	S	W	O	M	S
$C_{13}$	M	EW	S	W	EW	W	S	S	VS	VW	M	M	O	EW
$C_{14}$	EW	VS	W	EW	S	W	S	M	W	M	VW	W	ES	O

**Table 33:** The initial linguistic PRs matrix $\overline{D}_{L4}$ given by $E_{4}$

PD	$C_{1}$	$C_{2}$	$C_{3}$	$C_{4}$	$C_{5}$	$C_{6}$	$C_{7}$	$C_{8}$	$C_{9}$	$C_{10}$	$C_{11}$	$C_{12}$	$C_{13}$	$C_{14}$
$C_{1}$	O	M	W	S	VW	M	S	ES	EW	VS	ES	S	M	M
$C_{2}$	M	O	M	EW	M	ES	VW	M	W	W	M	VS	M	M
$C_{3}$	S	M	O	VS	M	VS	M	W	VS	VW	S	M	W	VW
$C_{4}$	W	ES	VW	O	S	S	VW	S	M	VS	W	VW	ES	EW
$C_{5}$	VS	M	M	W	O	S	ES	M	S	EW	M	M	VW	M
$C_{6}$	M	EW	VW	W	W	O	VW	S	S	VW	VS	VW	W	VS
$C_{7}$	EW	VS	M	VS	EW	VS	O	M	M	S	W	S	S	W
$C_{8}$	ES	M	S	W	M	W	M	O	S	W	ES	S	EW	S
$C_{9}$	VW	S	VW	M	W	W	M	W	O	M	M	S	M	S
$C_{10}$	EW	S	VS	VW	ES	VS	W	S	M	O	S	S	EW	W
$C_{11}$	VS	M	W	S	M	VW	S	EW	M	W	O	ES	W	VS
$C_{12}$	W	VW	M	VS	M	VS	W	W	W	W	EW	O	VS	S
$C_{13}$	M	M	S	EW	VS	S	W	ES	M	ES	S	VW	O	VW
$C_{14}$	M	M	VS	ES	M	VW	S	W	W	S	VW	W	VS	O

**Table 34:** The initial linguistic PRs matrix $\overline{D}_{L5}$ given by *E*_5_

PD	$C_{1}$	$C_{2}$	$C_{3}$	$C_{4}$	$C_{5}$	$C_{6}$	$C_{7}$	$C_{8}$	$C_{9}$	$C_{10}$	$C_{11}$	$C_{12}$	$C_{13}$	$C_{14}$
$C_{1}$	O	ES	W	VW	W	M	S	W	S	M	W	W	ES	W
$C_{2}$	EW	O	M	M	S	EW	EW	S	S	S	VS	EW	S	M
$C_{3}$	W	M	O	S	VS	S	S	ES	W	VS	W	S	ES	ES
$C_{4}$	VS	M	W	O	VS	VW	M	S	VS	W	ES	VS	VS	VW
$C_{5}$	W	W	VW	VW	O	ES	W	VW	W	M	EW	S	W	W
$C_{6}$	M	ES	W	VS	EW	O	VS	VW	ES	M	W	S	M	M
$C_{7}$	W	ES	W	M	S	VW	O	ES	S	M	S	VW	S	ES
$C_{8}$	S	W	EW	W	VS	VS	EW	O	W	VW	EW	EW	S	W
$C_{9}$	W	W	W	VW	EW	EW	W	S	O	M	W	VS	M	S
$C_{10}$	M	W	VW	S	M	M	M	VS	M	O	S	S	S	M
$C_{11}$	S	VW	S	EW	ES	S	W	ES	S	W	O	VS	W	ES
$C_{12}$	S	ES	W	VW	W	W	VS	ES	VW	W	VW	O	EW	M
$C_{13}$	EW	W	EW	VW	S	M	W	W	M	W	S	ES	O	S
$C_{14}$	S	M	EW	VS	S	M	EW	S	W	M	EW	M	W	O
